# Comparative expression profiling of miRNA during anther development in genetic male sterile and wild type cotton

**DOI:** 10.1186/1471-2229-13-66

**Published:** 2013-04-19

**Authors:** Mingming Wei, Hengling Wei, Man Wu, Meizhen Song, Jinfa Zhang, Jiwen Yu, Shuli Fan, Shuxun Yu

**Affiliations:** 1 State Key Laboratory of Cotton Biology, Institute of Cotton Research of CAAS, Anyang, 455000 Henan, P. R. China; 2 College of Agronomy, Northwest A&F University, Yangling, 712100 Shaanxi, P. R. China; 3 Department of biology, New Mexico State University, Montreal 880033, USA

## Abstract

**Background:**

Genetic male sterility (GMS) in cotton (*Gossypium hirsutum*) plays an important role in the utilization of hybrid vigor. However, the molecular mechanism of the GMS is still unclear. While numerous studies have demonstrated that microRNAs (miRNA) regulate flower and anther development, whether different small RNA regulations exist in GMS and its wild type is unclear. A deep sequencing approach was used to investigate the global expression and complexity of small RNAs during cotton anther development in this study.

**Results:**

Three small RNA libraries were constructed from the anthers of three development stages each from fertile wild type (WT) and its GMS mutant cotton, resulting in nearly 80 million sequence reads. The total number of miRNAs and short interfering RNAs in the three WT libraries was significantly greater than that in the corresponding three mutant libraries. Sixteen conserved miRNA families were identified, four of which comprised the vast majority of the expressed miRNAs during anther development. In addition, six conserved miRNA families were significantly differentially expressed during anther development between the GMS mutant and its WT.

**Conclusions:**

The present study is the first to deep sequence the small RNA population in *G*. *hirsutum* GMS mutant and its WT anthers. Our results reveal that the small RNA regulations in cotton GMS mutant anther development are distinct from those of the WT. Further results indicated that the differently expressed miRNAs regulated transcripts that were distinctly involved in anther development. Identification of a different set of miRNAs between the cotton GMS mutant and its WT will facilitate our understanding of the molecular mechanisms for male sterility.

## Background

Cotton is one of the most important economic crops in the world. Male sterility is a simple and efficient pollination control system that has been widely used in hybrid cotton breeding. In cotton breeding, two major male sterile systems are used to produce hybrid seeds, namely cytoplasmic male sterility (CMS) and genetic male sterility (GMS). Both systems have a maternally (former) or nuclear (later) inherited trait that renders them inability to produce or release functional pollen, so they can be used as maternal plants to produce hybrid seeds. The molecular mechanism of male sterility is currently a research hotspot in plant science.

Many studies have demonstrated that CMS is often associated with unusual open reading frames (ORFs) found in mitochondrial genomes. For example, accumulation of the cytotoxic peptide ORF79 in Boro-Taichung (BT)-type cytoplasmic male sterile rice (*Oryza sativa*) with Chinsurah Boro II cytoplasm causes CMS. The ORF79 protein is expressed by a dicistronic gene, *atp6*-*orf79*, which exists in addition to the normal *atp6* gene in the BT-type mitochondrial genome [1]. Nuclear-encoded-fertility restorer genes can suppress CMS-inducing ORFs and restore male fertility [2]. GMS has been also extensively studied at the gene and protein expression levels with an exclusive focus on protein coding genes. Up to now, very few studies have been on the relationship between male sterility and protein non-coding genes.

As a class of non-coding genes, small non-coding RNAs (ncRNAs) play an essential role in regulating the molecular machinery of eukaryotic cells by controlling transcriptional and post-transcriptional mechanisms [3]. These processes include chromatin formation and maintenance, defense against selfish and parasitic entities such as transposable elements and viruses, as well as native protein coding gene expression [4,5]. Regulatory ncRNA in plants can be divided into two primary categories, i.e., microRNAs (miRNAs) and short interfering RNAs (siRNAs). While siRNAs result primarily from exogenous sources, miRNAs are a class of endogenous small regulatory ncRNAs with lengths ranging from 20–24 nucleotides (nt) that negatively regulate gene expression at the post-transcriptional level through perfect or near-perfect complementarity with target mRNAs for cleavage or inhibition of translation [6-8]. Some known miRNA loci form clusters in the genome and these miRNA clusters are probably produced by gene duplication and the miRNAs in a given cluster are often related to one another [9-11].

miRNAs are key post transcriptional regulators that control various biological and metabolic processes in eukaryotes, many of which are conserved and have more recently evolved species-specific diversity [12,13]. miRNAs also have important regulatory functions in specific biological processes during the life cycle of plants, such as controlling tissue differentiation and development, the phase switch from vegetative to reproductive growth, and responses to different biotic and abiotic stresses [14-16]. A growing number of new plant miRNAs have been identified in recent years. To date, more than 1,000 miRNAs have been annotated in Arabidopsis, rice, and other plant species [17]. However, the number of miRNAs in plants is apparently not saturated because new miRNAs are continually identified in different species. In Upland cotton (*G*. *hirsutum*), only 54 miRNAs have been reported.

Anthers are highly specialized organs for nutrient storage and reproductive development. Their maturation and development involves meticulous gene regulation at the transcriptional and post-transcriptional levels [18]. In anthers, small ncRNAs are essential for sporophyte development in the somatic diploid phase of flowering plants and small RNA pathways are present and functional in angiosperm male gametophytes [19,20]. In Arabidopsis, over-expression of miR167 leads to male sterility [21]. Even though there is no direct evidence that any miRNAs are causative genes for male sterility in plants, we hypothesize that differential expression of some miRNA genes are involved in regulation of male sterility.

As the first step towards the understanding of their regulatory mechanisms and networks of target genes in male sterility in plants, expression of miRNAs between a cotton GMS mutant (‘Dong A’) and its fertile wild type (WT) was compared using a deep sequencing approach developed by Solexa (Illumina Inc) in the present study. The male sterility of the GMS mutant ‘Dong A’ is controlled by one pair of recessive genes [22], and it has the same genetic background with its wild type (WT). Therefore, they are ideal genetic materials for studying cotton anther development and male sterility. In the present work, the expression patterns of miRNAs and the critical small RNA pathways of the GMS ‘Dong A’ and its WT were analyzed and compared at three different stages of male gametophyte development, followed by an integrated bioinformatics analysis to identify novel and candidate miRNAs. Furthermore, the expression profiles of miRNAs were analyzed by miRNA clustering, which has been widely used to study miRNA expression levels in various species [23-25]. By further comparing the expression patterns between selected miRNAs and their corresponding target genes, we have gained a better understanding of the molecular mechanism of miRNAs in anther development and genetic male sterility of cotton.

## Results

### Phenotypic analysis of impaired anthers in the cotton male-sterile mutant

**Figure 1:**
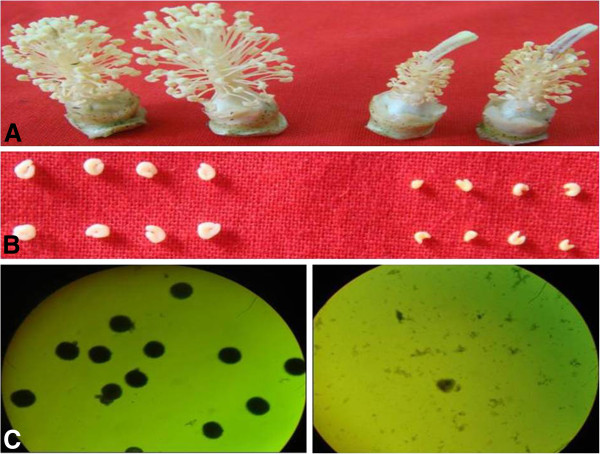
**Flowers and anthers of the WT and the GMS mutant.** From left to right: (**A**) Flower of *G*. *hirsutum* ‘Dong A’ (left) and GMS mutant of ‘Dong A’ (right); (**B**) anthers of the WT (left) and the GMS mutant (right) 1day post anthesis (DPA); (**C**) results of pollen stained with 2% I_2_-KI from 0 DPA flowers in the WT (left) and the GMS mutant (right) (10 × 40 view).

To determine the morphological defects of the cotton GMS mutant, we compared the anthers of the mutant and its fertile wild type (WT). At 0 day post anthesis (DPA), the mutant showed an abnormal floral phenotype with no pollen grains and smaller anthers than the WT (Figure 1A and B). The pollen grains in the WT and the mutant were stained with 2% I_2_-KI to detect starch activity during the flowering period. There were many viable pollen grains in the WT, while there was no viable pollen in the mutant observed (Figure 1C). Therefore, the mutant is completely sterile.

### Distribution of small RNAs during cotton anther development

Based on previous studies that the peak of male sterility mainly occurs in the uninucleate microspore stage of anthers in ‘Dong A’ GMS mutant [26], early anther development stages were chosen to identify possible miRNAs that may be involved in events leading to male sterility. In this study, anthers were selected from two earlier stages, i.e., meiosis stage (WT: Mar-F-1; mutant: Mar-S-1) and tetrad stage (WT: Mar-F-2; mutant: Mar-S-2), together with the uninucleate microspore stage (WT: Mar-F-3; mutant: Mar-S-3) from the GMS ‘Dong A’ mutant and its fertile wild type to construct six small RNA libraries (i.e., anthers from the three stages of the two genotypes).

The datasets from the six libraries were used to query the ncRNA sequences deposited in the National Center for Biotechnology Information Gene Bank (http://www.ncbi.nlm.nih.gov/) and the Rfam 9.1 database (http://rfam.janelia.org/) to separate the small RNAs that matched non-coding sequences, such as ribosomal RNA (rRNA), transfer RNA (tRNA), small nuclear RNA (snRNA), and small nucleolar RNA (snoRNA). The distribution of these fragments (<5% of the total reads) is listed in Table 1.

**Table 1:** Summary of small RNA sequences from the WT and the GMS mutant libraries

**Small RNA**	**Library**					
	**Mar**-**F**-**1**	**Mar**-**F**-**2**	**Mar**-**F**-**3**	**Mar**-**S**-**1**	**Mar**-**S**-**2**	**Mar**-**S**-**3**
**rRNA**	**308795**(**2**.**25**%)	**533737**(**3**.**26**%)	**185686**(**1**.**43**%)	**68630**(**0**.**63**%)	**236981**(**1**.**81**%)	**453064**(**3**.**34**%)
**tRNA**	**30091**(**0**.**22**%)	**92144**(**0**.**56**%)	**98208**(**0**.**76**%)	**12589**(**0**.**12**%)	**41200**(**0**.**31**%)	**116915**(**0**.**86**%)
**snoRNA**	**934**(**0**.**01**%)	**1198**(**0**.**01**%)	**836**(**0**.**01**%)	**466**(**0**%)	**839**(**0**.**01**%)	**1100**(**0**.**01**%)
**snRNA**	**2096**(**0**.**02**%)	**4793**(**0**.**03**%)	**3662**(**0**.**03**%)	**1060**(**0**.**01**%)	**2027**(**0**.**02**%)	**3248**(**0**.**02**%)
**Total reads**	**13741122**	**16347976**	**12981571**	**10839275**	**13106653**	**13570780**

**Figure 2:**
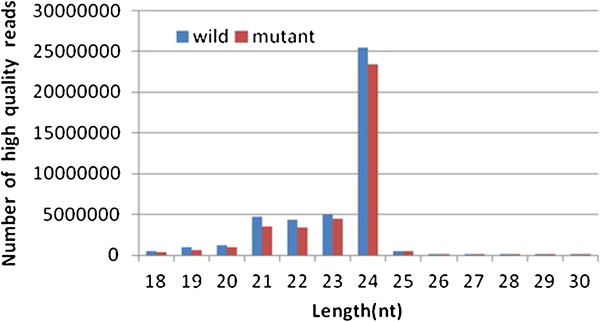
**Size distribution of small RNA sequences derived from the WT and mutant libraries.** Wild: the total small RNA sequences in the three WT libraries; Mutant: the total small RNA sequences in the three GMS mutant libraries. All reads were of high quality, ranging from 18–30 nt in length.

Almost 80 million small RNA sequences with lengths ranging from 18–30 nt were obtained in these six small RNA libraries. The majority of the small RNAs in both the WT and mutant libraries were 21–24 nt (Figure 2), which is within the typical size range for dicer-derived products and in agreement with most previously reported results. Of these, 24 nt small RNAs were the most abundant.

### Variations in small RNA expression in the WT and GMS mutant during anther development

The total numbers of miRNAs and siRNAs in the three WT libraries were greater than those of the three corresponding GMS mutant libraries (Additional file 1). The number of unique miRNAs in the three WT libraries was different from that in the three GMS mutant libraries. Moreover, the number of unique miRNAs in the Mar-F-1 library was twice that of the Mar-S-1 library and the number of unique siRNAs in the Mar-F-1 library was also significantly greater than that in the Mar-S-1 library (Additional file 1).

**Figure 3:**
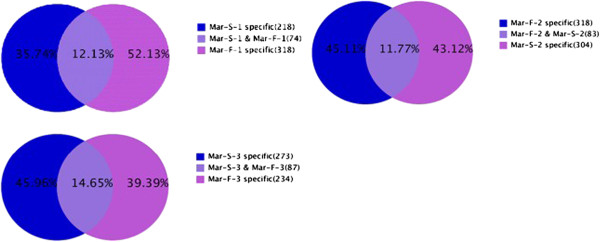
**Distribution of unique miRNAs among the six small RNA libraries.** Mar-F-1 and Mar-S-1: meiosis stage anthers from WT and GMS mutant; Mar-F-2 and Mar-S-2: tetrad stage anthers from WT and GMS mutant; Mar-F-3 and Mar-S-3: uninucleate microspore stage anthers from WT and GMS mutant.

Analyzing miRNA variations in the three anther developmental stages between the WT and its GMS mutant, we found that the Mar-S-1 and the Mar-F-1 libraries comprised 35.74% and 52.13% of the unique miRNAs, respectively; the Mar-S-2 and the Mar-F-2 libraries comprised 45.11% and 43.12%, respectively; and the Mar-S-3 and the Mar-F-3 libraries comprised 45.96% and 39.39%, respectively. Only 12.13%, 11.77% and 14.65% of the unique miRNAs were shared between the WT and its GMS mutant during the same three anther developmental stages, respectively (Figure 3). Therefore, most of the unique miRNAs found in the GMS mutant anthers were different from those in the WT anthers at the corresponding stage.

The above results indicated that various small RNA regulations were already present during the anther development of the ‘Dong A’ GMS mutant, as compared to its fertile wild type. These different small RNA varieties and diverse small RNA regulations may target different genes that influence the anther development and therefore male sterility.

### Identification of conserved cotton miRNAs

Aligning the small RNA sequences to known cotton miRNAs resulted in 405,829 and 192,554 matches for the Mar-F-1 and Mar-S-1 libraries, respectively. In the Mar-F-2 and Mar-S-2 libraries, there were 496,607 and 402,146 matches for the WT and the mutant, respectively. In the Mar-F-3 and Mar-S-3 libraries, there were 1,108,399 and 767,638 matches for the WT and the mutant, respectively (Additional file 2).

**Figure 4:**
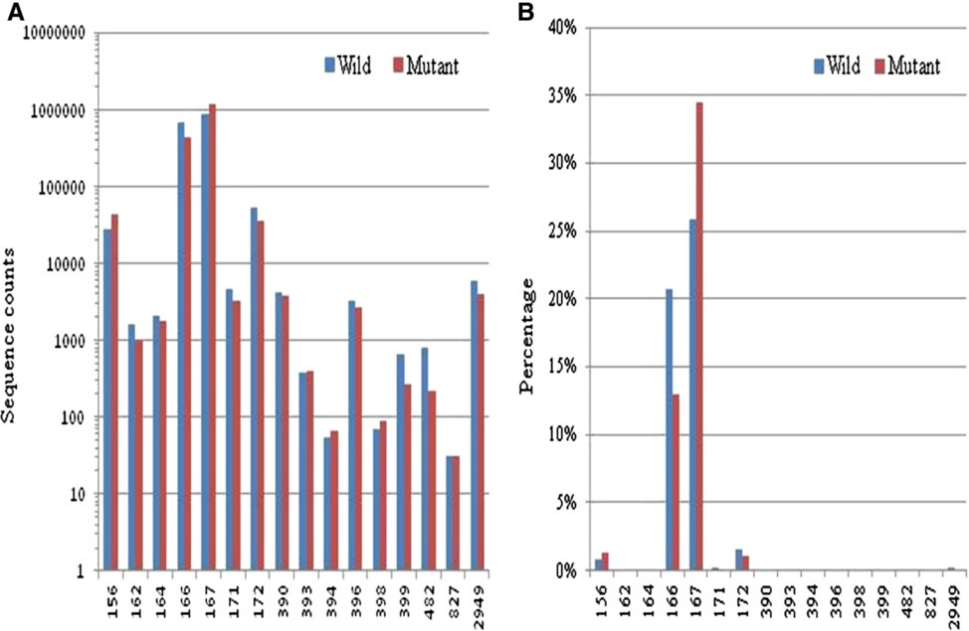
**Relative abundance and differential expression levels of the identified cotton conserved miRNA families.** (**A**) The sequence counts reflect the relative abundance of each miRNA family between the WT and GMS mutant. (**B**) The differential miRNA expression levels are presented as percentages of the total sequence count (WT + Mutant) for each family.

Sixteen conserved cotton miRNA families comprising 3,373,236 individual candidate miRNA reads were identified in the six small RNA libraries, with the Gh-miR167 and Gh-miR166 families being the most abundant, followed by the Gh-miR172 and Gh-miR156 families (Figure 4A). Of all the conserved cotton miRNA reads, Gh-miR167 dominated the WT and the mutant libraries, accounting for 25.8% (in the three WT libraries) and 34.5% (in the three GMS mutant libraries), respectively (Figure 4B). Next is Gh-miR166, which accounted for 20.7% and 12.9% in the WT and mutant libraries, respectively. In contrast, some other miRNA families showed very low expression abundance in the anthers, with very lower read counts. The varied abundance of these miRNA families suggests that the miRNA genes are differentially transcribed during anther development.

Analyzing miRNA expression between WT and its GMS mutant anthers revealed that Gh-miR394, Gh-miR396, Gh-miR398, Gh-miR399, and Gh-miR482 were differentially expressed during the meiosis stage, three of which (i.e., Gh-miR394, Gh-miR398, and Gh-miR399) were also differentially expressed during the tetrad stage and two of which (i.e., Gh-miR398 and Gh-miR482) together with Gh-miR827 were differentially expressed during the uninucleate microspore stage (Additional file 3). Thus, Gh-miR398 was in common in all the three stages and Gh-miR394, Gh-miR399, and Gh-miR482 were each differentially expressed between the ‘Dong A’ WT and the GMS mutant during two anther developmental stages.

### Degradome library construction and validation of conserved miRNA targets

In cotton, conserved miRNA targets were previously identified mainly via bioinformatics prediction [27] and only a few conserved miRNA targets have been experimentally validated [28]. In this study, in order to identify miRNA targets, a degradome library derived from anthers of the WT and GMS mutant representing three stages of development was constructed and sequenced, resulting in the generation of 24.6 million raw reads. After removal of low quality sequences and adapter sequences, 24.4 million clean reads were obtained and 98% were 20 or 21 nt in length as expected (Additional file 4) in that normally length distribution peak of degradome fragment is between 20 and 21 nt [29]. Of unique signatures, 9.5 million distinct reads of 20 and 21 nt in length were obtained and 5.68 million (59.8%) signatures (referred as mapped reads) were perfectly mapped to reference sequences in the cotton transcript assemblies database (DFCI-Cotton Gene Index, release 11.0), which represented 81.3% (95,966) of the annotated unique cotton sequences. These data indicate that the degradome library was of high quality with good genome coverage in identifying degraded mRNA targets that should contain the sequence profile resulting from miRNA directed cleavage.

By sequence alignments, a total of 896 distinct transcripts targeted by 145 unique miRNAs were detected in our degradome library (Additional file 5). Gene ontology (GO) categories based on biological processes revealed that these miRNA-target genes were related to 32 biological processes (as shown in Additional file 6); the five most frequent terms are regulation of cellular process, metabolic process, response to stimulus, macromolecule metabolic process, and primary metabolic process, indicating the importance of these miRNAs in gene regulations during cotton anther development.

**Figure 5:**
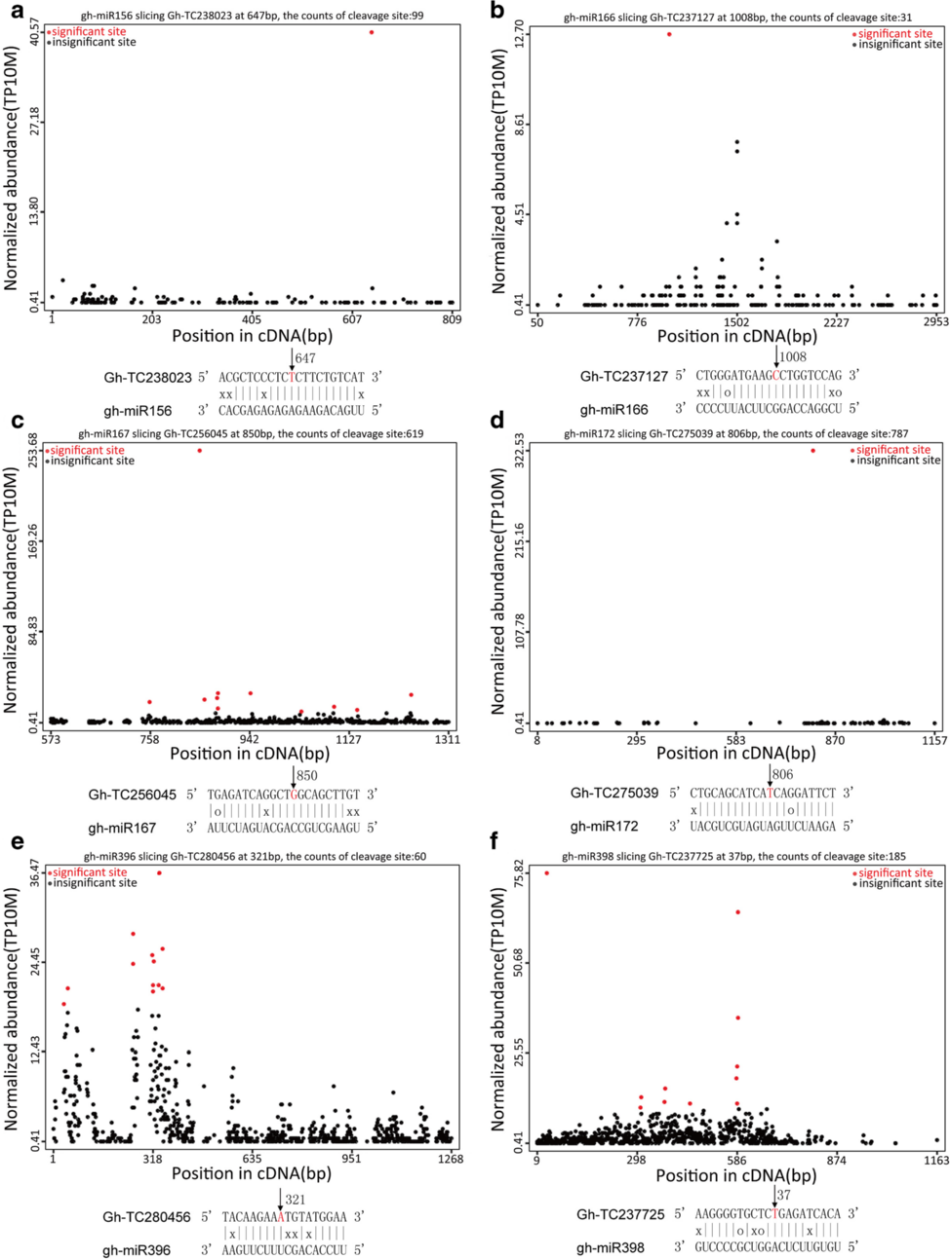
**Target plots (t-plots) of identified cotton conserved miRNA targets using degradome sequencing.** The abundance of each signature is plotted as a function of its position in the transcript. The red colored italicized nucleotide on the target transcript from the 3^′^ end indicates the cleavage site detected in the degradome library. The number next to the arrow in the alignment between the miRNA and the target is the cDNA position corresponds to the detected cleavage site. The X-axis of each t-plot represents the cDNA position range with the sequenced tags coverage. TP10M is normalized abundance in the formula TP10M = raw abundance/(total genome match – (t/r/sn/snoRNA))*10,000,000.

As shown above, many targets of conserved miRNAs were captured by the degradome analysis, which provided experimental evidence to support previous predictions. The results of degradome analysis revealed that Gh-miR156, Gh-miR166, Gh-miR167, Gh-miR172, Gh-miR396, and Gh-miR398 directed cleavages of SBP-box (TC238023, Figure 5a), class III HD-Zip like protein (TC237127, Figure 5b), auxin response factor 4 (TC256045, Figure 5c), AP2 (TC275039, Figure 5d), ACC oxidase 3 (TC280456, Figure 5e), and Cu/Zn superoxide dismutase (TC237725, Figure 5f) genes, respectively, which are key genes involved in hormone signals, cell patterning, and anti-oxidant metabolism. These identified miRNA targets using degradome sequencing are present in the form of target plots (t-plots) that plot the abundance of the signatures relative to their position in the transcripts [30]. In each of these t-plots, a clear peak for the absolute number of tags is found at the predicted cleavage site for Gh-miR156, Gh-miR166, Gh-miR167, Gh-miR172, Gh-miR396, or Gh-miR398 (Additional file 7), indicated that there are correspondences between the cleavage positions and significant sites on the t-plots.

### Validation of miRNA and target expression through TaqMan microRNA assays

To examine miRNA expression during three stages of anther development as well as validate the sequencing results, Gh-miR156a, Gh-miR166a, Gh-miR167, Gh-miR172, Gh-miR396a and Gh-miR398 were assayed to validate if these miRNAs had significant differences in expressions between the WT and GMS mutant anthers (Additional file 8). The miRNA expression patterns were similar to the sequencing results, indicating that the small RNA sequencing results were reliable.

**Figure 6:**
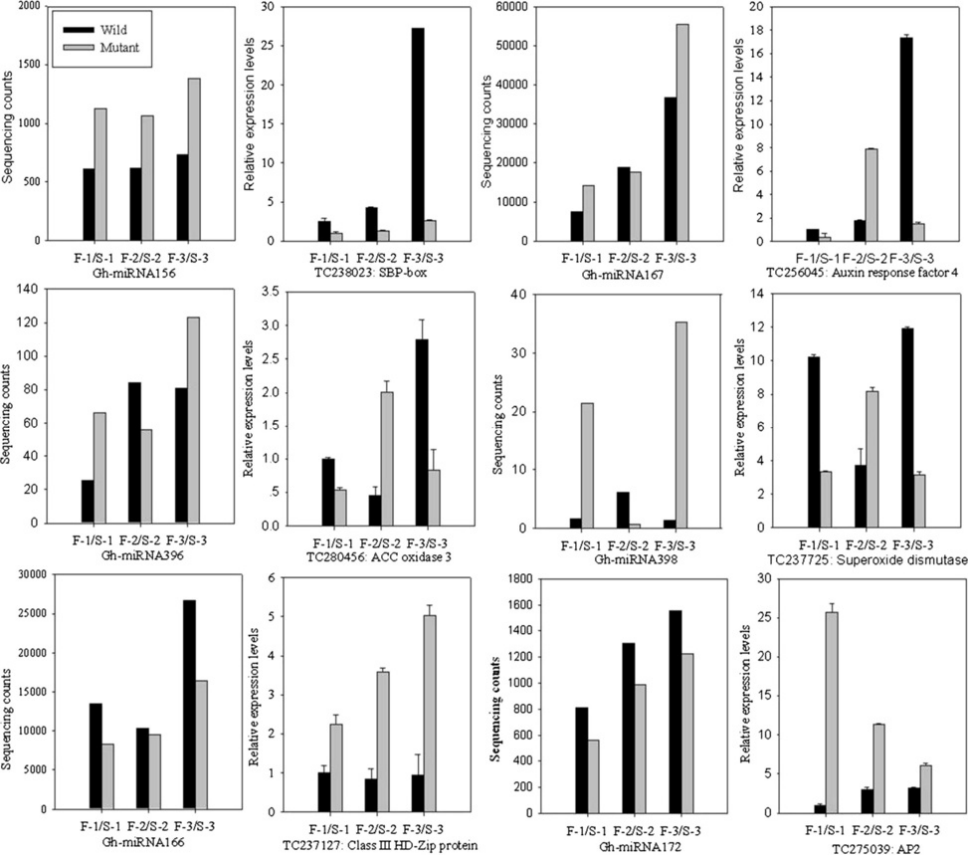
**miRNAs and their predicted target gene expressions in anthers of the WT and the GMS mutant.** F-1 and S-1: meiosis stage wild type and mutant anthers; F-2 and S-2: tetrad stage wild type and mutant anthers; F-3 and S-3: uninucleate microspore stage wild type and mutant anthers. Relative expression levels were calculated using 18S RNA as a control.

To test if any correlation between miRNAs and their targets existed, the expression patterns of identified miRNA targets based on quantitative RT-PCR (qRT-PCR) were compared (Figure 6). If a miRNA degraded its target mRNA transcripts, their expression levels could be negatively correlated. As expected, the expression levels of most miRNA were inversely correlated with these of the corresponding mRNAs. During the three anther developmental stages, Gh-miR156 expressed at a relatively higher level in the GMS mutant than in the WT, while its target gene encoding a SBP-box (TC238023) expressed in the reverse way, as expected (Figure 6). Unexpectedly, as compared with the GMS mutant, this target gene expressed at a proportionally higher level at the uninucleate stage of the WT anthers, during which stage the Gh-miR156 level was relatively lower (Figure 6). The relationship between Gh-miR167 and its target (TC256045) encoding an auxin response factor 4(ARF4)and between Gh-miR398 and its target (TC237725) encoding Cu/Zn superoxide dismutase followed a similar trend in the first and third stages (Figure 6). As compared with the WT, the GMS mutant anthers had higher expression levels of the two miRNAs and lower expression of their target genes at the meiosis and uninucleate stages. On the contrary, the GMS mutant anthers at the tetrad stage had similar (in Gh-miR167) or lower (in Gh-miR396 and Gh-miR398) expression levels of the miRNAs, but their target genes had significantly higher expression levels, as compared with the WT (Figure 6). Similarly to Gh-miR156, Gh-miR167 was up-regulated in the uninucleate microspore stage of the GMS mutant anthers as compared with the WT, while its target gene (TC256045) encoding for ARF4 expressed at a much lower level (Figure 6).

A reverse trend was noted between Gh-miR166 and its target gene coding for class III HD-Zip like protein (TC237127) and between Gh-miR172 and its target gene coding for AP2 (TC275039) in the GMS mutant as compared to the WT. The expression levels of Gh-miR166 and Gh-miR172 in the GMS mutant were significantly lower than in the WT during the three stages of the anther development, while the reverse was true for their target genes (Figure 6). It should be pointed out that, the negative correlation in expression levels between miRNAs and their target genes existed except for Gh-miR166, but the linear correlation coefficients (r= 0.64 to 0.98) were not statistically significant due in part to only three anther developmental stages sampled. The non-linear relationship of expression levels between miRNAs and their target genes may also indicate that there are other mechanisms regulating expression of the target genes.

### Analysis of novel miRNA candidates

Given the fact that the sequencing of the Upland cotton genome is incomplete, and information on genomewide cotton small RNA population is unknown, accurate identification of non-conserved miRNA in cotton is a difficult task. Following a BLASTn search and hairpin structure prediction (See Materials and Methods), 110 putative unique *G*. *hirsutum* miRNAs were detected in the six small RNA libraries (Additional file 9), including 33 in the Mar-F-1 library, 19 in the Mar-F-2 library, 45 in the Mar-F-3 library, 6 in the Mar-S-1 library, 5 in the Mar-S-2 library, and 2 in the Mar-S-3 library (Table 2). All of these newly identified miRNAs met the criteria for miRNA annotation [31].

**Table 2:** Novel miRNAs identified from the six small RNA libraries

**Name**	**Count**	**miRNA Sequence**	**Fold energy**
Mar-F-1-m0001	42	CGCUAUCCAUCCUGAGUUUCA	−50.60
Mar-F-1-m0002	8	UCUUGUACUGCAUCAUAACUU	−55.90
Mar-F-1-m0004	58	AGAGAUUGCAUUUCCUCUUCCA	−29.40
Mar-F-1-m0006	12	UAACUGAAGAGUUUGAUCAUGG	−90.50
Mar-F-1-m0011	24	UGCAAAUCCAGUCAAAAGUUA	−33.90
Mar-F-1-m0013	21	GGGAAUUUCUGAUUGUCGGGG	−46.30
Mar-F-1-m0017	34	UGCUCACUUCUCUUCUGUCAGC	−57.60
Mar-F-1-m0018	78	UUCCAUCUCUUGCACACUGGA	−44.60
Mar-F-1-m0019	13	CCAAGAGGAUUGAAGGCCAUG	−39.30
Mar-F-1-m0022	11	GAAGCGCCUGGCAAGUUAGAC	−42.80
Mar-F-1-m0024	72	CGAGCCGAAUCAAUAUCACUC	−40.10
Mar-F-1-m0025	30	AGCUGCUUGGCUAUGGAUCCC	−46.10
Mar-F-1-m0026	9	AUGACCAUUCAAGAAAGUGCU	−59.25
Mar-F-1-m0027	82	GUAGUUGAACGACGUUUAUCUA	−35.40
Mar-F-1-m0029	121	GGAGCAUCAUCAAGAUUCACA	−48.11
Mar-F-1-m0030	1765	UUACUUUAGAUGUCUCCUUCA	−48.92
Mar-F-1-m0031	52	UCCAAAGGGAUCGCAUUGAUC	−58.70
Mar-F-1-m0032	14	ACGUUAUGGGCAUGGUAUGGA	−50.92
Mar-F-1-m0033	12	CAUGACUUUUAGCGGCGUUUG	−32.80
Mar-F-1-m0035	6	UGGUUUUCAAGUGGGAUUUGCUG	−60.90
Mar-F-1-m0038	136	UUCAGAAACCAUCCCUUCCUU	−58.60
Mar-F-1-m0039	3138	ACAGCUUUAGAAAUCAUCCCU	−52.50
Mar-F-1-m0040	12	GCUCUCUAUGCUUCUGUCAUC	−55.00
Mar-F-1-m0041	9	AUAUGUUAGAUCAAAGAGUAA	−49.50
Mar-F-1-m0042	162	GGCUGUGGUUGAUUCGGCAAGA	−37.55
Mar-F-1-m0043	73	AAUGGAGGAGUUGGAAAGAUU	−37.39
Mar-F-1-m0044	14	AGUGGAUUGGGCUACAGUUUCUU	−27.10
Mar-F-1-m0048	6	AUAAAAUACUGAUGUGACAUA	−33.90
Mar-F-1-m0051	9	ACGGUUUUAAGUUUUAACUGA	−28.42
Mar-F-1-m0052	9	GACGGUUUUAAGUUUUAACUG	−28.42
Mar-F-1-m0053	12	UUGGCGAACAAAUCAGUAGGAGU	−20.80
Mar-F-1-m0054	8	ACGACAGAAAAAAGGAUUGAUCA	−30.30
Mar-F-1-m0055	21	AAGUGGGAUGGGUGGAAAGAUU	−40.90
Mar-F-2-m0006	12	GGAGGAUCUCCAGGACUUGGCUU	−36.69
Mar-F-2-m0014	219	GAUUUGGGGCAAAGACGGGAU	−42.80
Mar-F-2-m0016	7	CUGGAUGCAGAGGUUUAUCGA	−51.70
Mar-F-2-m0019	11	GUGAUUGGGCUAGGGUCUAGGCA	−28.84
Mar-F-2-m0020	9	AAAACUGGACUGUUGUAUUGGUU	−39.70
Mar-F-2-m0022	8	UUAGAUUCAUUGGCUGAGUUA	−95.50
Mar-F-2-m0034	65	AACCAAUGACUAUUCAUGAUUCC	−25.30
Mar-F-2-m0035	7	GGGAGAAAUUAGAUUGCCGA	−18.33
Mar-F-2-m0039	10	AAGCUUGCUAGGCUCAAAGCCCA	−20.00
Mar-F-2-m0043	11	GUUCGAUUCUCGGAAUGCCC	−56.80
Mar-F-2-m0046	14	GGAAGGGAUAUAACUCAGCGGUA	−28.60
Mar-F-2-m0051	12	AAAGGGAUGAUUUCUAAAGCU	−47.85
Mar-F-2-m0055	10	UAUGUUAGAUCAAAGAGUAAAUU	−48.90
Mar-F-2-m0058	18	UGCCUGGCUCCCUGUAUGCCU	−42.10
Mar-F-2-m0064	8	AUACGACUAGCGCGACUCGA	−83.97
Mar-F-2-m0065	10	AAGAGUCAGAUUGCAUUUUGC	−25.40
Mar-F-2-m0067	11	AGGUACAGAGUCUGUUGGCAU	−47.80
Mar-F-2-m0069	294	GAUGGGUGAGGGGGUAAGACA	−52.80
Mar-F-2-m0071	8	AAGAGAGAAAGAGAGGCCUGGA	−30.62
Mar-F-3-m0001	16	ACUAAAAAAUGGGCAAAUUAG	−70.85
Mar-F-3-m0002	12	CUUUGGAGGGGAGAUUAGAGC	−61.05
Mar-F-3-m0006	8	AGGGAAGGUUAGAUAUUUAUA	−77.80
Mar-F-3-m0008	46	UAGAGAUUGCAUUUCCUCUUCC	−29.40
Mar-F-3-m0014	10	CGUGGUGAUCAGUUGGACCUUU	−23.40
Mar-F-3-m0018	6	GGCAGCGGUUCAUCGAUCUCU	−28.70
Mar-F-3-m0029	5	UUUAAUUUCCUCCAAUAUCUUA	−46.64
Mar-F-3-m0031	23	UGCCUGGCUCCCUGAAUGCCA	−53.30
Mar-F-3-m0032	103	UAGCCAAGGAUGACUUGCCUG	−54.30
Mar-F-3-m0035	18	UGAUAUUGGCCUGGUUCACUC	−44.99
Mar-F-3-m0036	10	CUCUAUGGUAGAAUCAGUCGGGG	−42.60
Mar-F-3-m0038	458	UUCCACAGCUUUCUUGAACUU	−62.70
Mar-F-3-m0041	909	GGAAUGUUGUCUGGCUCGAGG	−50.90
Mar-F-3-m0043	35	AGAUCAUGUGGCAGUUUCACC	−45.64
Mar-F-3-m0044	7	AGAGCUUUCUUCAGUCCACUC	−82.60
Mar-F-3-m0048	111	UUGGUGCGGUUCAAUCAGAUA	−50.80
Mar-F-3-m0049	20	CGAAUGAUCUCGGACCAGGCU	−35.76
Mar-F-3-m0050	154	AUCAUGUGGCAGUUUCACCUG	−44.00
Mar-F-3-m0056	4369	UCUUGACCUUGUAAGACCUUU	−48.30
Mar-F-3-m0058	10	GGAAUGUUGGCUGGCUCGAAG	−52.60
Mar-F-3-m0064	38	UGAAUGAUUUCGGACCAGGCU	−40.80
Mar-F-3-m0065	28	UGGUGCAGGUCGGGAACUGAU	−76.37
Mar-F-3-m0067	65	UUCCACGGCUUUCUUGAACUU	−51.20
Mar-F-3-m0070	22	UGCAUUCUGAUGUAUGGGGAC	−69.07
Mar-F-3-m0080	14	UUUAAUAUUGUUUGGAUAUUGU	−32.70
Mar-F-3-m0087	5	CGGCAAGUUGUCUUUGGCUAC	−52.00
Mar-F-3-m0091	12	CAGGUGUAGCAUCAUCAAGAU	−63.81
Mar-F-3-m0092	30	UCAGGUCAUCUUGCAGCUUCA	−111.30
Mar-F-3-m0093	53	CGCUAUCUAUCCUGAGUUUCA	−61.50
Mar-F-3-m0095	26	UUGAAGACCCAUUUGCAACCAA	−24.80
Mar-F-3-m0110	15	CUCUUGUUGGGCAAAUGAGCAU	−22.10
Mar-F-3-m0113	6	UGGGAACUUGAAGAUGAGGCU	−29.40
Mar-F-3-m0115	15	AGGAGGAGCAGGAAGCAGUAACU	−56.90
Mar-F-3-m0120	6	UUUCAACAUAGUAGAGGGACU	−102.07
Mar-F-3-m0124	11	UAUAUGGCUUAAAACAGGCUCC	−78.30
Mar-F-3-m0127	21	UCUUUCCUACUCCUCCCAUUCC	−55.10
Mar-F-3-m0141	10	AUGGACAUCCAAGGGGGAGUGUU	−50.46
Mar-F-3-m0146	15	UCCCUUUGGAUGUCUUCUUGC	−75.70
Mar-F-3-m0164	22	UGGCUUCUAGACAGUGGAUGCA	−23.40
Mar-F-3-m0178	24	UGUUGGCUCGGUUCACUCAGA	−61.50
Mar-F-3-m0183	21	AUGCACUGCCUCUUCCCUGGC	−50.60
Mar-F-3-m0185	160	UGGAGGCAGCGGUUCAUCGAUC	−36.30
Mar-F-3-m0198	7	AAGAGUCAGAUUGCAUUUUG	−25.40
Mar-F-3-m0200	9	AGAGGUGAUCAUGGGCCGGG	−21.20
Mar-F-3-m0205	32	CAGCCCUGGUGUUGGACAUUC	−46.00
Mar-S-1-m0013	9	GGGGCUGCAUUGAAGUGAAGGCU	−76.70
Mar-S-1-m0014	7	UUCCACAGCUUUCUUGAACUG	−49.30
Mar-S-1-m0035	12	ACAGGACAGGACAGGACAGGACA	−68.99
Mar-S-1-m0039	13	UGGCGUAUGAGGAGCCAUGCA	−51.90
Mar-S-1-m0097	18	AUAUGGCUUAAAACAGGCUCCA	−78.30
Mar-S-1-m0110	8	AGAGGAGAAGAAAAUUCACUAUA	−45.65
Mar-S-2-m0027	7	AUUGGUGAUUGACAUUUUUAUCU	−33.10
Mar-S-2-m0040	67	GGAAUGGAGGAGUUGGAAAGA	−47.59
Mar-S-2-m0043	6	UCUUUGAUCUAAUAUACAGG	−40.30
Mar-S-2-m0046	7	AGUGUAAGACCUGUCUGGGACA	−28.50
Mar-S-2-m0051	6	CUGAGGUUGGGUCGGACGACA	−30.02
Mar-S-3-m0014	6	UCCAUGGUGGAGAUUGCUCUU	−30.00
Mar-S-3-m0041	16	UGUUGAAGGUCGAUGGGUUAA	−39.70

Comparing the expression of these novel miRNAs between WT and GMS mutant anthers, 43, 22 and 56 novel miRNAs were significantly differentially expressed in the meiosis, the tetrad and the uninucleate microspore stages, respectively (Additional file 10). Identification of target genes for these novel miRNAs suggests that they may participate in various aspects of anther development. For example, novel miRNA Mar-F-1-m0031 was identified to target gene encoding a transport inhibitor response 1 (TIR1, a receptor of IAA, Additional file 11), which can directly bind to auxin through the formation of the SCFTIR1 complex and is the key protein in the Aux/IAA degradation pathway of the 26S proteasome [32].

## Discussion

Small RNAs regulate many aspects of anther development. However, no existing studies have reported the relationship between miRNAs and male sterility in cotton. To understand the underlying molecular basis resulting in the male sterility of the cotton GMS mutant, six small RNA libraries were constructed during the anther development of ‘Dong A’ WT and its GMS mutant in the current work. To the best of our knowledge, the present study represents one of the first such attempts.

Millions of unique small RNA sequences of 18–30 nt in length were detected, including 110 novel miRNAs, thus enriching the number of known unique small RNAs in cotton. Sixteen conserved miRNA families were detected in this study. Many canonical miRNAs are conserved among mosses, eudicots, and monocots, and some have conserved functions among land plants [33]. For example, the mature canonical Gh-miR167 in cotton is identical to those in poplar and Arabidopsis. These conserved miRNAs may play an important role in cotton anther development, as many of their targets mediate biological pathways, such as auxin responses and cell patterning, as implicated in the regulation of anther development, based on previous studies [34].

Both Gh-miR167 and Gh-miR166 were predominantly expressed during anther development in ‘Dong A’ WT and its GMS mutant (Figure 4), an indication of important roles in regulating cotton anther development. In this study, Gh-miR167 and Gh-miR166 were identified to target ARF4 and class III HD-Zip like protein, respectively. As compared with the wild type, Gh-miR167 was expressed at a relatively higher level in the uninucleate microspore stage, which led to down-regulation of ARF4 by ten-fold in the GMS mutant anthers (Figure 6). The much lower expression level of ARF4 may affect the auxin response pathway in the GMS mutant, which was consistent with the lower content of IAA in the uninucleate microspore stage of the GMS mutant anthers (Additional file 12). In Arabidopsis, miR166 is thought to target mRNAs that encode a class III HD-Zip-like protein that plays a critical role in shoot apical meristem initiation and leaf polarity and pattern formation [35,36]. However, the relationship between male sterility and the lower level of Gh-miR166 in the GMS mutant anthers relative to WT anthers is currently unknown and needs further studies.

miR156 and miR172 target SQUAMOSA PROMOTER BINDING PROTEIN transcription factors (SBP-box) and APETALA2 (AP2), respectively, which have been predicted to play important roles in anther development [37,38]. miR156 directly represses the expression of SBP-box transcription factors that play an important role in juvenile-to-adult transition throughout the plant kingdom [39]. It has been shown that miR156 directly promotes the transcription of miR172 via SBP-box, and miR172 acts downstream of miR156 to promote adult epidermal identity [40]. Furthermore, the miR156-regulated SBP-box is a direct upstream activator of LEAFY, FRUITFULL, and APETALA1 [41]. In this study, Gh-miR156 and Gh-miR172 were moderately expressed in ‘Dong A’ WT and its GMS mutant (Figure 4). Compared to these of the WT, the anthers from the three anther developmental stages of the GMS mutant had higher expression levels of Gh-miR156 and lower expression of its target SBP-box. In contrast to the fact that over-expression of miR172 resulted in male sterility in Arabidopsis and rice [20,38], we detected lower level of expression of Gh-miR172 and higher level of expression of its target AP2 in the GMS mutant anthers at the three anther developmental stages (Figure 6). Therefore, the relationship between miR156/miR172 and male sterility in the GMS mutant is likely different and needs further studies.

Gh-miR396 was identified to target ACC oxidase 3 (TC280456), a key branch-point enzyme involved in ethylene biosynthetic process [42]. In anther development, ethylene is important for male gametophyte germination and anther dehiscence [43,44] and it has been reported that fertile male gametophyte development is accompanied by two peaks of ethylene production in anther tissues and the mature pollen is characterized by a high content of ethylene [45]. In the current study, Gh-miR396 was differentially expressed between ‘Dong A’ WT and its GMS mutant anthers in the meiosis stage, and it had a higher level of expression in the GMS mutant anthers in the uninucleate microspore stage. This is consistent with the relatively lower level of expression of its target gene ACC oxidase 3 (Figure 6). However, whether the opposite expressions of Gh-miR396 and its target gene in the GMS mutant anthers leading to a significant reduction in ethylene synthesis remains to be studied.

Gh-miR398 targets mRNA (TC237725) that encodes Cu/Zn superoxide dismutase (Cu/Zn SOD), which plays an important role in plant antioxidant metabolism [46]. In plants, the prominent role of reactive oxygen species (ROS) has been revealed in induction, signaling, and execution of programmed cell death (PCD) [47]. ROS can trigger release of cytochrome *c*, which is a ROS-derived PCD feature shared among mammalian, plant and yeast mitochondria [48]. Previous studies revealed that excessive accumulation of O_2_^-2^ and H_2_O_2_, and a significant reduction in ROS-scavenging enzyme activity coincide with male cell death in cytoplasmic male sterile of cotton [49]. Budar and Pelletier reasoned that the difference in SOD gene expression between the cotton male sterile line and its maintainer may result in an imbalance in ROS metabolism and male sterility [50]. In the present study, we showed the existence of different underlying miRNA pathways that may regulate enzymatic activities in the WT and its GMS mutant. Surprisingly, Gh-miRNA398 was up-regulated by twenty-fold and its target gene Cu/Zn SOD was reversely much lower expressed in the uninucleate microspore stage of GMS mutant anthers, as compared to the WT anthers (Figure 6). Up-regulation of cytochrome *c* by threefold was observed in the corresponding stage of the GMS mutant anthers (Additional file 13). The decreased Cu/Zn SOD activity and elevated expression level of cytochrome *c* in the GMS mutant anthers may lead to a transient oxidative burst and significant ROS accumulation. However, more studies are needed to understand the underlying mechanisms that lead to male sterility in the GMS mutant.

## Conclusion

Using a deep sequencing strategy, a number of miRNAs expressed during three anther development stages of cotton were identified. The differential expression of the miRNAs between the GMS mutant and its WT indicates that miRNAs are distinctly involved in cotton anther development and male sterility. Further studies of these differentially expressed miRNAs and their targets in the anthers will provide a better understanding of the regulatory mechanisms underlying male sterility in cotton.

## Methods

### Plant materials and anther collection

Upland cotton (*G*. *hirsutum*) ‘Dong A’ (WT) plants and the GMS mutant in the ‘Dong A’ background were grown under regular field conditions at the experimental farm of the Cotton Research Institute in China Agricultural Academy of Science. Previous study revealed that when the longitudinal length of buds reach 5.0mm, 6.5mm, and 9mm, respectively, the pollen mother cell of the GMS mutant enter the meiosis, tetrad and uninucleate stages [51]. According to this sampling criterion and combined with microscopic examination, developing anthers at these three different growth stages were collected during early mornings. The excised anthers were frozen in liquid nitrogen and stored at −80°C for analysis.

### Small RNA sequencing and library construction

Total RNA was extracted from anthers using the pBiozol Total RNA Extraction Reagent (BioFlux), in accordance with the manufacturer’s instructions. The RNA was then precipitated with ethanol, dissolved in diethypyrocarbonate (DEPC) water and stored at −80°C. All RNA samples were examined for protein contamination (A_260_/A_280_ ratios) and reagent contamination (A_260_/A_230_ ratios) using a Nanodrop ND 1000 spectrophotometer (NanoDrop, Wilmington, DE).

The samples from the WT and GMS mutant anthers were quantified and equalized so that equivalent amounts of RNA were analyzed. The extracted total RNA was resolved on denatured 15% polyacrylamide gels. Gel fragments with a size range of 18–30 nt were excised, and the small RNA fragments were eluted overnight with 0.5 M NaCl at 4°C, and precipitated with ethanol. These 18–30 nt small RNAs were given 5^′^ and 3^′^ RNA adapters that were ligated with T4 RNA ligase. The adapter-ligated small RNAs were subsequently transcribed into cDNA by Super-Script II Reverse Transcriptase (Invitrogen) and amplified with the polymerase chain reaction, using primers that were annealed to the ends of the adapters. The amplified cDNA products were purified and recovered. Finally, Solexa sequencing technology was employed to sequence the small RNA samples (BGI, Shenzhen, China).

### Analysis of sequencing data

Raw sequence reads were produced using an Illumina 1G Genome Analyzer at BGI (Shenzhen, China) and processed into clean full-length reads through the BGI small RNA pipelines. During this procedure, all low quality reads were removed, such as reads with 3^′^ and 5^′^ adapter contaminants, those without insert tags, and those with poly A sequences. The remaining high-quality sequences were trimmed of their adapter sequences, and those larger than 30 nt or smaller than 18 nt were discarded. All high-quality sequences, even those with only a single unique read, were considered significant and used for further analysis and the sequences were deposited in NCBI with an accession number of GSE43531.

A chi-square test was performed to determine the statistical significance of the differences between the WT and GMS mutant small RNA libraries following a previously described method [52].

### Identification of novel miRNAs

To identify potentially novel miRNAs among the six small RNA libraries, cotton transcript assemblies (http://occams.dfci.harvard.edu/pub/bio/tgi/data/Gossypium ) from the Dana Farber Cancer Institute were chosen to map unique small RNA sequences. The characteristic hairpin structure of miRNA precursors was used to predict possible novel miRNAs. The miRNA prediction software, mireap, was also used to predict novel miRNA based on the secondary structure, the Dicer cleavage site, and the minimum free energy (http://sourceforge.net/projects/mireap/).

### Identification of miRNAs and their targets by degradome sequencing

The small RNAs were aligned to miRNA precursors/mature miRNAs in the miRBase (http://www.mirbase.org/index.shtml, release 15.0). The following criteria were used to determine the sequence counts of miRNA families in the different tissue samples: (1) if there was cotton miRNA information in the miRBase, the small RNAs were aligned to the corresponding cotton miRNA precursor/mature miRNA; and (2) if there was no cotton miRNA information in the miRBase, the small RNAs were aligned to the miRNA precursors/mature miRNAs of all plants in the database.

Most plant miRNAs facilitate the degradation of their mRNA targets by slicing precisely between the tenth and eleventh nucleotides (nt) from the 5^′^ end of the miRNA. As a result, the 3^′^ fragment of the target mRNA possesses a monophosphate at its 5^′^ end. This important property has been used to validate miRNA targets [53]. In this study, in order to dissect miRNA-guided gene regulation in the WT and GMS mutant anthers, a degradome library suitable for miRNA target identification was constructed as described previously [29]. Briefly, total RNAs, which were extracted from the WT and GMS mutant anthers representing three stages of development, respectively, were mixed at an equal molar ratio as one sample. Approximately 200 μg of the mixed total RNA was used for polyadenylation using the Oligotex mRNA mini kit (Qiagen). Using T4 RNA ligase (Takara), a 5^′^ RNA adapter was added to the cleavage products, which possessed a free 5^′^-monophosphate at their 3^′^ termini. The ligated products were then purified using Oligotex mRNA mini kit (Qiagen) for reverse transcription to generate the first strand of cDNA using an oligo dT primer via SuperScript II RT (Invitrogen). After the cDNA library was amplified for 6 cycles (94°C for 30 s, 60°C for 20 s, and 72°C for 3 min) using Phusion Taq (NEB), the PCR products were digested with restriction enzyme *Mme* I (NEB). A double-stranded DNA adapter was then ligated to the digested products using T4 DNA ligase (NEB). The ligated products were selected based on size by running 10% polyacrylamide gel and purified for the final PCR amplification (94°C for 30 s, 60°C for 20 s, and 72°C for 20 s) for 20 cycles. The PCR products were gel purified and used for high-throughput sequencing using Illumina HiSeq 2000.

Low quality sequences and adapters were removed before sequence analysis and the clean sequences were deposited in NCBI with an accession number of GSE43389. Unique sequence signatures were aligned to the database of cotton transcript assemblies in Cotton Gene Index (Release 11.0, http://occams.dfci.harvard.edu/pub/bio/tgi/data/Gossypium ) using SOAP software (http://soap.genomics.org.cn/). The CleaveLand was used to detect potentially cleaved targets based on degradome sequences. The 20 and 21 nt distinct reads were subjected to the CleaveLand pipeline for small RNA targets identification as previously described [54]. Briefly, the 20 and 21 nt distinct reads were first normalized to give“reads per million” (RPM). Subsequently, the degradome reads were mapped to the cotton annotated cDNA (DFCI-Cotton Gene Index, release 11.0) and the cDNA hit number of each degradome read was recorded. Raw abundances in the target library was normalized according to the formula: normalized abundance (TP10M) = raw abundance/(total genome match – (t/r/sn/snoRNA))*10,000,000. All alignments with scores not exceeding 4 and having the 5^′^ end of the degradome sequence coincident with the tenth and eleventh nucleotides of complementarity to the small RNA were retained. To evaluate the potential functions of miRNA-targeted genes, gene ontology (GO) categories (http://www.geneontology.org/) were used for assignment of the identified target genes according to the previously described method [55].

### The qRT-PCR of miRNAs

qRT-PCR reactions were carried out in final volumes of 20 μl containing 10 μl 2×TaqMan Universal PCR Master Mix, 1 μl 20×TaqMan MicroRNA Assay primers and probes, 7.67 μl nuclease-free water, and 1.33 μl product from RT reactions using a ABI 7500 Real-Time PCR system (Applied Biosystems). The reactions were incubated in a 96-well plate at 95°C for 10 min, followed by 40 cycles of 95°C for 15 s and 60°C for 60 s. Cotton 18S was used to normalize the amounts of gene-specific RT-PCR products [56].

## Authors’ contributions

MMW, SXY, SLF, and JWY designed the experiments. SLF performed the field cotton plant cultivation and anther collection. MZS conceived the study, participated in its design, as well as drafted and amended the manuscript. MMW wrote the manuscript draft and ZJF edited and revised the manuscript. MMW, HLW, and WM performed the experiments. All authors read and approved the final manuscript.

## Supplementary Material

Additional file 1 The number of total miRNA and siRNA in six small RNA libraries. Click here for file

Additional file 2 Cotton conserved miRNA families in six small RNA libraries. Click here for file

Additional file 3 The number of significant differentially expressed cotton conserved miRNA in the six small RNA. Click here for file

Additional file 4 Length distribution of small RNAs in degradome library. Click here for file

Additional file 5 The number of distinct transcripts targeted by unique miRNAs detected in degradome library. Click here for file

Additional file 6 GO analysis of miRNA target genes identified in the WT and GMS mutant anthers representing three stages of development. Click here for file

Additional file 7 The significant sites on the t-plots. Click here for file

Additional file 8 **Comparison of the qRT-PCR results of the identified cotton miRNAs with the Solexa sequencing results of the corresponding miRNAs.** (a), (c), (e) Solexa sequencing results of miRNAs; (b), (d), (f) qRT-PCR results of miRNAs. F-1 and S-1: meiosis stage of the wild and mutant anthers; F-2 and S-2: tetrad stage of the wild and mutant anthers; F-3 and S-3: uninucleate microspore stage of the wild and mutant anthers. Relative expression levels (R.E.Ls) were calculated using 18S as a control. Click here for file

Additional file 9 **Novel miRNAs identified from six small RNA libraries**. Click here for file

Additional file 10 The number of significant differentially expressed novel cotton miRNA in the six libraries. Click here for file

Additional file 11 **Target plots (t-plots) of identified novel miRNA (Mar-F-1-m0031) targets using degradome sequencing.** The abundance of each signature is plotted as a function of its position in the transcript. The red colored italicized nucleotide on the target transcript from the 3^′^ end indicates the cleavage site detected in the degradome library. Click here for file

Additional file 12 **Measurment of IAA contents in the uninucleate microspore stage of WT and GMS mutant anthers using high performance liquid chromatography.** F-3 and S-3: uninucleate microspore stage wild type and mutant anthers. Click here for file

Additional file 13 **The qRT-PCR results of cytochrome *c *in anthers of the WT and GMS mutant.** Click here for file
