# Supplementary figures and images for: Comparative expression profiling of miRNA during anther development in genetic male sterile and wild type cotton
Source: BMC Plant Biol. 2013 Apr 19;13:66. doi: 10.1186/1471-2229-13-66 (PMC3639194; doi:10.1186/1471-2229-13-66)

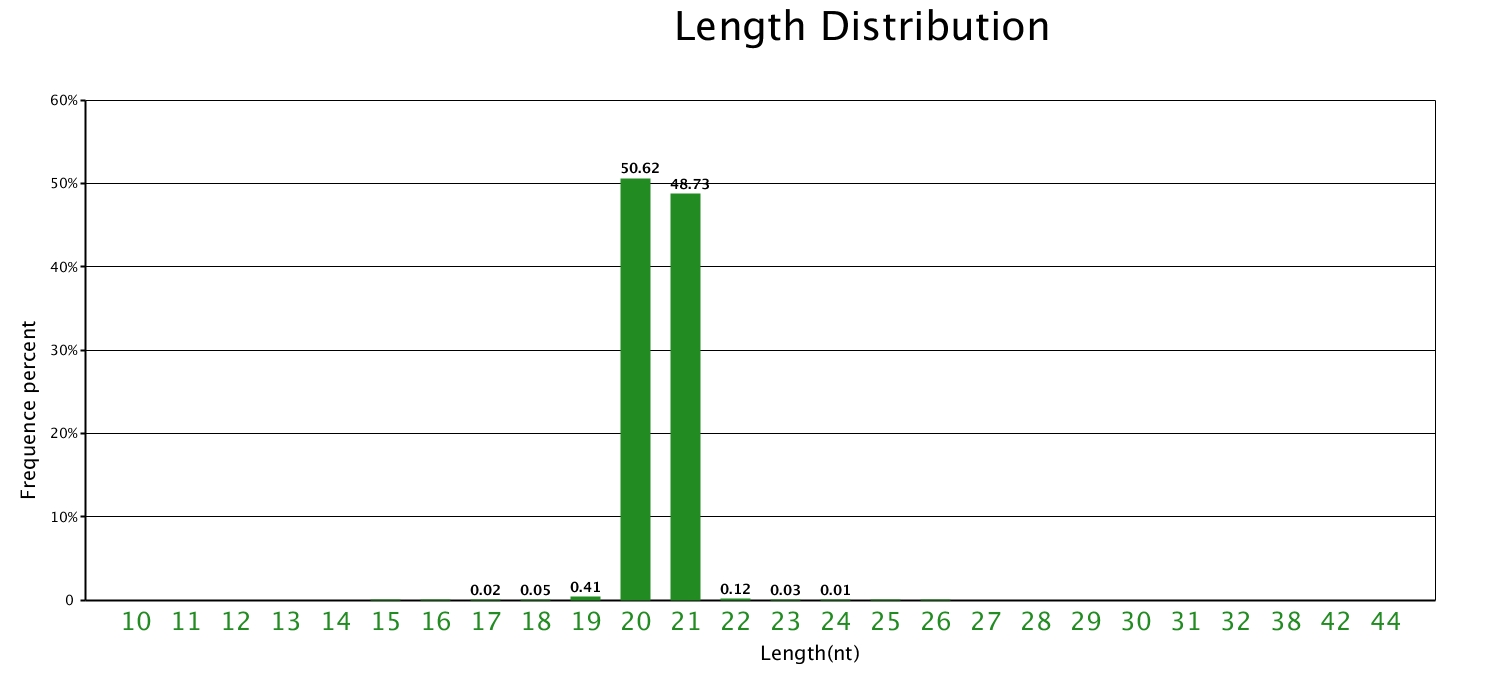

Supplement: Additional file 4 — Length distribution of small RNAs in degradome library. [file 1471-2229-13-66-S4.jpeg]

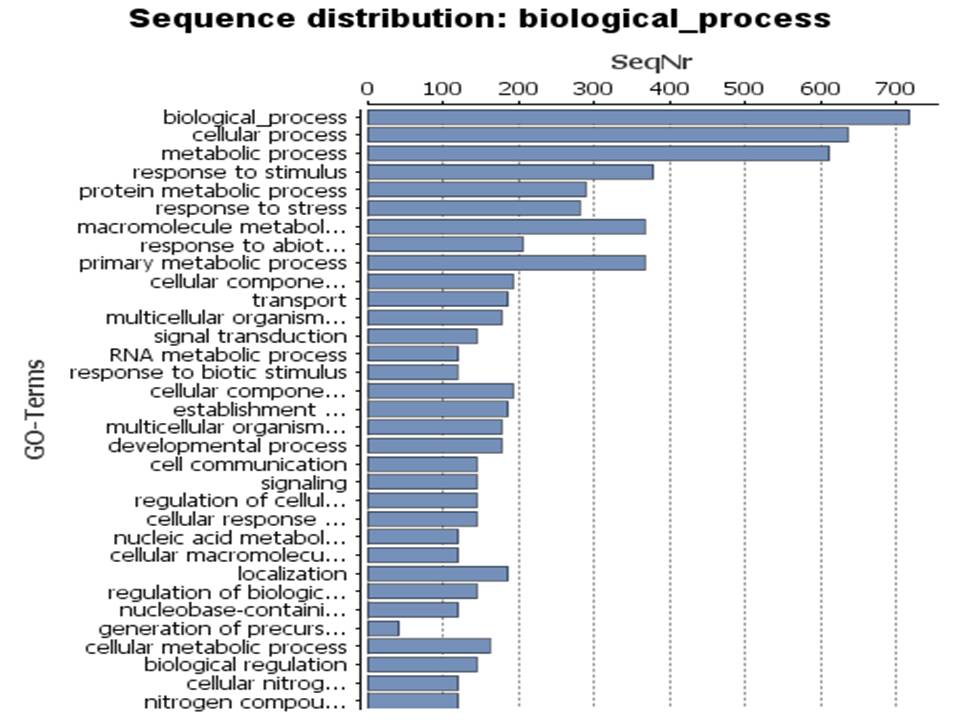

Supplement: Additional file 6 — GO analysis of miRNA target genes identified in the WT and GMS mutant anthers representing three stages of development. [file 1471-2229-13-66-S6.jpeg]

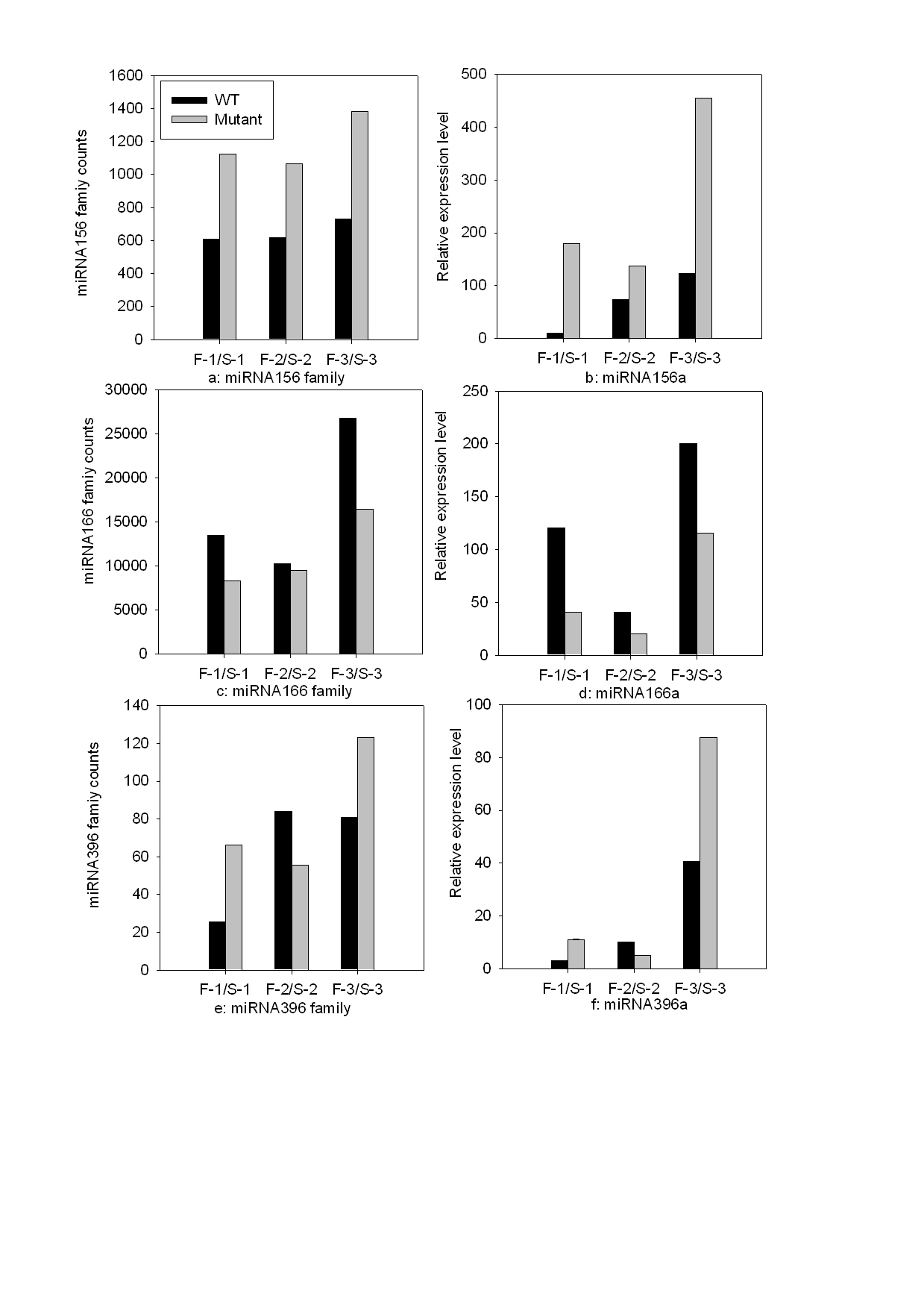

Supplement: Additional file 8 — Comparison of the qRT-PCR results of the identified cotton miRNAs with the Solexa sequencing results of the corresponding miRNAs. (a), (c), (e) Solexa sequencing results of miRNAs; (b), (d), (f) qRT-PCR results of miRNAs. F-1 and S-1: meiosis stage of the wild and mutant anthers; F-2 and S-2: tetrad stage of the wild and mutant anthers; F-3 and S-3: uninucleate microspore stage of the wild and mutant anthers. Relative expression levels (R.E.Ls) were calculated using 18S as a control. [file 1471-2229-13-66-S8.jpeg]

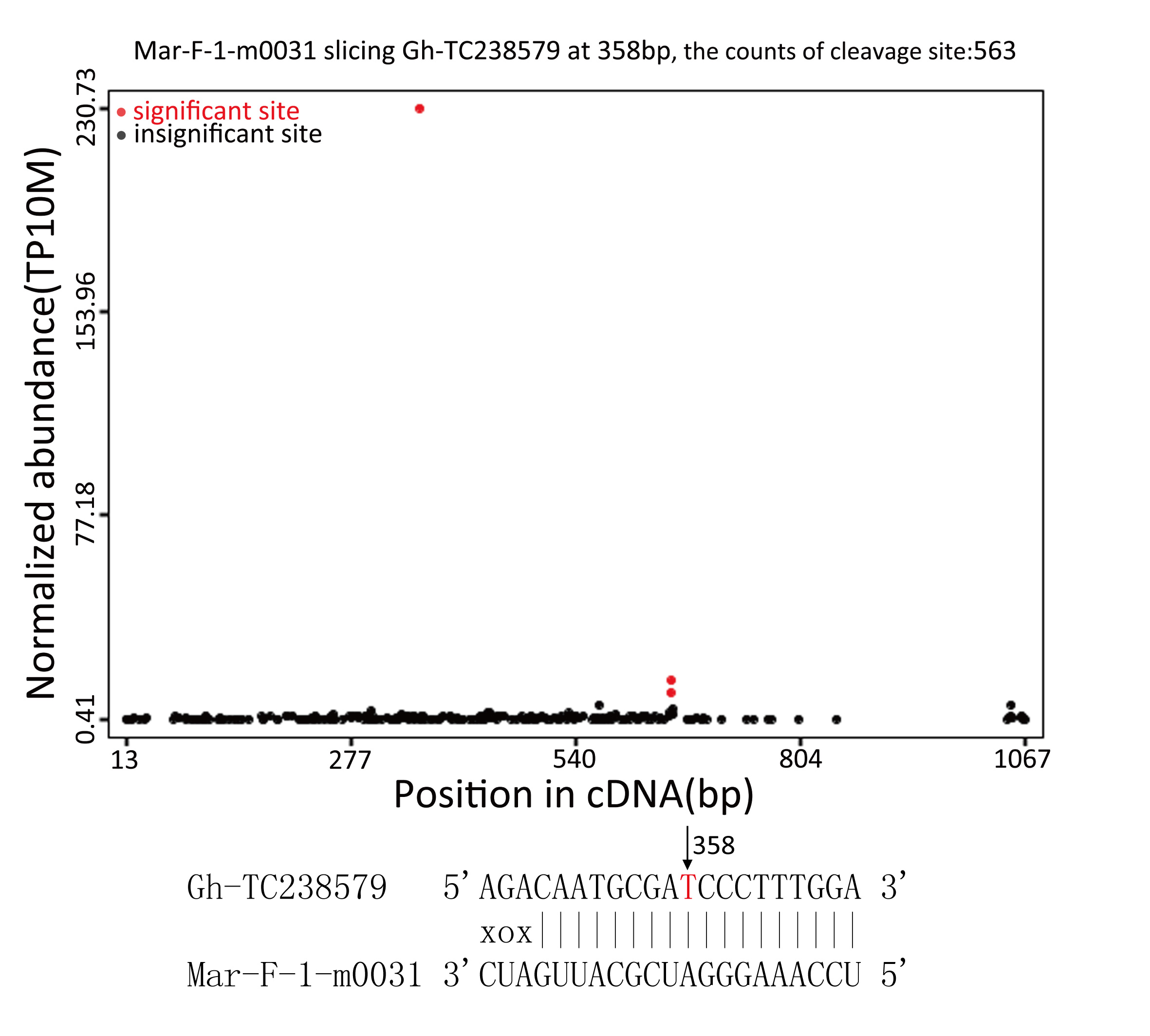

Supplement: Additional file 11 — Target plots (t-plots) of identified novel miRNA (Mar-F-1-m0031) targets using degradome sequencing. The abundance of each signature is plotted as a function of its position in the transcript. The red colored italicized nucleotide on the target transcript from the 3′ end indicates the cleavage site detected in the degradome library. [file 1471-2229-13-66-S11.jpeg]

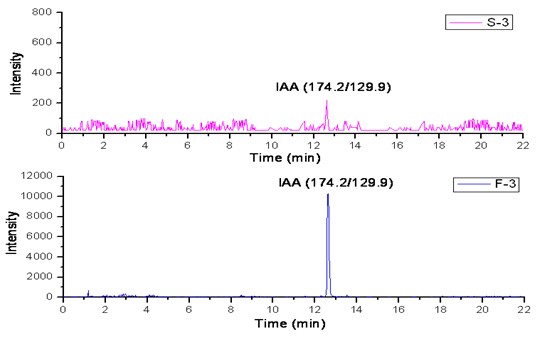

Supplement: Additional file 12 — Measurment of IAA contents in the uninucleate microspore stage of WT and GMS mutant anthers using high performance liquid chromatography. F-3 and S-3: uninucleate microspore stage wild type and mutant anthers. [file 1471-2229-13-66-S12.jpeg]
