# Supplementary material for: Comparative expression profiling of miRNA during anther development in genetic male sterile and wild type cotton
Source: BMC Plant Biol. 2013 Apr 19;13:66. doi: 10.1186/1471-2229-13-66 (PMC3639194; doi:10.1186/1471-2229-13-66)
Supplement: Additional file 9 — Novel miRNAs identified from six small RNA libraries. [file 1471-2229-13-66-S9.docx]

Supplemental file 9**.** Novel miRNAs identified from six small RNA libraries.

| Name | Count | Sequence | Fold energy |
| --- | --- | --- | --- |
| Mar-F-1-m0001 | 42 | CGCTATCCATCCTGAGTTTCA | -50.60 |
| Mar-F-1-m0002 | 8 | TCTTGTACTGCATCATAACTT | -55.90 |
| Mar-F-1-m0004 | 58 | AGAGATTGCATTTCCTCTTCCA | -29.40 |
| Mar-F-1-m0006 | 12 | TAACTGAAGAGTTTGATCATGG | -90.50 |
| Mar-F-1-m0011 | 24 | TGCAAATCCAGTCAAAAGTTA | -33.90 |
| Mar-F-1-m0013 | 21 | GGGAATTTCTGATTGTCGGGG | -46.30 |
| Mar-F-1-m0017 | 34 | TGCTCACTTCTCTTCTGTCAGC | -57.60 |
| Mar-F-1-m0018 | 78 | TTCCATCTCTTGCACACTGGA | -44.60 |
| Mar-F-1-m0019 | 13 | CCAAGAGGATTGAAGGCCATG | -39.30 |
| Mar-F-1-m0022 | 11 | GAAGCGCCTGGCAAGTTAGAC | -42.80 |
| Mar-F-1-m0024 | 72 | CGAGCCGAATCAATATCACTC | -40.10 |
| Mar-F-1-m0025 | 30 | AGCTGCTTGGCTATGGATCCC | -46.10 |
| Mar-F-1-m0026 | 9 | ATGACCATTCAAGAAAGTGCT | -59.25 |
| Mar-F-1-m0027 | 82 | GTAGTTGAACGACGTTTATCTA | -35.40 |
| Mar-F-1-m0029 | 121 | GGAGCATCATCAAGATTCACA | -48.11 |
| Mar-F-1-m0030 | 1765 | TTACTTTAGATGTCTCCTTCA | -48.92 |
| Mar-F-1-m0031 | 52 | TCCAAAGGGATCGCATTGATC | -58.70 |
| Mar-F-1-m0032 | 14 | ACGTTATGGGCATGGTATGGA | -50.92 |
| Mar-F-1-m0033 | 12 | CATGACTTTTAGCGGCGTTTG | -32.80 |
| Mar-F-1-m0035 | 6 | TGGTTTTCAAGTGGGATTTGCTG | -60.90 |
| Mar-F-1-m0038 | 136 | TTCAGAAACCATCCCTTCCTT | -58.60 |
| Mar-F-1-m0039 | 3138 | ACAGCTTTAGAAATCATCCCT | -52.50 |
| Mar-F-1-m0040 | 12 | GCTCTCTATGCTTCTGTCATC | -55.00 |
| Mar-F-1-m0041 | 9 | ATATGTTAGATCAAAGAGTAA | -49.50 |
| Mar-F-1-m0042 | 162 | GGCTGTGGTTGATTCGGCAAGA | -37.55 |
| Mar-F-1-m0043 | 73 | AATGGAGGAGTTGGAAAGATT | -37.39 |
| Mar-F-1-m0044 | 14 | AGTGGATTGGGCTACAGTTTCTT | -27.10 |
| Mar-F-1-m0048 | 6 | ATAAAATACTGATGTGACATA | -33.90 |
| Mar-F-1-m0051 | 9 | ACGGTTTTAAGTTTTAACTGA | -28.42 |
| Mar-F-1-m0052 | 9 | GACGGTTTTAAGTTTTAACTG | -28.42 |
| Mar-F-1-m0053 | 12 | TTGGCGAACAAATCAGTAGGAGT | -20.80 |
| Mar-F-1-m0054 | 8 | ACGACAGAAAAAAGGATTGATCA | -30.30 |
| Mar-F-1-m0055 | 21 | AAGTGGGATGGGTGGAAAGATT | -40.90 |
| Mar-F-2-m0006 | 12 | GGAGGATCTCCAGGACTTGGCTT | -36.69 |
| Mar-F-2-m0014 | 219 | GATTTGGGGCAAAGACGGGAT | -42.80 |
| Mar-F-2-m0016 | 7 | CTGGATGCAGAGGTTTATCGA | -51.70 |
| Mar-F-2-m0019 | 11 | GTGATTGGGCTAGGGTCTAGGCA | -28.84 |
| Mar-F-2-m0020 | 9 | AAAACTGGACTGTTGTATTGGTT | -39.70 |
| Mar-F-2-m0022 | 8 | TTAGATTCATTGGCTGAGTTA | -95.50 |
| Mar-F-2-m0034 | 65 | AACCAATGACTATTCATGATTCC | -25.30 |
| Mar-F-2-m0035 | 7 | GGGAGAAATTAGATTGCCGA | -18.33 |
| Mar-F-2-m0039 | 10 | AAGCTTGCTAGGCTCAAAGCCCA | -20.00 |
| Mar-F-2-m0043 | 11 | GTTCGATTCTCGGAATGCCC | -56.80 |
| Mar-F-2-m0046 | 14 | GGAAGGGATATAACTCAGCGGTA | -28.60 |
| Mar-F-2-m0051 | 12 | AAAGGGATGATTTCTAAAGCT | -47.85 |
| Mar-F-2-m0055 | 10 | TATGTTAGATCAAAGAGTAAATT | -48.90 |
| Mar-F-2-m0058 | 18 | TGCCTGGCTCCCTGTATGCCT | -42.10 |
| Mar-F-2-m0064 | 8 | ATACGACTAGCGCGACTCGA | -83.97 |
| Mar-F-2-m0065 | 10 | AAGAGTCAGATTGCATTTTGC | -25.40 |
| Mar-F-2-m0067 | 11 | AGGTACAGAGTCTGTTGGCAT | -47.80 |
| Mar-F-2-m0069 | 294 | GATGGGTGAGGGGGTAAGACA | -52.80 |
| Mar-F-2-m0071 | 8 | AAGAGAGAAAGAGAGGCCTGGA | -30.62 |
| Mar-F-3-m0001 | 16 | ACTAAAAAATGGGCAAATTAG | -70.85 |
| Mar-F-3-m0002 | 12 | CTTTGGAGGGGAGATTAGAGC | -61.05 |
| Mar-F-3-m0006 | 8 | AGGGAAGGTTAGATATTTATA | -77.80 |
| Mar-F-3-m0008 | 46 | TAGAGATTGCATTTCCTCTTCC | -29.40 |
| Mar-F-3-m0014 | 10 | CGTGGTGATCAGTTGGACCTTT | -23.40 |
| Mar-F-3-m0018 | 6 | GGCAGCGGTTCATCGATCTCT | -28.70 |
| Mar-F-3-m0029 | 5 | TTTAATTTCCTCCAATATCTTA | -46.64 |
| Mar-F-3-m0031 | 23 | TGCCTGGCTCCCTGAATGCCA | -53.30 |
| Mar-F-3-m0032 | 103 | TAGCCAAGGATGACTTGCCTG | -54.30 |
| Mar-F-3-m0035 | 18 | TGATATTGGCCTGGTTCACTC | -44.99 |
| Mar-F-3-m0036 | 10 | CTCTATGGTAGAATCAGTCGGGG | -42.60 |
| Mar-F-3-m0038 | 458 | TTCCACAGCTTTCTTGAACTT | -62.70 |
| Mar-F-3-m0041 | 909 | GGAATGTTGTCTGGCTCGAGG | -50.90 |
| Mar-F-3-m0043 | 35 | AGATCATGTGGCAGTTTCACC | -45.64 |
| Mar-F-3-m0044 | 7 | AGAGCTTTCTTCAGTCCACTC | -82.60 |
| Mar-F-3-m0048 | 111 | TTGGTGCGGTTCAATCAGATA | -50.80 |
| Mar-F-3-m0049 | 20 | CGAATGATCTCGGACCAGGCT | -35.76 |
| Mar-F-3-m0050 | 154 | ATCATGTGGCAGTTTCACCTG | -44.00 |
| Mar-F-3-m0056 | 4369 | TCTTGACCTTGTAAGACCTTT | -48.30 |
| Mar-F-3-m0058 | 10 | GGAATGTTGGCTGGCTCGAAG | -52.60 |
| Mar-F-3-m0064 | 38 | TGAATGATTTCGGACCAGGCT | -40.80 |
| Mar-F-3-m0065 | 28 | TGGTGCAGGTCGGGAACTGAT | -76.37 |
| Mar-F-3-m0067 | 65 | TTCCACGGCTTTCTTGAACTT | -51.20 |
| Mar-F-3-m0070 | 22 | TGCATTCTGATGTATGGGGAC | -69.07 |
| Mar-F-3-m0080 | 14 | TTTAATATTGTTTGGATATTGT | -32.70 |
| Mar-F-3-m0087 | 5 | CGGCAAGTTGTCTTTGGCTAC | -52.00 |
| Mar-F-3-m0091 | 12 | CAGGTGTAGCATCATCAAGAT | -63.81 |
| Mar-F-3-m0092 | 30 | TCAGGTCATCTTGCAGCTTCA | -111.30 |
| Mar-F-3-m0093 | 53 | CGCTATCTATCCTGAGTTTCA | -61.50 |
| Mar-F-3-m0095 | 26 | TTGAAGACCCATTTGCAACCAA | -24.80 |
| Mar-F-3-m0110 | 15 | CTCTTGTTGGGCAAATGAGCAT | -22.10 |
| Mar-F-3-m0113 | 6 | TGGGAACTTGAAGATGAGGCT | -29.40 |
| Mar-F-3-m0115 | 15 | AGGAGGAGCAGGAAGCAGTAACT | -56.90 |
| Mar-F-3-m0120 | 6 | TTTCAACATAGTAGAGGGACT | -102.07 |
| Mar-F-3-m0124 | 11 | TATATGGCTTAAAACAGGCTCC | -78.30 |
| Mar-F-3-m0127 | 21 | TCTTTCCTACTCCTCCCATTCC | -55.10 |
| Mar-F-3-m0141 | 10 | ATGGACATCCAAGGGGGAGTGTT | -50.46 |
| Mar-F-3-m0146 | 15 | TCCCTTTGGATGTCTTCTTGC | -75.70 |
| Mar-F-3-m0164 | 22 | TGGCTTCTAGACAGTGGATGCA | -23.40 |
| Mar-F-3-m0178 | 24 | TGTTGGCTCGGTTCACTCAGA | -61.50 |
| Mar-F-3-m0183 | 21 | ATGCACTGCCTCTTCCCTGGC | -50.60 |
| Mar-F-3-m0185 | 160 | TGGAGGCAGCGGTTCATCGATC | -36.30 |
| Mar-F-3-m0198 | 7 | AAGAGTCAGATTGCATTTTG | -25.40 |
| Mar-F-3-m0200 | 9 | AGAGGTGATCATGGGCCGGG | -21.20 |
| Mar-F-3-m0205 | 32 | CAGCCCTGGTGTTGGACATTC | -46.00 |
| Mar-S-1-m0013 | 9 | GGGGCTGCATTGAAGTGAAGGCT | -76.70 |
| Mar-S-1-m0014 | 7 | TTCCACAGCTTTCTTGAACTG | -49.30 |
| Mar-S-1-m0035 | 12 | ACAGGACAGGACAGGACAGGACA | -68.99 |
| Mar-S-1-m0039 | 13 | TGGCGTATGAGGAGCCATGCA | -51.90 |
| Mar-S-1-m0097 | 18 | ATATGGCTTAAAACAGGCTCCA | -78.30 |
| Mar-S-1-m0110 | 8 | AGAGGAGAAGAAAATTCACTATA | -45.65 |
| Mar-S-2-m0027 | 7 | ATTGGTGATTGACATTTTTATCT | -33.10 |
| Mar-S-2-m0040 | 67 | GGAATGGAGGAGTTGGAAAGA | -47.59 |
| Mar-S-2-m0043 | 6 | TCTTTGATCTAATATACAGG | -40.30 |
| Mar-S-2-m0046 | 7 | AGTGTAAGACCTGTCTGGGACA | -28.50 |
| Mar-S-2-m0051 | 6 | CTGAGGTTGGGTCGGACGACA | -30.02 |
| Mar-S-3-m0014 | 6 | TCCATGGTGGAGATTGCTCTT | -30.00 |
| Mar-S-3-m0041 | 16 | TGTTGAAGGTCGATGGGTTAA | -39.70 |

mireap-plant

Mar-F-1-m0001 gi|109839591|gb|DW238152.1|DW238152:58:178:+ 121(nt) -50.60(kcal/mol)

GGAAGAATCTGTTAAGCTCAGGAGGGATAGCGCCATGGATGATCCATCTTTCAAGCTTTCATGCATGTATGTTATTTGATCCTCATATAGCGCTATCCATCCTGAGTTTCATCGGTTCTTC Mar-F-1-m0001 42

.((((((((.((.((((((((((.(((((((((.(((((.((((...(...((.((......)).))...)......)))).)).))).))))))))).)))))))))).)).))))))))

******************************************************************************************CGCTATCCATCCTGAGTTTCA********** Mar-F-1-m0001-3p 42

------------------------------------------------------------------------------------------CGCTATCCATCCTGAGTTTC----------- t0214217 5

------------------------------------------------------------------------------------------CGCTATCCATCCTGAGTTTCA---------- t0013337 37

//

Mar-F-1-m0002 gi|109845403|gb|DW243961.1|DW243961:210:356:- 147(nt) -55.90(kcal/mol)

TGGTAGCTTACGACTAGTTATGATGCATTATAAGATGTAATGACCATTACCCAATGTGGACTGATCAGTAGCTTTTAAAAAGATACGATGCATGATAAGATGTAATGCTAATTACATCTTGTACTGCATCATAACTTGTAAGTTACC Mar-F-1-m0002 9

.(((((((((((...((((((((((((.(((((((((((((...((((((((.....)).((.(((((((.(((.....)))......))).)))).))..))))))...))))))))))))).)))))))))))))))))))))))

********************************************************************************************************************TCTTGTACTGCATCATAACTT********** Mar-F-1-m0002-3p 8

-------------------------------------------CCATTACCCAATGTGGACT------------------------------------------------------------------------------------- t2340459 1

--------------------------------------------------------------------------------------------------------------------TCTTGTACTGCATCATAACT----------- t0999940 2

--------------------------------------------------------------------------------------------------------------------TCTTGTACTGCATCATAACTT---------- t0163059 6

//

Mar-F-1-m0004 gi|109849019|gb|DW477998.1|DW477998:429:550:+ 122(nt) -29.40(kcal/mol)

AGTCTGTTATCGCAAGAGGAATCTGCAATATTCTTTGAGCAATGTCCATTTGGAAGGTTCAATCCCTGGTATCCATCGATCAATCCAAATAGAGATTGCATTTCCTCTTCCAACTCTCCAGC Mar-F-1-m0004 58

...........(.((((((((..((((((.((((((((((....(((....)))..))))))....(((.(((....)))....)))...)))))))))).)))))))).)...........

******************************************************************************************AGAGATTGCATTTCCTCTTCCA********** Mar-F-1-m0004-3p 58

-----------------------------------------------------------------------------------------TAGAGATTGCATTTCCTCT-------------- t0425871 3

-----------------------------------------------------------------------------------------TAGAGATTGCATTTCCTCTT------------- t0400902 3

-----------------------------------------------------------------------------------------TAGAGATTGCATTTCCTCTTC------------ t0061734 12

-----------------------------------------------------------------------------------------TAGAGATTGCATTTCCTCTTCC----------- t0079173 10

-----------------------------------------------------------------------------------------TAGAGATTGCATTTCCTCTTCCA---------- t0288024 4

------------------------------------------------------------------------------------------AGAGATTGCATTTCCTCT-------------- t4082762 1

------------------------------------------------------------------------------------------AGAGATTGCATTTCCTCTT------------- t6317226 1

------------------------------------------------------------------------------------------AGAGATTGCATTTCCTCTTCC----------- t0267616 4

------------------------------------------------------------------------------------------AGAGATTGCATTTCCTCTTCCA---------- t0034761 19

-------------------------------------------------------------------------------------------GAGATTGCATTTCCTCTTCCA---------- t7428962 1

//

Mar-F-1-m0006 gi|109849221|gb|DW478200.1|DW478200:466:715:- 250(nt) -90.50(kcal/mol)

ATAACTGAAGAGTTTGATCATGGCTCAGATTGAACGCTGGCGGCAGGCCTAACACATGCAAGTCGAGCGGATGAGAGGAGCTTGCTCCTCGATTCAGCGGCGGACGGGTGAGTAATGCCTAGGAATCTGCCTAGTAGTGGGGGACAACGTTTCGAAAGGAACGCTAATACCGCATACGTCCTACGGGAGAAAGTGGGGGATCTTCGGACCTCACGCTATTAGATGAGCCTAGGTCGGATTAGCTAGTTGG Mar-F-1-m0006 13

.((((((...(((((((((..(((((.((((...(((((.((((..((.........))..))))...((((..((((((....)))))).))))..)))))...((((.......))))...))))...(((((((((((((....((((((....))))))......(((......((((((........))))))......))).)))).)))))))))..)))))..)))))))))...)))))).

*TAACTGAAGAGTTTGATCATGG*********************************************************************************************************************************************************************************************************************************** Mar-F-1-m0006-5p 12

-TAACTGAAGAGTTTGATCAT------------------------------------------------------------------------------------------------------------------------------------------------------------------------------------------------------------------------------------- t4775366 1

-TAACTGAAGAGTTTGATCATG------------------------------------------------------------------------------------------------------------------------------------------------------------------------------------------------------------------------------------ t0875137 2

-TAACTGAAGAGTTTGATCATGG----------------------------------------------------------------------------------------------------------------------------------------------------------------------------------------------------------------------------------- t0322536 4

-TAACTGAAGAGTTTGATCATGGCT--------------------------------------------------------------------------------------------------------------------------------------------------------------------------------------------------------------------------------- t0827336 2

--AACTGAAGAGTTTGATCATGG----------------------------------------------------------------------------------------------------------------------------------------------------------------------------------------------------------------------------------- t6398879 1

---ACTGAAGAGTTTGATCATGGCT--------------------------------------------------------------------------------------------------------------------------------------------------------------------------------------------------------------------------------- t6972565 1

----CTGAAGAGTTTGATCATGGCTCAG------------------------------------------------------------------------------------------------------------------------------------------------------------------------------------------------------------------------------ t6846040 1

------------TTTGATCATGGCTCAGATT--------------------------------------------------------------------------------------------------------------------------------------------------------------------------------------------------------------------------- t4959576 1

//

Mar-F-1-m0011 gi|109864195|gb|DW493171.1|DW493171:229:334:+ 106(nt) -33.90(kcal/mol)

TTAGTTTGCGTCTTGTCTTTTGAACTGGATTTGCCGACTTGAACTGCGTTGTTGAAGGTTTACAAAACCCGAGCCTGCAAATCCAGTCAAAAGTTAAACCGGGCTG Mar-F-1-m0011 24

.(((((((.((.(((.(((((((.((((((((((.(.(((((((......)))...((((.....)))))))).).))))))))))))))))).))))))))))))

***************************************************************************TGCAAATCCAGTCAAAAGTTA********** Mar-F-1-m0011-3p 24

-------------------------------------------------------------------------CCTGCAAATCCAGTCAAAAGT------------ t5825814 1

---------------------------------------------------------------------------TGCAAATCCAGTCAAAAGTTA---------- t0029468 21

----------------------------------------------------------------------------GCAAATCCAGTCAAAAGTTA---------- t0876542 2

//

Mar-F-1-m0013 gi|109865386|gb|DW494362.1|DW494362:170:311:+ 142(nt) -46.30(kcal/mol)

TCTGGGAACACATGCCGGCGATCGATCCCAGTCTCATACGAGATCGTATGCTGGCTATACAGAATAAAATCCAAGCCCTCGAAAACCGAAAGAGGGCCCTCATCCTAGAACGGGAATTTCTGATTGTCGGGGCGGCCTCCAG Mar-F-1-m0013 21

.(((((..(.(.(.(((((((((((((((.(((.((((((....))))))..)))...................((((((...........))))))..............))))...)).))))))))).).))..)))))

***************************************************************************************************************GGGAATTTCTGATTGTCGGGG********** Mar-F-1-m0013-3p 21

---------------------------------------------------------------------------------------------------------------GGGAATTTCTGATTGTCGGG----------- t0657632 2

---------------------------------------------------------------------------------------------------------------GGGAATTTCTGATTGTCGGGG---------- t0156143 6

---------------------------------------------------------------------------------------------------------------GGGAATTTCTGATTGTCGGGGC--------- t2045045 1

---------------------------------------------------------------------------------------------------------------GGGAATTTCTGATTGTCGGGGCGG------- t0263688 4

-----------------------------------------------------------------------------------------------------------------GAATTTCTGATTGTCGGGG---------- t1532143 1

-----------------------------------------------------------------------------------------------------------------GAATTTCTGATTGTCGGGGCG-------- t2696344 1

-----------------------------------------------------------------------------------------------------------------GAATTTCTGATTGTCGGGGCGGC------ t2575005 1

-----------------------------------------------------------------------------------------------------------------GAATTTCTGATTGTCGGGGCGGCC----- t5963704 1

------------------------------------------------------------------------------------------------------------------AATTTCTGATTGTCGGGGCGGCC----- t5833289 1

------------------------------------------------------------------------------------------------------------------AATTTCTGATTGTCGGGGCGGCCT---- t0562582 3

//

Mar-F-1-m0017 gi|109879497|gb|DW508467.1|DW508467:227:334:- 108(nt) -57.60(kcal/mol)

GGAAGGAGAGGTTTGACAGAAGAGAGTGAGCACACGCAGGCAGATTGTATGAAAGGCCATACCTTTGCCCGCCGTGTGCTCACTTCTCTTCTGTCAGCTTAACAATTC Mar-F-1-m0017 34

.(((....(((((.(((((((((((((((((((((((.((((((..(((((......))))).)))))).).))))))))))).)))))))))))))))).....)))

****************************************************************************TGCTCACTTCTCTTCTGTCAGC********** Mar-F-1-m0017-3p 34

--------------------------------------------------------------------------TGTGCTCACTTCTCTTCTGTC------------- t5101098 1

----------------------------------------------------------------------------TGCTCACTTCTCTTCTGTC------------- t4219039 1

----------------------------------------------------------------------------TGCTCACTTCTCTTCTGTCAGC---------- t0017186 31

-----------------------------------------------------------------------------GCTCACTTCTCTTCTGTCAGC---------- t5275249 1

//

Mar-F-1-m0018 gi|109885784|gb|DW514754.1|DW514754:97:189:+ 93(nt) -44.60(kcal/mol)

TTATGTTATATTCCATCTCTTGCACACTGGACTAGCCAGCTTTTTGTTGGCTTCAGCTTCAGGTTGGTGTGCAGGGGGTGGAATACATCATTG Mar-F-1-m0018 78

..(((...(((((((((((((((((((..(.(((((((((.....)))))))........)).)..)))))))))))))))))))...)))..

**********TTCCATCTCTTGCACACTGGA************************************************************** Mar-F-1-m0018-5p 78

----------TTCCATCTCTTGCACACT----------------------------------------------------------------- t0338827 4

----------TTCCATCTCTTGCACACTG---------------------------------------------------------------- t0172284 6

----------TTCCATCTCTTGCACACTGGA-------------------------------------------------------------- t0005820 68

//

Mar-F-1-m0019 gi|109888102|gb|DW517071.1|DW517071:448:600:+ 153(nt) -39.30(kcal/mol)

AAAGAAGAAGCCAAGAGGATTGAAGGCCATGGGAAGTGTGGAAAGTGATTCTGAAAGAGATTGATTGATTGATACAAATGAATCCACACAGGTGGTCTGCCTTAGCCTGGGGAGACGATTACTGATATAGCTTTTCTTTTCTGTTTTTTCATT Mar-F-1-m0019 13

...((((((((..((((((..((((((.(((....(((((((.(((.(.(((.....))).).))).(((......)))...))))))).(((.((((.(((......))).)))).))).....))).)))))).))))))))))))))...

**********CCAAGAGGATTGAAGGCCATG************************************************************************************************************************** Mar-F-1-m0019-5p 13

----------CCAAGAGGATTGAAGGCCA---------------------------------------------------------------------------------------------------------------------------- t8109032 1

----------CCAAGAGGATTGAAGGCCATG-------------------------------------------------------------------------------------------------------------------------- t0134486 7

----------CCAAGAGGATTGAAGGCCATGG------------------------------------------------------------------------------------------------------------------------- t3989124 1

-----------CAAGAGGATTGAAGGCCATG-------------------------------------------------------------------------------------------------------------------------- t1200670 2

------------AAGAGGATTGAAGGCCATG-------------------------------------------------------------------------------------------------------------------------- t7695700 1

------------AAGAGGATTGAAGGCCATGG------------------------------------------------------------------------------------------------------------------------- t6788935 1

//

Mar-F-1-m0022 gi|164240685|gb|ES801346.1|ES801346:12:196:+ 185(nt) -42.80(kcal/mol)

AAGCATTAGAGAAGCGCCTGGCAAGTTAGACAAACTTCGAAGTCAATCCTTATCAATCTCAATCTACTTTAGATATTTGCTGGAATTTGTTCTCTAATTCAAATCCAAGCAGCAGCATTATTACTATTATTTTTATTGCCTTCAATTTCTTTCTCTAACTTGTCTTCGCCCTTCTCTCATTCTTA Mar-F-1-m0022 11

(((.((.(((((((.((..(((((((((((.(((....((((.((((..............(((((...)))))..((((((((.((((..........))))))).)))))......................)))).))))......))).)))))))))))...)).))))))).)).))).

**********GAAGCGCCTGGCAAGTTAGAC********************************************************************************************************************************************************** Mar-F-1-m0022-5p 11

-------AGAGAAGCGCCTGGCAAGTTAG------------------------------------------------------------------------------------------------------------------------------------------------------------ t1746710 1

----------GAAGCGCCTGGCAAGTTA------------------------------------------------------------------------------------------------------------------------------------------------------------- t6519575 1

----------GAAGCGCCTGGCAAGTTAG------------------------------------------------------------------------------------------------------------------------------------------------------------ t1115963 2

----------GAAGCGCCTGGCAAGTTAGA----------------------------------------------------------------------------------------------------------------------------------------------------------- t4190631 1

----------GAAGCGCCTGGCAAGTTAGAC---------------------------------------------------------------------------------------------------------------------------------------------------------- t0158837 6

//

Mar-F-1-m0024 gi|164297310|gb|ES809290.1|ES809290:29:118:+ 90(nt) -40.10(kcal/mol)

GGAGTAGACACGACGTGATATTGGTTCGGCTCATCTTATTGTGATTAAAAATTTCAAGGCGAGCCGAATCAATATCACTCATGTATGCTT Mar-F-1-m0024 72

.(((((.(((.((.((((((((((((((((((..(((...((........))...)))..)))))))))))))))))))).))).)))))

***********************************************************CGAGCCGAATCAATATCACTC********** Mar-F-1-m0024-3p 72

-----------------------------------------------------------CGAGCCGAATCAATATCAC------------ t0994301 2

-----------------------------------------------------------CGAGCCGAATCAATATCACT----------- t1001245 2

-----------------------------------------------------------CGAGCCGAATCAATATCACTC---------- t0005743 68

//

Mar-F-1-m0025 gi|164305589|gb|ES824206.1|ES824206:590:686:+ 97(nt) -46.10(kcal/mol)

GGGTATATTGAGCTGCTTGGCTATGGATCCCACAGTCCTATCCCATGGTTGGAGGGATAGGCTTGCGGGCTCCATATCTCAGGAGCTTCATTACCTT Mar-F-1-m0025 30

(((((...((((((.((.((.((((((.(((.(((.((((((((.........)))))))).))).))).)))))).)).)).))).))).))))).

**********AGCTGCTTGGCTATGGATCCC****************************************************************** Mar-F-1-m0025-5p 30

----------AGCTGCTTGGCTATGGATCC------------------------------------------------------------------- t0906649 2

----------AGCTGCTTGGCTATGGATCCC------------------------------------------------------------------ t0019542 28

//

mireap-plant

Mar-F-1-m0026 gi|164306863|gb|ES841525.1|ES841525:640:893:+ 254(nt) -59.25(kcal/mol)

AAAGTTAAAAATGACCATTCAAGAAAGTGCTCTAAAATTATATTCAACATTATGTGAAGTAGAAAGTTTAACTGATGATGAGCTCTATCACGCATTGATCAAAATTTTAGATTATCCAACGCAAATGCTTATTTTCTTTAGTTTACTGTACAAATTGGAATAGGTCAAAAGATTTCTTGCTGACCATTAAATATCATGGGTTCTGTTGATGATATTTTGGGTACTTTTTTGTGTTTGGTAATATTTGGGATGGT Mar-F-1-m0026 9

...(((..(((((((((..(((((((((((.(((((((.....((((((...(((((.(((...(((..((((((.(((((((............(((((.........)))))............)))))))....)))))).))).)))...........(((((.(((....)))..)))))........))))).....))))))....))))))))))))))))))....))))..)))))..)))...

**********ATGACCATTCAAGAAAGTGCT******************************************************************************************************************************************************************************************************************************* Mar-F-1-m0026-5p 9

---------AATGACCATTCAAGAAAG----------------------------------------------------------------------------------------------------------------------------------------------------------------------------------------------------------------------------------- t0351955 3

---------AATGACCATTCAAGAAAGTGCTCT----------------------------------------------------------------------------------------------------------------------------------------------------------------------------------------------------------------------------- t8071166 1

----------ATGACCATTCAAGAAAGT---------------------------------------------------------------------------------------------------------------------------------------------------------------------------------------------------------------------------------- t3399279 1

----------ATGACCATTCAAGAAAGTGCT------------------------------------------------------------------------------------------------------------------------------------------------------------------------------------------------------------------------------- t0296143 4

//

mireap-plant

Mar-F-1-m0027 gi|164318687|gb|ES792617.1|ES792617:632:787:- 156(nt) -35.40(kcal/mol)

AATGGCTTCCTAAACAGCTTGTGTAACTGCCATATATCCGCCTTCTATAGTAGACACAGGTACTGTAGATTGTAAAGTAGACTCCCAACTCATAGGGGCTTTAGCAAGAGTAAACAGATACCCGGTAGTTGAACGACGTTTATCTAGATCACCAGC Mar-F-1-m0027 83

..(((..((.(((((.(.((((.((((((((...((((.((..(((((((((........)))))))))..))...((..((((((........)).((....))..))))..)).))))...)))))))).))))))))))....))...)))..

****************************************************************************************************************************GTAGTTGAACGACGTTTATCTA********** Mar-F-1-m0027-3p 82

----------------------------------------------------------------------------------------------GGGGCTTTAGCAAGAGTAAACAGA-------------------------------------- t4615634 1

----------------------------------------------------------------------------------------------------------------------------GTAGTTGAACGACGTTTAT------------- t2716467 1

----------------------------------------------------------------------------------------------------------------------------GTAGTTGAACGACGTTTATC------------ t2757343 1

----------------------------------------------------------------------------------------------------------------------------GTAGTTGAACGACGTTTATCTA---------- t0011089 43

-----------------------------------------------------------------------------------------------------------------------------TAGTTGAACGACGTTTATCTA---------- t0014227 36

------------------------------------------------------------------------------------------------------------------------------AGTTGAACGACGTTTATCTA---------- t1691712 1

//

Mar-F-1-m0029 gi|164352093|gb|ES839084.1|ES839084:189:299:+ 111(nt) -48.11(kcal/mol)

GTTTGCTGTTGGAGCATCATCAAGATTCACAATTTTAGGGTTCTTTTTTGATTGATTTGATCAATCAGCCTTTTTATGATTGAGAATCCTGATGATGCTGCAGTGGCAATA Mar-F-1-m0029 121

..((((..(((.((((((((((.(((((.(((((..(((((.......((((((((...))))))))))))).....))))).))))).)))))))))).)))..))))..

**********GGAGCATCATCAAGATTCACA******************************************************************************** Mar-F-1-m0029-5p 121

---------TGGAGCATCATCAAGATTCACA-------------------------------------------------------------------------------- t5630228 1

----------GGAGCATCATCAAGATTC----------------------------------------------------------------------------------- t5818345 1

----------GGAGCATCATCAAGATTCA---------------------------------------------------------------------------------- t6841174 1

----------GGAGCATCATCAAGATTCAC--------------------------------------------------------------------------------- t0047031 15

----------GGAGCATCATCAAGATTCACA-------------------------------------------------------------------------------- t0003105 102

-----------GAGCATCATCAAGATTCACAA------------------------------------------------------------------------------- t7509759 1

//

Mar-F-1-m0031 gi|164360788|gb|ES827656.1|ES827656:74:213:+ 140(nt) -58.70(kcal/mol)

GGTGGAGAGCTCCAAAGGGATCGCATTGATCTAATGTTGATGATGGGCTTCCTTGAAAACCTTAACGAATTAATTATTGATTTTTGTTGATTCAGTTCAGTACCCATTGGATCATGCGATCCCTTCGGAATTTTCCATCG Mar-F-1-m0031 52

(((((((((.(((.((((((((((((.(((((((((....(((.((..(((...)))..)).....((((((((...........))))))))...))).....))))))))))))))))))))).))).))))))))).

**********TCCAAAGGGATCGCATTGATC************************************************************************************************************* Mar-F-1-m0031-5p 52

----------TCCAAAGGGATCGCATTG---------------------------------------------------------------------------------------------------------------- t0293376 4

----------TCCAAAGGGATCGCATTGA--------------------------------------------------------------------------------------------------------------- t0537431 3

----------TCCAAAGGGATCGCATTGAT-------------------------------------------------------------------------------------------------------------- t0070087 11

----------TCCAAAGGGATCGCATTGATC------------------------------------------------------------------------------------------------------------- t0062644 12

----------TCCAAAGGGATCGCATTGATCT------------------------------------------------------------------------------------------------------------ t0089431 9

----------TCCAAAGGGATCGCATTGATCTAAT--------------------------------------------------------------------------------------------------------- t1529043 1

------------CAAAGGGATCGCATTGAT-------------------------------------------------------------------------------------------------------------- t7113814 1

------------CAAAGGGATCGCATTGATCTAA---------------------------------------------------------------------------------------------------------- t0084757 10

-------------AAAGGGATCGCATTGATCTAAT--------------------------------------------------------------------------------------------------------- t2690480 1

//

mireap-plant

Mar-F-1-m0032 gi|21092412|gb|BQ404725.1|BQ404725:41:142:+ 102(nt) -50.92(kcal/mol)

AAGCGAAGTTACGTTATGGGCATGGTATGGATTGCTGTTTTTGATGATTTGTTGCTTTCATTAACAGCAAATTATACCGTGCCCATGACTGTAGCATCACTC Mar-F-1-m0032 14

.((.((.(((((((((((((((((((((((.((((((((..(((.............)))..)))))))).)))))))))))))))))).))))).)).)).

**********ACGTTATGGGCATGGTATGGA*********************************************************************** Mar-F-1-m0032-5p 14

----------ACGTTATGGGCATGGTATG------------------------------------------------------------------------- t0544762 3

----------ACGTTATGGGCATGGTATGG------------------------------------------------------------------------ t0787259 2

----------ACGTTATGGGCATGGTATGGA----------------------------------------------------------------------- t0092182 9

//

mireap-plant

Mar-F-1-m0033 gi|21093413|gb|BQ405726.1|BQ405726:351:417:+ 67(nt) -32.80(kcal/mol)

ATTTTTTTATCATGACTTTTAGCGGCGTTTGCAAGAAAAGCGTCGCTAAAGGTCATGATCTTTAGAC Mar-F-1-m0033 12

........((((((((((((((((((((((.......))))))))))))))))))))))........

**********CATGACTTTTAGCGGCGTTTG************************************ Mar-F-1-m0033-5p 12

----------CATGACTTTTAGCGGCGTTT------------------------------------- t2912243 1

----------CATGACTTTTAGCGGCGTTTG------------------------------------ t0081818 10

-----------ATGACTTTTAGCGGCGTTTG------------------------------------ t6265076 1

//

Mar-F-1-m0035 gi|21102387|gb|BQ414700.1|BQ414700:101:359:- 259(nt) -60.90(kcal/mol)

CTAAGAAATATGGTTTTCAAGTGGGATTTGCTGATAATATGTTGAAGAAGAATTTGCTTGCCTATTTTGCTATTCAAAATCTGCCCAAATCGACCGTAAAAATATAGCTTCCCAAGAGCAAATGTTGGTGTTGCTGTTGCAGGCTTGCTGCAGTCTACAAAAATATTGGCTTTAATGTGTTCGTACTTGACTTCGGATATCTATATTTACTTTTCTGTATGTGTGAAGTTTATCTTATTTGAGATTAATACTTTGTTAA Mar-F-1-m0035 6

.(((.((((((...(((((((((((((..(((....(((((..(((((.((.((((...((..((((((.....))))))..)).))))))........((((((((.......(((((.((((((..(((.(((((.((......)).)))))..)))..)))))).)))))...(((((((..........)))))))))))))))..)))))..)))))....)))..)))))))))))))...))).))).))).

**********TGGTTTTCAAGTGGGATTTGCTG********************************************************************************************************************************************************************************************************************************** Mar-F-1-m0035-5p 6

----------TGGTTTTCAAGTGGGATTTGC------------------------------------------------------------------------------------------------------------------------------------------------------------------------------------------------------------------------------------ t6643583 1

----------TGGTTTTCAAGTGGGATTTGCTG---------------------------------------------------------------------------------------------------------------------------------------------------------------------------------------------------------------------------------- t0385408 3

----------TGGTTTTCAAGTGGGATTTGCTGA--------------------------------------------------------------------------------------------------------------------------------------------------------------------------------------------------------------------------------- t0892194 2

//

Mar-F-1-m0038 gi|48739824|gb|CO070343.1|CO070343:231:358:- 128(nt) -58.60(kcal/mol)

GCAATATTGTTATTTACGGCTTCAGAAACCATCCCTTCCTTGCTTAATTTATGGAATCTGTAATATACAGATTGACGCCCCTTGCAAGGAAGGAATGGTTTCTGAAGCTTTAGGTAACTTTATCACGA Mar-F-1-m0038 165

(..(((..((((((((.(((((((((((((((.((((((((((...........((((((((...))))))))..........)))))))))).))))))))))))))).))))))))..)))..)..

********************TTCAGAAACCATCCCTTCCTT*************************************************************************************** Mar-F-1-m0038-5p 136

***************************************************************************************GGAAGGAATGGTTTCTGAAGC******************** Mar-F-1-m0038-3p 27

----------------CGGCTTCAGAAACCATCCCTTCCT---------------------------------------------------------------------------------------- t3395141 1

-------------------CTTCAGAAACCATCCCTTCCT---------------------------------------------------------------------------------------- t5961897 1

--------------------TTCAGAAACCATCCCTTCC----------------------------------------------------------------------------------------- t1292541 1

--------------------TTCAGAAACCATCCCTTCCT---------------------------------------------------------------------------------------- t4355443 1

--------------------TTCAGAAACCATCCCTTCCTT--------------------------------------------------------------------------------------- t0002169 129

---------------------TCAGAAACCATCCCTTCCTT--------------------------------------------------------------------------------------- t1919606 1

----------------------CAGAAACCATCCCTTCCTT--------------------------------------------------------------------------------------- t0533959 3

-------------------------------------------------------------------------------------AAGGAAGGAATGGTTTCT------------------------- t7153652 1

---------------------------------------------------------------------------------------GGAAGGAATGGTTTCTGAAG--------------------- t0928424 2

---------------------------------------------------------------------------------------GGAAGGAATGGTTTCTGAAGC-------------------- t0024471 24

---------------------------------------------------------------------------------------------AATGGTTTCTGAAGCTTTAGGTAA----------- t2497314 1

//

mireap-plant

Mar-F-1-m0039 gi|48739824|gb|CO070343.1|CO070343:423:531:- 109(nt) -52.50(kcal/mol)

ACTGTTATCTACAGCTTTAGAAATCATCCCTTTTTTAGATGTGTAAATATATAAATAAATTGAAGCACATTGCTAGAAAGGGATGATTTCTAAAGCTCTAGATAACGTT Mar-F-1-m0039 3172

...((((((((.(((((((((((((((((((((((..(((((((.(((.((....)).)))...)))))))...))))))))))))))))))))))).))))))))...

**********ACAGCTTTAGAAATCATCCCT****************************************************************************** Mar-F-1-m0039-5p 3138

********************************************************************************GGATGATTTCTAAAGCTCTAG******** Mar-F-1-m0039-3p 22

--TGTTATCTACAGCTTTAGAAA-------------------------------------------------------------------------------------- t0865090 2

-----TATCTACAGCTTTAGAAATCA----------------------------------------------------------------------------------- t4943403 1

-------TCTACAGCTTTAGAAATCA----------------------------------------------------------------------------------- t0370576 3

-------TCTACAGCTTTAGAAATCAT---------------------------------------------------------------------------------- t4328656 1

-------TCTACAGCTTTAGAAATCATC--------------------------------------------------------------------------------- t0009195 49

--------CTACAGCTTTAGAAATCATCC-------------------------------------------------------------------------------- t0772201 2

---------TACAGCTTTAGAAATCATCCC------------------------------------------------------------------------------- t0059218 13

----------ACAGCTTTAGAAATCATC--------------------------------------------------------------------------------- t0003115 102

----------ACAGCTTTAGAAATCATCC-------------------------------------------------------------------------------- t0015429 34

----------ACAGCTTTAGAAATCATCCC------------------------------------------------------------------------------- t0007522 56

----------ACAGCTTTAGAAATCATCCCT------------------------------------------------------------------------------ t0000033 2427

----------ACAGCTTTAGAAATCATCCCTT----------------------------------------------------------------------------- t0038481 17

----------ACAGCTTTAGAAATCATCCCTTT---------------------------------------------------------------------------- t6915954 1

----------ACAGCTTTAGAAATCATCCCTTTT--------------------------------------------------------------------------- t2303906 1

-----------CAGCTTTAGAAATCATCC-------------------------------------------------------------------------------- t8234987 1

-----------CAGCTTTAGAAATCATCCC------------------------------------------------------------------------------- t0198553 5

-----------CAGCTTTAGAAATCATCCCT------------------------------------------------------------------------------ t0033566 19

-----------CAGCTTTAGAAATCATCCCTT----------------------------------------------------------------------------- t0000360 400

-----------CAGCTTTAGAAATCATCCCTTT---------------------------------------------------------------------------- t0315738 4

------------AGCTTTAGAAATCATCCCT------------------------------------------------------------------------------ t4393133 1

------------AGCTTTAGAAATCATCCCTT----------------------------------------------------------------------------- t1029074 2

---------------TTTAGAAATCATCCCTTTTTT------------------------------------------------------------------------- t0202477 5

---------------------------------------------------------------------------GAAAGGGATGATTTCTAAAGC------------- t5039291 1

----------------------------------------------------------------------------AAAGGGATGATTTCTAAAG-------------- t4143253 1

----------------------------------------------------------------------------AAAGGGATGATTTCTAAAGCT------------ t5683785 1

-----------------------------------------------------------------------------AAGGGATGATTTCTAAAG-------------- t3083164 1

-----------------------------------------------------------------------------AAGGGATGATTTCTAAAGCT------------ t2385601 1

-----------------------------------------------------------------------------AAGGGATGATTTCTAAAGCTCTA--------- t3045672 1

-----------------------------------------------------------------------------AAGGGATGATTTCTAAAGCTCTAG-------- t0121395 7

------------------------------------------------------------------------------AGGGATGATTTCTAAAGC------------- t0409894 3

------------------------------------------------------------------------------AGGGATGATTTCTAAAGCT------------ t0286054 4

-------------------------------------------------------------------------------GGGATGATTTCTAAAGCT------------ t1208763 2

--------------------------------------------------------------------------------GGATGATTTCTAAAGCTCT---------- t7280231 1

----------------------------------------------------------------------------------ATGATTTCTAAAGCTCTAGAT------ t1062758 2

----------------------------------------------------------------------------------------TCTAAAGCTCTAGATAACGTT t4229584 1

//

Mar-F-1-m0040 gi|48752263|gb|CO082782.1|CO082782:157:290:+ 134(nt) -55.00(kcal/mol)

TGGGAGGTGTTGGTTGAAGAGGAGGCACTGGTGATTGTTGTTGACAGAAGATAGAGAGCACAGATGATGGTGTTGCCTATGTGCATGCATCCCACTCCTTTGTGCTCTCTATGCTTCTGTCATCATCACCTTTC Mar-F-1-m0040 21

.....((((((..((....))..))))))((((((.(....(((((((((((((((((((((((.(((((((((((......))).)))..))).)).)))))))))))))).))))))))).)))))))....

*******************************************************************************************************GCTCTCTATGCTTCTGTCATC********** Mar-F-1-m0040-3p 21

-------------------------------------------------------------------------------------------------------GCTCTCTATGCTTCTGTCATC---------- t0037117 18

-------------------------------------------------------------------------------------------------------GCTCTCTATGCTTCTGTCATCA--------- t0528370 3

//

mireap-plant

Mar-F-1-m0041 gi|48790494|gb|CO091808.1|CO091808:438:656:+ 219(nt) -49.50(kcal/mol)

ATTGATCTACATATGTTAGATCAAAGAGTAAATTAGCTATTTTTTTTTCAAAATTTCATCCATTTATACTATTAAAAACAACGTGGTTGACAGAATAATGGGACAATGACACGTACCATATCATGTGTACATGTAAAGATCTATTTTAAATAGCAAAAATAAATGAATTTTTAATAGAATGGTCTATTTACTTTTTAATCTAATATATAAGCATCAATT Mar-F-1-m0041 9

((((((((..(((((((((((.(((((((((((..((((((((...((.((((.(((((..((((...(((((.((((....((.((((.((......))...)))).))..(((((((...))).)))).............)))).)))))...))))..))))).)))))).))))))))..))))))))))).))))))))))).)).)))))).

**********ATATGTTAGATCAAAGAGTAA******************************************************************************************************************************************************************************************** Mar-F-1-m0041-5p 9

----------ATATGTTAGATCAAAGAGT---------------------------------------------------------------------------------------------------------------------------------------------------------------------------------------------- t7706287 1

----------ATATGTTAGATCAAAGAGTAA-------------------------------------------------------------------------------------------------------------------------------------------------------------------------------------------- t0635856 2

----------ATATGTTAGATCAAAGAGTAAA------------------------------------------------------------------------------------------------------------------------------------------------------------------------------------------- t6791840 1

----------ATATGTTAGATCAAAGAGTAAATT----------------------------------------------------------------------------------------------------------------------------------------------------------------------------------------- t8173330 1

-----------TATGTTAGATCAAAGAGTAAA------------------------------------------------------------------------------------------------------------------------------------------------------------------------------------------- t0981605 2

-----------TATGTTAGATCAAAGAGTAAAT------------------------------------------------------------------------------------------------------------------------------------------------------------------------------------------ t4342629 1

-----------TATGTTAGATCAAAGAGTAAATT----------------------------------------------------------------------------------------------------------------------------------------------------------------------------------------- t3532453 1

//

mireap-plant

Mar-F-1-m0042 gi|48793670|gb|CO094984.1|CO094984:68:167:+ 100(nt) -37.55(kcal/mol)

GGAAGTCTTCGGCTGTGGTTGATTCGGCAAGAAAATATAACTTACTTGTCTTTTTTATTTTTATTTTTCTTGCCGATTCCACCCATGCCTACGATTTCCT Mar-F-1-m0042 164

(((((((...(((((.(((.((.((((((((((((.............................)))))))))))).)).))))).)))...))))))).

**********GGCTGTGGTTGATTCGGCAAGA******************************************************************** Mar-F-1-m0042-5p 162

-----TCTTCGGCTGTGGTTGATTCGGCA----------------------------------------------------------------------- t7909015 1

--------TCGGCTGTGGTTGATTCGGCA----------------------------------------------------------------------- t0311803 4

--------TCGGCTGTGGTTGATTCGGCAAG--------------------------------------------------------------------- t4384217 1

--------TCGGCTGTGGTTGATTCGGCAAGA-------------------------------------------------------------------- t5429040 1

--------TCGGCTGTGGTTGATTCGGCAAGAA------------------------------------------------------------------- t1537350 1

---------CGGCTGTGGTTGATTCGGCAA---------------------------------------------------------------------- t0651610 2

---------CGGCTGTGGTTGATTCGGCAAG--------------------------------------------------------------------- t0014981 34

---------CGGCTGTGGTTGATTCGGCAAGA-------------------------------------------------------------------- t0026310 23

---------CGGCTGTGGTTGATTCGGCAAGAA------------------------------------------------------------------- t0928300 2

----------GGCTGTGGTTGATTCGGCAAG--------------------------------------------------------------------- t0047358 15

----------GGCTGTGGTTGATTCGGCAAGA-------------------------------------------------------------------- t0005207 73

----------GGCTGTGGTTGATTCGGCAAGAA------------------------------------------------------------------- t4260118 1

----------GGCTGTGGTTGATTCGGCAAGAAA------------------------------------------------------------------ t7226855 1

----------GGCTGTGGTTGATTCGGCAAGAAAA----------------------------------------------------------------- t6885987 1

-----------GCTGTGGTTGATTCGGCAAGA-------------------------------------------------------------------- t0422766 3

------------------TTGATTCGGCAAGAAAATATA------------------------------------------------------------- t3376130 1

//

mireap-plant

Mar-F-1-m0043 gi|48793670|gb|CO094984.1|CO094984:258:350:+ 93(nt) -37.39(kcal/mol)

AAATTTTCGGAATGGAGGAGTTGGAAAGATTTTGAATTTTCTCTGTATTTTAATAATGAAAAATCTTTCCAATTCCTCCCATTCCACTGATTT Mar-F-1-m0043 110

........(((((((((((((((((((((((((..(((................)))..)))))))))))))))))).)))))))........

**********AATGGAGGAGTTGGAAAGATT************************************************************** Mar-F-1-m0043-5p 73

***************************************************************TCTTTCCAATTCCTCCCATTCC******** Mar-F-1-m0043-3p 36

-------CGGAATGGAGGAGTTGGAA------------------------------------------------------------------- t2730257 1

-------CGGAATGGAGGAGTTGGAAA------------------------------------------------------------------ t6575607 1

-------CGGAATGGAGGAGTTGGAAAG----------------------------------------------------------------- t6441165 1

--------GGAATGGAGGAGTTGGAAAGA---------------------------------------------------------------- t3232568 1

----------AATGGAGGAGTTGGAAAGA---------------------------------------------------------------- t1239567 2

----------AATGGAGGAGTTGGAAAGATT-------------------------------------------------------------- t0007144 59

-----------ATGGAGGAGTTGGAAAGATT-------------------------------------------------------------- t0539237 3

-----------ATGGAGGAGTTGGAAAGATTT------------------------------------------------------------- t0529362 3

------------TGGAGGAGTTGGAAAGATT-------------------------------------------------------------- t0928256 2

---------------------------------------------------------------TCTTTCCAATTCCTCCCATTCC-------- t0064963 12

----------------------------------------------------------------CTTTCCAATTCCTCCCATTCC-------- t2674737 1

-----------------------------------------------------------------TTTCCAATTCCTCCCATTCCAC------ t0026338 23

---------------------------------------------------------------------CAATTCCTCCCATTCCACTGATT- t4444176 1

//

Mar-F-1-m0044 gi|48800227|gb|CO101541.1|CO101541:747:821:- 75(nt) -27.10(kcal/mol)

TGATTACCAGTAGCTGTGGCAGTAATAAACTCTGCTTCGGGCAGTGGATTGGGCTACAGTTTCTTGGTTTTTCGA Mar-F-1-m0044 14

.((..(((((.(((((((((..((((...(.((((.....)))).).)))).)))))))))..)))))...))..

******************************************AGTGGATTGGGCTACAGTTTCTT********** Mar-F-1-m0044-3p 14

----------------------------------------GCAGTGGATTGGGCTACAGTTTCT----------- t0865476 2

-----------------------------------------CAGTGGATTGGGCTACAGTTTCTT---------- t1203172 2

------------------------------------------AGTGGATTGGGCTACAGTTTCTT---------- t0150179 6

------------------------------------------AGTGGATTGGGCTACAGTTTCTTG--------- t0474304 3

-------------------------------------------GTGGATTGGGCTACAGTTTCTTGG-------- t3078909 1

//

Mar-F-1-m0048 gi|48827796|gb|CO126186.1|CO126186:120:220:- 101(nt) -33.90(kcal/mol)

GTTTTGTTACATAAAATACTGATGTGACATAATTGATCAATATTAAATTGTGATTTTAGTATTAGTTGATCATGTCCCGTTAGTATTTTATGTTTTGTTTT Mar-F-1-m0048 6

........((((((((((((((((.(((((.((..((.(((((((((.......))))))))).))..)).))))).))))))))))))))))........

**********ATAAAATACTGATGTGACATA********************************************************************** Mar-F-1-m0048-5p 6

----------ATAAAATACTGATGTGACATA---------------------------------------------------------------------- t0161644 6

//

Mar-F-1-m0051 gi|73850013|gb|DT459033.1|DT459033:165:304:- 140(nt) -28.42(kcal/mol)

GAAGTTGACGACGGTTTTAAGTTTTAACTGAAACTTTATGGATGGAACCCCAAGAGCTTTTTGTTGTTTGTTTATCTTCTATCATCATGATTCATGAATTTGATGCAATCCATGTTAATATGTAAAACCTTGTTATCTAT Mar-F-1-m0051 9

..((.((((((.(((((((.((.(((((((..........(((((((......((((........)))).......))))))).(((((...))))).............)).))))).)).))))))))))))).))..

**********ACGGTTTTAAGTTTTAACTGA************************************************************************************************************* Mar-F-1-m0051-5p 9

---------GACGGTTTTAAGTTTTAACTG-------------------------------------------------------------------------------------------------------------- t0337954 4

----------ACGGTTTTAAGTTTTAACT--------------------------------------------------------------------------------------------------------------- t4178257 1

----------ACGGTTTTAAGTTTTAACTGA------------------------------------------------------------------------------------------------------------- t0303782 4

//

mireap-plant

Mar-F-1-m0052 gi|73850014|gb|DT459034.1|DT459034:592:733:+ 142(nt) -28.42(kcal/mol)

TGAAGTTGACGACGGTTTTAAGTTTTAACTGAAACTTTATGGATGGAACCCCAAGAGCTTTTTGTTGTTTGTTTATCTTCTATCATCATGATTCATGAATTTGATGCAATCCATGTTAATATGTAAAACCTTGTTATCTATC Mar-F-1-m0052 9

...((.((((((.(((((((.((.(((((((..........(((((((......((((........)))).......))))))).(((((...))))).............)).))))).)).))))))))))))).))...

**********GACGGTTTTAAGTTTTAACTG*************************************************************************************************************** Mar-F-1-m0052-5p 9

----------GACGGTTTTAAGTTTTAACTG--------------------------------------------------------------------------------------------------------------- t0337954 4

-----------ACGGTTTTAAGTTTTAACT---------------------------------------------------------------------------------------------------------------- t4178257 1

-----------ACGGTTTTAAGTTTTAACTGA-------------------------------------------------------------------------------------------------------------- t0303782 4

//

mireap-plant

Mar-F-1-m0053 gi|73863731|gb|DT466470.1|DT466470:332:400:- 69(nt) -20.80(kcal/mol)

ATGCGTGCAATTGGCGAACAAATCAGTAGGAGTTTTGCCTCCGATGTGGTAGTTCACCAGGGCATCCAA Mar-F-1-m0053 23

.((.((((..((((.((((..((((...((((......))))....)))).)))).)))).)))).)).

**********TTGGCGAACAAATCAGTAGGAGT************************************ Mar-F-1-m0053-5p 12

***************************************TCCGATGTGGTAGTTCACCAGGG******* Mar-F-1-m0053-3p 10

-------CAATTGGCGAACAAATCAGT------------------------------------------ t6746009 1

-------CAATTGGCGAACAAATCAGTA----------------------------------------- t1553957 1

---------ATTGGCGAACAAATCAGTA----------------------------------------- t4823577 1

---------ATTGGCGAACAAATCAGTAGGAGT------------------------------------ t1015012 2

----------TTGGCGAACAAATCAGTA----------------------------------------- t0741923 2

----------TTGGCGAACAAATCAGTAGGAG------------------------------------- t0646359 2

----------TTGGCGAACAAATCAGTAGGAGT------------------------------------ t0523849 3

--------------------------------------CTCCGATGTGGTAGTTCACCAGGG------- t2190559 1

---------------------------------------TCCGATGTGGTAGTTCACCAGGGC------ t0899442 2

----------------------------------------CCGATGTGGTAGTTCACCAGGGCA----- t0355036 3

-----------------------------------------CGATGTGGTAGTTCACCAGGGCAT---- t0317249 4

---------------------------------------------GTGGTAGTTCACCAGGGCATCCAA t3345001 1

//

mireap-plant

Mar-F-1-m0054 gi|78323093|gb|DT543367.1|DT543367:746:896:- 151(nt) -30.30(kcal/mol)

TCTCCACTTAACGACAGAAAAAAGGATTGATCAAATTCTATTGAGTCTGATATATTTATACTTTTAAATAGAATAGCTCCACAGCTGCACACAATCATCGACAAACTCAAGTTCTTGTTCATCTTTTTCATATCCACTGTGAGGCGGAGAC Mar-F-1-m0054 9

(((((.(((.(((...((.((((((((.((.(((......((((((.((...((((((......))))))...(((((....)))))..............)).))))))....))).))))))))))....))...))).))).))))).

**********ACGACAGAAAAAAGGATTGATCA********************************************************************************************************************** Mar-F-1-m0054-5p 8

----------ACGACAGAAAAAAGGATTGATCA---------------------------------------------------------------------------------------------------------------------- t0268820 4

----------ACGACAGAAAAAAGGATTGATCAA--------------------------------------------------------------------------------------------------------------------- t0400581 3

-----------CGACAGAAAAAAGGATTGATCAAA-------------------------------------------------------------------------------------------------------------------- t7676025 1

---------------------------------AATTCTATTGAGTCTGATATATTT---------------------------------------------------------------------------------------------- t1301406 1

//

Mar-F-1-m0055 gi|84168871|gb|DR457519.1|DR457519:174:279:+ 106(nt) -40.90(kcal/mol)

CGGGAGTTTATGGAAGTGGGATGGGTGGAAAGATTTGTATTTTCTCTCTTGAGTTTCTAAGAATGTAAAGCAAATCTTTCCTACTCCTCCCATACCACTTATTTCC Mar-F-1-m0055 30

.((((((...(((..((((((.((((((((((((((((.(((.(..(((((......)))))..).))))))))))))))).)))).)))))).)))...))))))

*************AAGTGGGATGGGTGGAAAGATT*********************************************************************** Mar-F-1-m0055-5p 21

**************************************************************************TCTTTCCTACTCCTCCCATACC********** Mar-F-1-m0055-3p 8

-------------AAGTGGGATGGGTGGAAAGA------------------------------------------------------------------------- t1609439 1

-------------AAGTGGGATGGGTGGAAAGAT------------------------------------------------------------------------ t2101993 1

-------------AAGTGGGATGGGTGGAAAGATT----------------------------------------------------------------------- t0045639 15

--------------AGTGGGATGGGTGGAAAGATTTG--------------------------------------------------------------------- t0712278 2

----------------TGGGATGGGTGGAAAGATTTG--------------------------------------------------------------------- t0636669 2

--------------------ATGGGTGGAAAGATTTGTAT------------------------------------------------------------------ t6473503 1

--------------------------------------------------------------------------TCTTTCCTACTCCTCCCATACC---------- t0111823 8

//

mireap-plant

Mar-F-2-m0006 gi|109858124|gb|DW487103.1|DW487103:254:398:- 145(nt) -36.69(kcal/mol)

AGAAATGGTTGGAGGATCTCCAGGACTTGGCTTACGATGTGGATGACATCTTGGATGAGTTCGCTTATGAAGAATTTCTCTTTTCAACTCCTTTCTCATAATTCCAACATGTCTGTCATTGGAGAGGCTGCTCTTTCTGGGTTCC Mar-F-2-m0006 12

.............(((.(.((((((..((((((.(((((.....(((((.(((((((((........((((((.......))))))........))))....))))).)))))...)))))...))))))....))))))).)))

**********GGAGGATCTCCAGGACTTGGCTT**************************************************************************************************************** Mar-F-2-m0006-5p 12

---------TGGAGGATCTCCAGGACTTGGCTT---------------------------------------------------------------------------------------------------------------- t0680501 3

----------GGAGGATCTCCAGGACTTGGCT----------------------------------------------------------------------------------------------------------------- t4040572 1

----------GGAGGATCTCCAGGACTTGGCTT---------------------------------------------------------------------------------------------------------------- t0234573 5

----------GGAGGATCTCCAGGACTTGGCTTA--------------------------------------------------------------------------------------------------------------- t0529499 3

//

Mar-F-2-m0014 gi|109864468|gb|DW493444.1|DW493444:294:395:+ 102(nt) -42.80(kcal/mol)

AACGCCACAAATCCCACCTTTTCCCCAAATCTTTTAAAGTCCATGAACAACATGTTTGTGTTGTCTAAAAAGATTTGGGGCAAAGACGGGATTAGTGGGAAA Mar-F-2-m0014 224

....((((.((((((..((((.(((((((((((((..((.(((..(((.....)))..))..).)).))))))))))))).))))..)))))).))))....

***********************************************************************GATTTGGGGCAAAGACGGGAT********** Mar-F-2-m0014-3p 219

--------------------------------------------------------------------AAAGATTTGGGGCAAAGA---------------- t0920929 2

--------------------------------------------------------------------AAAGATTTGGGGCAAAGACGG------------- t0150791 8

----------------------------------------------------------------------AGATTTGGGGCAAAGACGGGAT---------- t4641969 1

-----------------------------------------------------------------------GATTTGGGGCAAAGACGGGA----------- t1313025 2

-----------------------------------------------------------------------GATTTGGGGCAAAGACGGGAT---------- t0001491 189

------------------------------------------------------------------------ATTTGGGGCAAAGACGGGATT--------- t1683302 1

-------------------------------------------------------------------------TTTGGGGCAAAGACGGGAT---------- t2975882 1

-------------------------------------------------------------------------TTTGGGGCAAAGACGGGATTA-------- t0097238 10

--------------------------------------------------------------------------TTGGGGCAAAGACGGGAT---------- t1357865 2

--------------------------------------------------------------------------TTGGGGCAAAGACGGGATTAG------- t0485283 3

---------------------------------------------------------------------------TGGGGCAAAGACGGGATTAG------- t6213883 1

---------------------------------------------------------------------------TGGGGCAAAGACGGGATTAGT------ t1299103 2

---------------------------------------------------------------------------TGGGGCAAAGACGGGATTAGTG----- t1226259 2

//

Mar-F-2-m0016 gi|109864995|gb|DW493971.1|DW493971:195:305:- 111(nt) -51.70(kcal/mol)

AGTTAAGGCGCTGGATGCAGAGGTTTATCGATCGATTCATGTATTTGTGTTATTCTCACAACAAAATTCTCAGGAAGAGATCGATGAACCGCTGCCTCCAGTGTCTTTACC Mar-F-2-m0016 7

.((.(((((((((((.((((.((((((((((((..(((.((.((((.((((........))))))))...)).)))..)))))))))))).)))).))))))))))).)).

**********CTGGATGCAGAGGTTTATCGA******************************************************************************** Mar-F-2-m0016-5p 7

----------CTGGATGCAGAGGTTTATCGA-------------------------------------------------------------------------------- t0218950 6

-----------TGGATGCAGAGGTTTATCGAT------------------------------------------------------------------------------- t5951554 1

//

Mar-F-2-m0019 gi|109867926|gb|DW496902.1|DW496902:180:262:+ 83(nt) -28.84(kcal/mol)

GGGCCACTTCGTGATTGGGCTAGGGTCTAGGCAAATTGGGTTTAAATTTTAATGGGCCCTAGTGGATTAGCAATTTGGGCCTG Mar-F-2-m0019 11

(((((......(((((.(.((((((((((...(((((.......)))))...))))))))))).)))))........))))).

**********GTGATTGGGCTAGGGTCTAGGCA************************************************** Mar-F-2-m0019-5p 11

-------TTCGTGATTGGGCTAGGGTCTAGG---------------------------------------------------- t1031725 2

-------TTCGTGATTGGGCTAGGGTCTAGGC--------------------------------------------------- t3940826 1

--------TCGTGATTGGGCTAGGGTCTAGGC--------------------------------------------------- t0969329 2

----------GTGATTGGGCTAGGGTCTAGGCA-------------------------------------------------- t0482159 3

----------GTGATTGGGCTAGGGTCTAGGCAA------------------------------------------------- t0675386 3

//

Mar-F-2-m0020 gi|109867949|gb|DW496925.1|DW496925:69:199:+ 131(nt) -39.70(kcal/mol)

TTTTGGGCCATCGGGCCTGTATTTGGGTCTTGGTTTTAAATTTGGGTCTAGGTTTAGTTATTGGGCTTGGGTTATGTAGCTGGGTTTGGGTCTGTTATAAAACTGGACTGTTGTATTGGTTTGGTTTAATT Mar-F-2-m0020 9

..((((((((...((((.((((...(((((.(((((((....(((..(((((..(((((((..(((....)))..)))))))..)))))..)))...))))))))))))...)))).))))))))))))..

**************************************************************************************************AAAACTGGACTGTTGTATTGGTT********** Mar-F-2-m0020-3p 9

-------------------------------------------------------------------------------------------------TAAAACTGGACTGTTGTATTGGTT---------- t4874724 1

--------------------------------------------------------------------------------------------------AAAACTGGACTGTTGTATTGGTT---------- t0609767 3

--------------------------------------------------------------------------------------------------AAAACTGGACTGTTGTATTGGTTT--------- t1471675 2

---------------------------------------------------------------------------------------------------AAACTGGACTGTTGTATTGGT----------- t5450880 1

---------------------------------------------------------------------------------------------------AAACTGGACTGTTGTATTGGTT---------- t5411879 1

----------------------------------------------------------------------------------------------------AACTGGACTGTTGTATTGGTTT--------- t8384632 1

//

Mar-F-2-m0022 gi|109875138|gb|DW504112.1|DW504112:81:378:+ 298(nt) -95.50(kcal/mol)

TTTCTTCTCTTTAGATTCATTGGCTGAGTTAAAACAGCAGCACAAATTTCGGGGTCAAGAATTTTATATGCAGGTGAAAAAACCACTTTTAACTTAAAAATATTCTTTTTCCGCAAAAATACCGGAAAACGCTAGTGTTTTAAAGTTAAATATGCGATTAAAAAAACCTTTCTACCCAAATATTGAGAAGTTGGTTAAAAGAATTTTAAACTCGACATCGAGTCAAAGTCATCGAGTTTAAGACCTAAAACCAAATATCTGAATATTAACTCAGCTAATGAATCAAAAGAGAAGAAAG Mar-F-2-m0022 8

((((((((((((.((((((((((((((((((....................((((..(((((((((..(((((((......)))...((((((((.(((((((((.(((((((.........))))))).)..)))))))).))))))))..))))..)))))......))))))))..(((((.(((..((((((...((..((((((((((((...(........)...)))))))))))).))..))))))...))).))))))))))))))))))))))).)))))))))))).

**********TTAGATTCATTGGCTGAGTTA*************************************************************************************************************************************************************************************************************************************************************************** Mar-F-2-m0022-5p 8

-------TCTTTAGATTCATTGGCTGAG------------------------------------------------------------------------------------------------------------------------------------------------------------------------------------------------------------------------------------------------------------------------------ t3443510 1

----------TTAGATTCATTGGCTGAGT----------------------------------------------------------------------------------------------------------------------------------------------------------------------------------------------------------------------------------------------------------------------------- t4864600 1

----------TTAGATTCATTGGCTGAGTTA--------------------------------------------------------------------------------------------------------------------------------------------------------------------------------------------------------------------------------------------------------------------------- t0202422 6

//

Mar-F-2-m0034 gi|164256299|gb|ES794470.1|ES794470:1:155:+ 155(nt) -25.30(kcal/mol)

ATGGGGTAGTTTGAATTCTTATTGAGGCTTTTTCTTCCTAAATTATTTATTTATTTCATATAGCATATAGAAGGAAATTGATTTCATATAGAAAGTATAGATAGATTACTATGTATCGACTGAACCAATGACTATTCATGATTCCATAATTGAAT Mar-F-2-m0034 65

(((((((....(((((..((((((..(((((((......((((((...((((.((((.((((...)))))))).)))))))))).....)))))))...((((.((....)).))))........))))))..)))))..)))))))........

**************************************************************************************************************************AACCAATGACTATTCATGATTCC********** Mar-F-2-m0034-3p 65

------------------------------------------------------------------------------------------------------------------------TGAACCAATGACTATTCATGATTC----------- t1356022 2

------------------------------------------------------------------------------------------------------------------------TGAACCAATGACTATTCATGATTCC---------- t0946166 2

-------------------------------------------------------------------------------------------------------------------------GAACCAATGACTATTCATGATT------------ t2321978 1

-------------------------------------------------------------------------------------------------------------------------GAACCAATGACTATTCATGATTCC---------- t0714398 2

--------------------------------------------------------------------------------------------------------------------------AACCAATGACTATTCATG--------------- t2776401 1

--------------------------------------------------------------------------------------------------------------------------AACCAATGACTATTCATGA-------------- t2119371 1

--------------------------------------------------------------------------------------------------------------------------AACCAATGACTATTCATGAT------------- t0191936 6

--------------------------------------------------------------------------------------------------------------------------AACCAATGACTATTCATGATT------------ t0053189 16

--------------------------------------------------------------------------------------------------------------------------AACCAATGACTATTCATGATTC----------- t0161037 7

--------------------------------------------------------------------------------------------------------------------------AACCAATGACTATTCATGATTCC---------- t0045591 18

--------------------------------------------------------------------------------------------------------------------------AACCAATGACTATTCATGATTCCA--------- t0137282 8

---------------------------------------------------------------------------------------------------------------------------ACCAATGACTATTCATGATTCC---------- t3254049 1

//

mireap-plant

Mar-F-2-m0035 gi|164277687|gb|ES792109.1|ES792109:536:620:- 85(nt) -18.33(kcal/mol)

AGCCCAAAAATCCTCAATTTCTAGGGTTCTTACCGATTCATCCATACTCTTTTTTGGGAGAAATTAGATTGCCGAAATTTGGGGA Mar-F-2-m0035 7

..((((((..((..(((..(((((..(((((.((((.................))))))))).))))))))..))..))))))..

*******************************************************GGGAGAAATTAGATTGCCGA********** Mar-F-2-m0035-3p 7

----------------------------------------------------TTTGGGAGAAATTAGATTGCCGAA--------- t2413532 1

-------------------------------------------------------GGGAGAAATTAGATTGCCGA---------- t0304186 5

-------------------------------------------------------GGGAGAAATTAGATTGCCGAAAT------- t8160526 1

//

Mar-F-2-m0039 gi|164305711|gb|ES850612.1|ES850612:220:314:- 95(nt) -20.00(kcal/mol)

TATCAAATCACCGGCATTTAGCCTGCTAGGTTTTAAATCCGATAATATTATTCACCTGCTTAAAGCTTGCTAGGCTCAAAGCCCAAATATCACCA Mar-F-2-m0039 11

............(((.((.((((((.((((((((((.......................)))))))))).)))))).)).)))............

**************************************************************AAGCTTGCTAGGCTCAAAGCCCA********** Mar-F-2-m0039-3p 10

--------CACCGGCATTTAGCCTGCTAGGTT--------------------------------------------------------------- t7165294 1

-------------------------------------------------------------AAAGCTTGCTAGGCTCAAAGCCCA---------- t1038422 2

--------------------------------------------------------------AAGCTTGCTAGGCTCAAAGCCC----------- t6665285 1

--------------------------------------------------------------AAGCTTGCTAGGCTCAAAGCCCA---------- t0431237 4

--------------------------------------------------------------AAGCTTGCTAGGCTCAAAGCCCAA--------- t7318276 1

---------------------------------------------------------------AGCTTGCTAGGCTCAAAGCCCA---------- t6887784 1

---------------------------------------------------------------AGCTTGCTAGGCTCAAAGCCCAAA-------- t2962334 1

//

Mar-F-2-m0043 gi|164355292|gb|ES817398.1|ES817398:531:729:- 199(nt) -56.80(kcal/mol)

TAAAAGTGAGAGATTTGAAAATTTTGGTATTCCAAGAATGAGCCCATTGAGAGTTAATAGGAGTTCTAGGAAAAAAAATCTAATAGCTACAAGCTAGACGTGAAGAATATCAGACATTGGGCATTTGGTCTAGTGGTATGATTCTCGCTTTGGGTGCGAGAGGTCCCGAGTTCGATTCTCGGAATGCCCCAGCACTTTT Mar-F-2-m0043 11

.(((((((.((((((....))))))((((((((.(((((((((.....((((((((...((.(((((.(((.......)))..((((.....)))).......))))).))...((((((((.....))))))))...))))))))...(((((..(....)..))))))))).))))).))))))))....)))))))

*************************************************************************************************************************************************************************GTTCGATTCTCGGAATGCCC********** Mar-F-2-m0043-3p 11

------------------------------------------------------------------------------------------------------------------------------------------------------------------------AGTTCGATTCTCGGAATG------------- t3882421 1

------------------------------------------------------------------------------------------------------------------------------------------------------------------------AGTTCGATTCTCGGAATGC------------ t5104453 1

------------------------------------------------------------------------------------------------------------------------------------------------------------------------AGTTCGATTCTCGGAATGCC----------- t0933944 2

------------------------------------------------------------------------------------------------------------------------------------------------------------------------AGTTCGATTCTCGGAATGCCC---------- t6641666 1

-------------------------------------------------------------------------------------------------------------------------------------------------------------------------GTTCGATTCTCGGAATGCC----------- t1366045 2

-------------------------------------------------------------------------------------------------------------------------------------------------------------------------GTTCGATTCTCGGAATGCCC---------- t0909635 2

---------------------------------------------------------------------------------------------------------------------------------------------------------------------------TCGATTCTCGGAATGCCC---------- t3747704 1

----------------------------------------------------------------------------------------------------------------------------------------------------------------------------CGATTCTCGGAATGCCCC--------- t3460802 1

//

Mar-F-2-m0046 gi|195983222|gb|FL576404.1|FL576404:137:236:- 100(nt) -28.60(kcal/mol)

CAAGAATAGTGGCCTTGAGTTTCTCGACCCTTTGACTTAGGATTAGTCAGTTCTATTTCTTGATGGGGGAAGGGATATAACTCAGCGGTAGAGTGTCACC Mar-F-2-m0046 14

...((......((((((((((((((..(((((.((..(((((((....)))))))..)).....)))))..))))...))))))).)))......))...

*******************************************************************GGAAGGGATATAACTCAGCGGTA********** Mar-F-2-m0046-3p 14

------------------------------------------------------------------GGGAAGGGATATAACTCAGCGGTA---------- t0291972 5

-------------------------------------------------------------------GGAAGGGATATAACTCAGCGGTA---------- t0238298 5

-------------------------------------------------------------------GGAAGGGATATAACTCAGCGGTAG--------- t0366358 4

//

Mar-F-2-m0051 gi|48739824|gb|CO070343.1|CO070343:425:529:+ 105(nt) -47.85(kcal/mol)

CGTTATCTAGAGCTTTAGAAATCATCCCTTTCTAGCAATGTGCTTCAATTTATTTATATATTTACACATCTAAAAAAGGGATGATTTCTAAAGCTGTAGATAACA Mar-F-2-m0051 17

.((((((((.(((((((((((((((((((((.(((..(((((......................))))))))..))))))))))))))))))))).)))))))).

************CTTTAGAAATCATCCCTTTCT************************************************************************ Mar-F-2-m0051-5p 5

**************************************************************************AAAGGGATGATTTCTAAAGCT********** Mar-F-2-m0051-3p 12

---------GAGCTTTAGAAATCATCCCTT--------------------------------------------------------------------------- t0717540 2

----------AGCTTTAGAAATCATCCCT---------------------------------------------------------------------------- t3290352 1

----------AGCTTTAGAAATCATCCCTT--------------------------------------------------------------------------- t0985524 2

--------------------------------------------------------------------------AAAGGGATGATTTCTAAAG------------ t7053274 1

--------------------------------------------------------------------------AAAGGGATGATTTCTAAAGCT---------- t0295081 5

----------------------------------------------------------------------------AGGGATGATTTCTAAAGCT---------- t0402051 4

-----------------------------------------------------------------------------GGGATGATTTCTAAAGCT---------- t1516858 2

//

Mar-F-2-m0055 gi|48790494|gb|CO091808.1|CO091808:439:655:+ 217(nt) -48.90(kcal/mol)

TTGATCTACATATGTTAGATCAAAGAGTAAATTAGCTATTTTTTTTTCAAAATTTCATCCATTTATACTATTAAAAACAACGTGGTTGACAGAATAATGGGACAATGACACGTACCATATCATGTGTACATGTAAAGATCTATTTTAAATAGCAAAAATAAATGAATTTTTAATAGAATGGTCTATTTACTTTTTAATCTAATATATAAGCATCAAT Mar-F-2-m0055 10

(((((((..(((((((((((.(((((((((((..((((((((...((.((((.(((((..((((...(((((.((((....((.((((.((......))...)))).))..(((((((...))).)))).............)))).)))))...))))..))))).)))))).))))))))..))))))))))).))))))))))).)).))))).

**********TATGTTAGATCAAAGAGTAAATT**************************************************************************************************************************************************************************************** Mar-F-2-m0055-5p 10

---------ATATGTTAGATCAAAGAGTA-------------------------------------------------------------------------------------------------------------------------------------------------------------------------------------------- t5796067 1

---------ATATGTTAGATCAAAGAGTAA------------------------------------------------------------------------------------------------------------------------------------------------------------------------------------------- t5813093 1

---------ATATGTTAGATCAAAGAGTAAA------------------------------------------------------------------------------------------------------------------------------------------------------------------------------------------ t8524811 1

---------ATATGTTAGATCAAAGAGTAAAT----------------------------------------------------------------------------------------------------------------------------------------------------------------------------------------- t5601887 1

----------TATGTTAGATCAAAGAGTAAA------------------------------------------------------------------------------------------------------------------------------------------------------------------------------------------ t0519062 3

----------TATGTTAGATCAAAGAGTAAATT---------------------------------------------------------------------------------------------------------------------------------------------------------------------------------------- t0484402 3

//

Mar-F-2-m0058 gi|48801241|gb|CO102555.1|CO102555:195:322:- 128(nt) -42.10(kcal/mol)

TCGAGCTTGCGGATCAAACAGCTGGAAGCCAGACGGAAACAGTCCGGACTTCAGTTTATTAAGATGGGGATCTGATGAAGATAATCTGACTTGAGCATGCCTGGCTCCCTGTATGCCTGCAGGAGCAT Mar-F-2-m0058 18

..(..(((((((..((.((((..(((.(((((.((((.....)))).......(((((...((((....((((.....)))).))))....)))))....)))))))))))).)).)))))))..)..

*************************************************************************************************TGCCTGGCTCCCTGTATGCCT********** Mar-F-2-m0058-3p 18

-------------------------------------------------------------------------------------------------TGCCTGGCTCCCTGTATG------------- t2467353 1

-------------------------------------------------------------------------------------------------TGCCTGGCTCCCTGTATGC------------ t0862031 2

-------------------------------------------------------------------------------------------------TGCCTGGCTCCCTGTATGCC----------- t1762181 1

-------------------------------------------------------------------------------------------------TGCCTGGCTCCCTGTATGCCT---------- t0068442 14

//

Mar-F-2-m0064 gi|48819513|gb|CO120826.1|CO120826:120:464:+ 345(nt) -83.97(kcal/mol)

AGCCCTTTTCCCCGATGTGCTGGGTCGTGTATATACATGGTATACGACCACATACCAAGATTATTTGCTGTTGGATGAGCCTCATTTTTTCATGGCACTTATGAAATTGGAGCTTGTTTTAGTACTGCCGCTCGCTATACTTAACACTTATGGCTTGTTGACCTCCAAGCCTTGGTTCAACATCACCTGCCTCATCTTCGGTTCTGCTCTTGTTACCTCCACGGTAAATTAAATATTTACTTTTCTTCCTTGCATTAAATATTACTACTTTTTTCTTTTTGGTGAATTATAAGAAGAGGAAAAACTCTAACAGCAATACGACTAGCGCGACTCGAATTCAGGTCA Mar-F-2-m0064 8

.(.(((......(((((((((((.((((((.......((((((........))))))........((((((((((.((((.......(((((((.....)))))))..((((((((((..((((..((.....))..)))).)))).....(((.((((((...(((.....))).))))))......)))........))))))))))...(((((.....)))))............((((((((.....(((((((..................)))))))......)))))))).......))))))))))))))))))))))))..))).....))).).

***************************************************************************************************************************************************************************************************************************************************************************************************************************ATACGACTAGCGCGACTCGA********** Mar-F-2-m0064-3p 8

---------------------------------------------------------------------------------------------------------------------------------------------------------------------------------------------------------------------------------------------------------------------------------------------------------------------------ATACGACTAGCGCGACTC------------ t4688262 1

---------------------------------------------------------------------------------------------------------------------------------------------------------------------------------------------------------------------------------------------------------------------------------------------------------------------------ATACGACTAGCGCGACTCG----------- t0681752 3

---------------------------------------------------------------------------------------------------------------------------------------------------------------------------------------------------------------------------------------------------------------------------------------------------------------------------ATACGACTAGCGCGACTCGA---------- t0590696 3

---------------------------------------------------------------------------------------------------------------------------------------------------------------------------------------------------------------------------------------------------------------------------------------------------------------------------ATACGACTAGCGCGACTCGAATTC------ t8435438 1

//

mireap-plant

Mar-F-2-m0065 gi|73847232|gb|DT456252.1|DT456252:431:528:+ 98(nt) -25.40(kcal/mol)

CTCTTATACGAAGAGTCAGATTGCATTTTGCCATCTTTATTCAATAAATGGATAAATTATTCTTTATACAATATGCAATTTGACTCCTAGTACAAAAG Mar-F-2-m0065 11

......(((...((((((((((((((.(((.....((((((((.....)))))))).((.....))..))).))))))))))))))...)))......

**********AAGAGTCAGATTGCATTTTGC******************************************************************* Mar-F-2-m0065-5p 10

--------CGAAGAGTCAGATTGCATTTTGCC------------------------------------------------------------------ t8511233 1

---------GAAGAGTCAGATTGCATTTTGC------------------------------------------------------------------- t4027055 1

---------GAAGAGTCAGATTGCATTTTGCC------------------------------------------------------------------ t5484268 1

----------AAGAGTCAGATTGCATTTTG-------------------------------------------------------------------- t0665613 3

----------AAGAGTCAGATTGCATTTTGC------------------------------------------------------------------- t0480173 3

----------AAGAGTCAGATTGCATTTTGCC------------------------------------------------------------------ t2762108 1

--------------------------------------------------------------------------------TGACTCCTAGTACAAAAG t9018723 1

//

Mar-F-2-m0067 gi|73850009|gb|DT459029.1|DT459029:257:462:- 206(nt) -47.80(kcal/mol)

TTCTGTACGTGTGTACAGATATTGTATGCGTAGAGTATATTTTGTGTGCGTAGTGTGTGTAATCTCCTCCCTTTACAAAGATTAAAAGTTGGGCTTTTATAATCGAAAAAGGAAATCAACCGAAAATATACAATATATTTGTTTCCATTCGCTGCTGTTTTTGTTTCTTTCTTGCAGGTACAGAGTCTGTTGGCATGCGCACGGGG Mar-F-2-m0067 11

.(((((.(((((((((((((.(((((((((.(((((((((...))))))((((((.(((..........(((((.....(((((((((.....))))..)))))...)))))...........(((((((...))))))).....))).))))))..............)))))))..))))).))))))..))))))).))))).

*******************************************************************************************************************************************************************************AGGTACAGAGTCTGTTGGCAT********** Mar-F-2-m0067-3p 11

----------------------------------------------------------------------------------------------------------------------------------------------------------------------------TGCAGGTACAGAGTCTGTTGGCAT---------- t0872752 2

-----------------------------------------------------------------------------------------------------------------------------------------------------------------------------GCAGGTACAGAGTCTGTTGGCA----------- t9146208 1

-------------------------------------------------------------------------------------------------------------------------------------------------------------------------------AGGTACAGAGTCTGTTGG------------- t5475430 1

-------------------------------------------------------------------------------------------------------------------------------------------------------------------------------AGGTACAGAGTCTGTTGGCA----------- t1984651 1

-------------------------------------------------------------------------------------------------------------------------------------------------------------------------------AGGTACAGAGTCTGTTGGCAT---------- t0361945 4

-------------------------------------------------------------------------------------------------------------------------------------------------------------------------------AGGTACAGAGTCTGTTGGCATG--------- t0780107 2

//

Mar-F-2-m0069 gi|73943722|gb|DT527030.1|DT527030:53:167:+ 115(nt) -52.80(kcal/mol)

AAGCTCGTGGGATGGGTGAGGGGGTAAGACAATGGACTGTTTCAGTCTTTTGGAAACAGAAATAAAGAAGAAGGCTGGTTTATGTCTTGCCTACTCCACCCATGCCACTGGTTTC Mar-F-2-m0069 309

(((((.((((.(((((((((..(((((((((.(((((.....((((((((((....))...........))))))))))))))))))))))..)).))))))).)))).))))).

**********GATGGGTGAGGGGGTAAGACA************************************************************************************ Mar-F-2-m0069-5p 294

************************************************************************************TCTTGCCTACTCCACCCATGCC********* Mar-F-2-m0069-3p 2

AAGCTCGTGGGATGGGTGAGG---------------------------------------------------------------------------------------------- t8629040 1

-AGCTCGTGGGATGGGTGAG----------------------------------------------------------------------------------------------- t0681030 3

-AGCTCGTGGGATGGGTGAGG---------------------------------------------------------------------------------------------- t7473550 1

-AGCTCGTGGGATGGGTGAGGGGG------------------------------------------------------------------------------------------- t1039792 2

------GTGGGATGGGTGAGGGGGT------------------------------------------------------------------------------------------ t7039138 1

------GTGGGATGGGTGAGGGGGTA----------------------------------------------------------------------------------------- t1317089 2

------GTGGGATGGGTGAGGGGGTAA---------------------------------------------------------------------------------------- t6503587 1

-------TGGGATGGGTGAGGGGGTAAG--------------------------------------------------------------------------------------- t0161156 7

-------TGGGATGGGTGAGGGGGTAAGA-------------------------------------------------------------------------------------- t7539342 1

-------TGGGATGGGTGAGGGGGTAAGACA------------------------------------------------------------------------------------ t0057066 15

--------GGGATGGGTGAGGGGGTAAG--------------------------------------------------------------------------------------- t1333073 2

--------GGGATGGGTGAGGGGGTAAGA-------------------------------------------------------------------------------------- t0047606 18

--------GGGATGGGTGAGGGGGTAAGAC------------------------------------------------------------------------------------- t4033952 1

--------GGGATGGGTGAGGGGGTAAGACA------------------------------------------------------------------------------------ t3963238 1

---------GGATGGGTGAGGGGGTAAGAC------------------------------------------------------------------------------------- t0015710 40

---------GGATGGGTGAGGGGGTAAGACA------------------------------------------------------------------------------------ t4308041 1

---------GGATGGGTGAGGGGGTAAGACAA----------------------------------------------------------------------------------- t2851674 1

----------GATGGGTGAGGGGGTAAGAC------------------------------------------------------------------------------------- t0636404 3

----------GATGGGTGAGGGGGTAAGACA------------------------------------------------------------------------------------ t0002784 127

----------GATGGGTGAGGGGGTAAGACAA----------------------------------------------------------------------------------- t0047480 18

----------GATGGGTGAGGGGGTAAGACAATG--------------------------------------------------------------------------------- t0458517 3

-----------ATGGGTGAGGGGGTAAGACAA----------------------------------------------------------------------------------- t1580422 1

------------TGGGTGAGGGGGTAAGACAA----------------------------------------------------------------------------------- t0029143 25

------------TGGGTGAGGGGGTAAGACAAT---------------------------------------------------------------------------------- t0024673 29

------------TGGGTGAGGGGGTAAGACAATG--------------------------------------------------------------------------------- t2528930 1

--------------------------------------------------------------------------------TATGTCTTGCCTACTCCACCCA------------- t8125476 1

---------------------------------------------------------------------------------ATGTCTTGCCTACTCCACCCATGCC--------- t1308267 2

----------------------------------------------------------------------------------------------TCCACCCATGCCACTGGTTTC t3868948 1

//

Mar-F-2-m0071 gi|78335384|gb|DT555658.1|DT555658:1:123:+ 123(nt) -30.62(kcal/mol)

AAAGAAAGAGAGAAAGAGAGGCCTGGAAAAAGACCGAATTGACCTTATCTTTCTTTCGAAATTACCCTTTCTGCTTTATGTTCGACTCTTCGTAATTGCCATCCTCTCTCTCTCTCTCTCTCT Mar-F-2-m0071 8

..(((.(((((((.(((((((..(((...((...((((..((.....))......(((((......(.....).......)))))...))))...)).))).))))))).))))))).)))..

*****AAGAGAGAAAGAGAGGCCTGGA************************************************************************************************ Mar-F-2-m0071-5p 8

-----AAGAGAGAAAGAGAGGCCTG-------------------------------------------------------------------------------------------------- t3394858 1

-----AAGAGAGAAAGAGAGGCCTGG------------------------------------------------------------------------------------------------- t1329114 2

-----AAGAGAGAAAGAGAGGCCTGGA------------------------------------------------------------------------------------------------ t0308173 5

//

mireap-plant

Mar-F-3-m0001 gi|109827046|gb|DW225753.1|DW225753:109:383:+ 275(nt) -70.85(kcal/mol)

TGTCTTCTCCACTAAAAAATGGGCAAATTAGTCATTTTATATTAGATCACTGAGCAAAATGGTTTTTTCTTTTAAAAATTTCATCCCTCTGTACTATTAAAAGCTGGCGTGCTTAATAGAATAACTAGATAGTGACAATTGCATGTATCTCTTGCTGATGTGCATGGATTGGTTTTTAAGAGTAAAAATGAATAAAATTTTTAATAAAAGGATCATTTTGTTATTTGAGCTAATACACAGGGACTAATTTACTTAAATTTTTAGTATAGGGTCAA Mar-F-3-m0001 17

((.((.((..((((((((.((((.(((((((((......((((((.(((...((((((((((((((((...((((((((((..........((((.((((((((..(.(((((...(((.....)))...))).)).....(((((((.((.....)).)))))))..)..)))))))).))))..........))))))))))..))))))))))))))))...))).))))))......))))))))).))))..))))))))..)))).)).

**********ACTAAAAAATGGGCAAATTAG**************************************************************************************************************************************************************************************************************************************************** Mar-F-3-m0001-5p 16

----------ACTAAAAAATGGGCAAATTA----------------------------------------------------------------------------------------------------------------------------------------------------------------------------------------------------------------------------------------------------- t3913129 1

----------ACTAAAAAATGGGCAAATTAG---------------------------------------------------------------------------------------------------------------------------------------------------------------------------------------------------------------------------------------------------- t0081158 10

----------ACTAAAAAATGGGCAAATTAGT--------------------------------------------------------------------------------------------------------------------------------------------------------------------------------------------------------------------------------------------------- t4385229 1

-----------CTAAAAAATGGGCAAATTAG---------------------------------------------------------------------------------------------------------------------------------------------------------------------------------------------------------------------------------------------------- t1103589 2

------------TAAAAAATGGGCAAATTAGT--------------------------------------------------------------------------------------------------------------------------------------------------------------------------------------------------------------------------------------------------- t6399738 1

-------------AAAAAATGGGCAAATTAGT--------------------------------------------------------------------------------------------------------------------------------------------------------------------------------------------------------------------------------------------------- t1484846 1

----------------------------------------------------------------------------------------------------------------------------------------------------------------------------------------------------------------AGGATCATTTTGTTATTTGAGCTA------------------------------------------- t3772549 1

//

mireap-plant

Mar-F-3-m0002 gi|109835838|gb|DW234401.1|DW234401:60:372:- 313(nt) -61.05(kcal/mol)

CAATAGAAGTCTTTGGAGGGGAGATTAGAGCGGGAAAGAGTCAACAATGAAAAACTAACAATTTACAGTGGTAGCAGAAGAAAATAGCAGAACACGTAACAGGCATCAAAAGAGTTATTCTTAGCTCACTGATTCCTCGGATCGTTTATAATCCCCAGGCATTAAGAACACAAAACTCATCATCAGCTGATCACAGATTATTTACATACATATTTATAAAAATATACCCCACCATCATCGTGGTAGCTAAAACCAATCAATAATCCGGATGACGGTAATCACTATAATCATCCCTGTCCCTAGATGCGACGTC Mar-F-3-m0002 12

........((((..(((((((((((((.((.((......((((...................((((.(((...((...........))....))))))).(((.((((...((((((....))))))..)))).)))(((((.((((.((((((...)).)))).))))...............((((.(((((.(((...........((((((....))))))...........))))))))))))..............)))))..)))).....)).)).))))).))))).)))..))))........

**********CTTTGGAGGGGAGATTAGAGC****************************************************************************************************************************************************************************************************************************************************************************************** Mar-F-3-m0002-5p 12

---------TCTTTGGAGGGGAGATTAGAGC------------------------------------------------------------------------------------------------------------------------------------------------------------------------------------------------------------------------------------------------------------------------------------------ t3674485 1

----------CTTTGGAGGGGAGATTAGA-------------------------------------------------------------------------------------------------------------------------------------------------------------------------------------------------------------------------------------------------------------------------------------------- t0693936 2

----------CTTTGGAGGGGAGATTAGAG------------------------------------------------------------------------------------------------------------------------------------------------------------------------------------------------------------------------------------------------------------------------------------------- t2726139 1

----------CTTTGGAGGGGAGATTAGAGC------------------------------------------------------------------------------------------------------------------------------------------------------------------------------------------------------------------------------------------------------------------------------------------ t0186801 5

-----------TTTGGAGGGGAGATTAGAGC------------------------------------------------------------------------------------------------------------------------------------------------------------------------------------------------------------------------------------------------------------------------------------------ t0578527 2

------------TTGGAGGGGAGATTAGAGC------------------------------------------------------------------------------------------------------------------------------------------------------------------------------------------------------------------------------------------------------------------------------------------ t3589132 1

//

Mar-F-3-m0006 gi|109847551|gb|DW476531.1|DW476531:412:732:- 321(nt) -77.80(kcal/mol)

GACGAGACAAAGGGAAGGTTAGATATTTATAAAATGGCTATATTGAATATAAAAAAAATTTAATTCGTAAATTCCGGTAATGCCTCATACCCTATTCCGGCAATAAATACGGGTAGGGGTATTACAAAAGGCTTACCTTTAATACTGATAGAATCCATAGCTAAAAAGAAGAGACAACGAGGGGGACGACACAGTTTGATATAGCTTTTGACATAAGAAACTCTAGATCGGCTTTGGGGTTGTGGTTCGGGATCAGATGGGTGATTTTAAAGCTTCAAAGACGATTCTTCATTATAACATCTCATCTCCGTTTGTAATAGA Mar-F-3-m0006 9

......(((((.(((.(((.((((..((((((...((((((((..(((.((((.....))))..((((...((((.((((((((((.(((((((((.........)))).))))))))))))))).(((((....)))))........................................)))).))))....)))..))))))))...................(((((.(((((((((.....((..((((((......))))))..))))))))))).))))).....)))))).)))).)))))).)))))......

**********AGGGAAGGTTAGATATTTATA************************************************************************************************************************************************************************************************************************************************************************************************** Mar-F-3-m0006-5p 8

----------AGGGAAGGTTAGATATTTAT--------------------------------------------------------------------------------------------------------------------------------------------------------------------------------------------------------------------------------------------------------------------------------------------------- t0805629 2

----------AGGGAAGGTTAGATATTTATA-------------------------------------------------------------------------------------------------------------------------------------------------------------------------------------------------------------------------------------------------------------------------------------------------- t0393899 3

----------AGGGAAGGTTAGATATTTATAAA------------------------------------------------------------------------------------------------------------------------------------------------------------------------------------------------------------------------------------------------------------------------------------------------ t4020484 1

----------AGGGAAGGTTAGATATTTATAAAA----------------------------------------------------------------------------------------------------------------------------------------------------------------------------------------------------------------------------------------------------------------------------------------------- t1046894 2

---------------------------------------------------------------------------GGTAATGCCTCATACCCTATTCC------------------------------------------------------------------------------------------------------------------------------------------------------------------------------------------------------------------------------- t2179485 1

//

Mar-F-3-m0008 gi|109849019|gb|DW477998.1|DW477998:430:549:+ 120(nt) -29.40(kcal/mol)

GTCTGTTATCGCAAGAGGAATCTGCAATATTCTTTGAGCAATGTCCATTTGGAAGGTTCAATCCCTGGTATCCATCGATCAATCCAAATAGAGATTGCATTTCCTCTTCCAACTCTCCAG Mar-F-3-m0008 46

..........(.((((((((..((((((.((((((((((....(((....)))..))))))....(((.(((....)))....)))...)))))))))).)))))))).)..........

****************************************************************************************TAGAGATTGCATTTCCTCTTCC********** Mar-F-3-m0008-3p 46

----------------------------------------------------------------------------------------TAGAGATTGCATTTCCTCTT------------ t2476591 1

----------------------------------------------------------------------------------------TAGAGATTGCATTTCCTCTTC----------- t0086793 9

----------------------------------------------------------------------------------------TAGAGATTGCATTTCCTCTTCC---------- t0047770 14

----------------------------------------------------------------------------------------TAGAGATTGCATTTCCTCTTCCA--------- t3617633 1

-----------------------------------------------------------------------------------------AGAGATTGCATTTCCTCT------------- t3429828 1

-----------------------------------------------------------------------------------------AGAGATTGCATTTCCTCTTCC---------- t0085047 9

-----------------------------------------------------------------------------------------AGAGATTGCATTTCCTCTTCCA--------- t0070951 11

//

Mar-F-3-m0014 gi|109861703|gb|DW490680.1|DW490680:244:345:- 102(nt) -23.40(kcal/mol)

TGGAATCGTCTTAGGTCTAATTCCTATTACTTTGGCTGGATTATTCGTAACCGCATATTTACAATACAGACGTGGTGATCAGTTGGACCTTTGATTAATTAA Mar-F-3-m0014 11

.......(((..((((((((((...((((((.((.(((...((((.((((........))))))))))).)).)))))).))))))))))..))).......

**********************************************************************CGTGGTGATCAGTTGGACCTTT********** Mar-F-3-m0014-3p 10

-----------------------------------------------------------------ACAGACGTGGTGATCAGTTGG---------------- t4720919 1

-------------------------------------------------------------------AGACGTGGTGATCAGTTGGACCTT----------- t6304484 1

----------------------------------------------------------------------CGTGGTGATCAGTTGGACCTTT---------- t0391979 3

----------------------------------------------------------------------CGTGGTGATCAGTTGGACCTTTG--------- t1110568 2

-----------------------------------------------------------------------GTGGTGATCAGTTGGACCTTT---------- t6209833 1

-----------------------------------------------------------------------GTGGTGATCAGTTGGACCTTTG--------- t0681445 2

------------------------------------------------------------------------TGGTGATCAGTTGGACCTTTG--------- t6834390 1

//

Mar-F-3-m0018 gi|109864996|gb|DW493972.1|DW493972:308:408:- 101(nt) -28.70(kcal/mol)

TTATTATTGAGGCAGCGGTTCATCGATCTCTTCCTGAGAATTTTGTTGTGAGAATAACACAAATACATGAATCGATCGATAAACCTCTGCATCCAGCGCCT Mar-F-3-m0018 6

........((.((((.((((.(((((((..(((.((...((((((((((....)))))).)))).)).)))..))))))).)))).)))).))........

**********GGCAGCGGTTCATCGATCTCT********************************************************************** Mar-F-3-m0018-5p 6

---------AGGCAGCGGTTCATCGATCTCT---------------------------------------------------------------------- t4283304 1

----------GGCAGCGGTTCATCGATCTCT---------------------------------------------------------------------- t0233752 4

----------GGCAGCGGTTCATCGATCTCTT--------------------------------------------------------------------- t4858765 1

//

Mar-F-3-m0029 gi|116047768|gb|DS017321.1|:498963:499104:+ 142(nt) -46.64(kcal/mol)

TAATATATGGTTTAATTTCCTCCAATATCTTACTTTCAACGGTTCATGGTGAGCATTAACCAACGAAAAGATTGAGAAGAAGGGTGGCAACTGGCATGGATCTTGAACGTGTAAGAATTGGAGGGAAGTAAACCCTATAAGT Mar-F-3-m0029 5

...((((.((((((.(((((((((((.((((((.(((((.((((((((.(.((.....(((..(..............)...))).....)).))))))))))))))...))))))))))))))))).)))))).))))...

**********TTTAATTTCCTCCAATATCTTA************************************************************************************************************** Mar-F-3-m0029-5p 5

----------TTTAATTTCCTCCAATATCTT--------------------------------------------------------------------------------------------------------------- t4389419 1

----------TTTAATTTCCTCCAATATCTTA-------------------------------------------------------------------------------------------------------------- t0242146 4

//

Mar-F-3-m0031 gi|116047796|gb|DS017293.1|:187214:187315:- 102(nt) -53.30(kcal/mol)

AAGAATGGTCTGCCTGGCTCCCTGAATGCCATCTAGGAAGCTTGTCAAAGAGTGTTGGCAACCTCTCTAACTGGCATGAGGGGAGTCACGCAGGCCCAACTA Mar-F-3-m0031 23

......(((((((.(((((((((..((((((..(((..((.(((((((......))))))).))..)))..))))))..))))))))).)))))))......

**********TGCCTGGCTCCCTGAATGCCA*********************************************************************** Mar-F-3-m0031-5p 23

----------TGCCTGGCTCCCTGAATGCCA----------------------------------------------------------------------- t0025760 22

----------TGCCTGGCTCCCTGAATGCCAT---------------------------------------------------------------------- t5676142 1

//

mireap-plant

Mar-F-3-m0032 gi|116047810|gb|DS017279.1|:607787:607932:- 146(nt) -54.30(kcal/mol)

TCTTGTTTGATAGCCAAGGATGACTTGCCTGCTACATACAAGAGATCATTATGCATAAATAGAACGAAATATGTACATGGTATTAATTAATGCAGTGAAAACTCTTGTTTGGTTGCCAGGCAGTCTCCTTGGCTAAGCTGACAGGC Mar-F-3-m0032 10

.((((((...((((((((((.((((.((((((..((.(((((((.......((((((............))))))(((.(((((....))))).)))....))))))).))...).)))))))))))))))))))....)))))).

**********TAGCCAAGGATGACTTGCCTG******************************************************************************************************************* Mar-F-3-m0032-5p 8

**********************************************************************************************************************GGCAGTCTCCTTGGCTAAG********* Mar-F-3-m0032-3p 2

----------TAGCCAAGGATGACTTGCCTG------------------------------------------------------------------------------------------------------------------- t0115757 7

----------TAGCCAAGGATGACTTGCCTGC------------------------------------------------------------------------------------------------------------------ t2805052 1

--------------------------------------------------------------------------------------------------------------------CAGGCAGTCTCCTTGGCTA----------- t0847276 2

//

Mar-F-3-m0035 gi|116047817|gb|DS017272.1|:112745:112876:+ 132(nt) -44.99(kcal/mol)

AGAGTGACTATGATATTGGCCTGGTTCACTCAGATCACGACTTCAGAGCAAAGTGTCTTTCTTCTTCTTCTTCTTCTTCTTGTTTTATGTTCGTTTGATTTGATTGAGCCGTGCCAATATCTCAGTACTCTT Mar-F-3-m0035 18

((((((.((..(((((((((.(((((((.(((((((((((.(..(((((((............................)))))))..).))))..))))))).))))))).)))))))))..)))))))).

**********TGATATTGGCCTGGTTCACTC***************************************************************************************************** Mar-F-3-m0035-5p 18

----------TGATATTGGCCTGGTTCACTC----------------------------------------------------------------------------------------------------- t0046626 14

-------------TATTGGCCTGGTTCACTCAGA-------------------------------------------------------------------------------------------------- t0306719 4

//

mireap-plant

Mar-F-3-m0036 gi|116047820|gb|DS017269.1|:182564:182704:+ 141(nt) -42.60(kcal/mol)

CAACTCAGCATCTCTTGAGATTTTGAGAAGAGTTGCTCTTTGAAGAGAGCACAATACGACGAAAGTTGAAAGCTATGTTCGATGGGGGAGTTATTGTCTATTGTAAACCTCTATGGTAGAATCAGTCGGGGGTCTGAGAGG Mar-F-3-m0036 11

...(((((...((((((((((((((...((((.(((((((....))))))).....((((....))))...........(((((((.((....)).))))))).....))))....)))))))..))))))).)))))...

**************TTGAGATTTTGAGAAGAGTT*********************************************************************************************************** Mar-F-3-m0036-5p 1

************************************************************************************************************CTCTATGGTAGAATCAGTCGGGG********** Mar-F-3-m0036-3p 10

--------------TTGAGATTTTGAGAAGAGTTGC--------------------------------------------------------------------------------------------------------- t3561601 1

-----------------------------------------------------------------------------------------------------------CCTCTATGGTAGAATCAGTCGGGG---------- t1120249 2

------------------------------------------------------------------------------------------------------------CTCTATGGTAGAATCAGTC-------------- t5469030 1

------------------------------------------------------------------------------------------------------------CTCTATGGTAGAATCAGTCGG------------ t0883756 2

------------------------------------------------------------------------------------------------------------CTCTATGGTAGAATCAGTCGGGG---------- t0380340 3

------------------------------------------------------------------------------------------------------------CTCTATGGTAGAATCAGTCGGGGG--------- t1188515 1

--------------------------------------------------------------------------------------------------------------CTATGGTAGAATCAGTCGGGGG--------- t3299068 1

//

Mar-F-3-m0038 gi|116047824|gb|CM000354.1|:12256220:12256366:- 147(nt) -62.70(kcal/mol)

GGTCATGCTTTTCCACAGCTTTCTTGAACTTCTTTGCCTTGCTTAATCTGTGTATATATAGATCACTACATGTACAGCTCCTATATATATAAATATATGTATGTATAGCGCCATGGAAGCTCAAGAAAGCTGTGGGAGAACATGGCA Mar-F-3-m0038 462

.((((((.(((((((((((((((((((.(((((..((.............(((((((.(((....))).)))))))....(((((((((((......))))))))))).))...))))).))))))))))))))))))).)))))).

**********TTCCACAGCTTTCTTGAACTT******************************************************************************************************************** Mar-F-3-m0038-5p 458

--TCATGCTTTTCCACAGCTT------------------------------------------------------------------------------------------------------------------------------ t3055203 1

--TCATGCTTTTCCACAGCTTTC---------------------------------------------------------------------------------------------------------------------------- t1104748 2

---CATGCTTTTCCACAGCTTTCT--------------------------------------------------------------------------------------------------------------------------- t2175726 1

--------TTTTCCACAGCTTTCTTGAAC---------------------------------------------------------------------------------------------------------------------- t5856966 1

----------TTCCACAGCTTTCTTGAA----------------------------------------------------------------------------------------------------------------------- t0006931 58

----------TTCCACAGCTTTCTTGAAC---------------------------------------------------------------------------------------------------------------------- t0014626 34

----------TTCCACAGCTTTCTTGAACT--------------------------------------------------------------------------------------------------------------------- t0119483 7

----------TTCCACAGCTTTCTTGAACTT-------------------------------------------------------------------------------------------------------------------- t0000511 353

----------TTCCACAGCTTTCTTGAACTTC------------------------------------------------------------------------------------------------------------------- t2455448 1

-----------TCCACAGCTTTCTTGAACTT-------------------------------------------------------------------------------------------------------------------- t0443868 3

-------------CACAGCTTTCTTGAACTT-------------------------------------------------------------------------------------------------------------------- t5082590 1

//

Mar-F-3-m0041 gi|116047828|gb|CM000350.1|:3895079:3895218:+ 140(nt) -50.90(kcal/mol)

TTTTTTGAGGGGAATGTTGTCTGGCTCGAGGACTTTTTGTTCATCAATCTAATCGAACTTTCTACCTGTAGATCTAGTATCTTATTTAAGATTGATCACGTATTAGGGTTGTCGGACCAGGCTTCATTCCCCCCAATCAT Mar-F-3-m0041 908

....(((.((((((((..((((((..(((.((((((.(((..((((((((((..(((((.((((....))))...))).))....)).)))))))).)))....)))))).)))..))))))..)))))))).)))....

**********GGAATGTTGTCTGGCTCGAGG************************************************************************************************************* Mar-F-3-m0041-5p 908

--------GGGGAATGTTGTCTGGCTCGA--------------------------------------------------------------------------------------------------------------- t0157451 6

---------GGGAATGTTGTCTGGCTCGAG-------------------------------------------------------------------------------------------------------------- t0374756 3

---------GGGAATGTTGTCTGGCTCGAGG------------------------------------------------------------------------------------------------------------- t0459819 3

----------GGAATGTTGTCTGGCTCGAG-------------------------------------------------------------------------------------------------------------- t0198049 5

----------GGAATGTTGTCTGGCTCGAGG------------------------------------------------------------------------------------------------------------- t0000168 817

----------GGAATGTTGTCTGGCTCGAGGA------------------------------------------------------------------------------------------------------------ t0032334 19

----------GGAATGTTGTCTGGCTCGAGGAC----------------------------------------------------------------------------------------------------------- t0263031 4

-----------GAATGTTGTCTGGCTCGAGG------------------------------------------------------------------------------------------------------------- t0905280 2

-----------GAATGTTGTCTGGCTCGAGGA------------------------------------------------------------------------------------------------------------ t0009118 47

-----------GAATGTTGTCTGGCTCGAGGAC----------------------------------------------------------------------------------------------------------- t5553893 1

------------AATGTTGTCTGGCTCGAGG------------------------------------------------------------------------------------------------------------- t3800619 1

//

Mar-F-3-m0043 gi|116047829|gb|CM000349.1|:2740290:2740387:+ 98(nt) -45.64(kcal/mol)

CACCGCTATTAGTTGAAGCTGCCAACATGATCTGAGCTTTCCTTAATTTTCCTATACAGGAAAGACTAGATCATGTGGCAGTTTCACCTATTGATGGT Mar-F-3-m0043 35

.((((.((.(((.(((((((((((.(((((((((..(((((((..............)))))))..)))))))))))))))))))).))).)).))))

*******************************************************************AGATCATGTGGCAGTTTCACC********** Mar-F-3-m0043-3p 35

-------------------------------------------------------------------AGATCATGTGGCAGTTTCACC---------- t0021956 25

--------------------------------------------------------------------GATCATGTGGCAGTTTCACC---------- t0279810 4

---------------------------------------------------------------------ATCATGTGGCAGTTTCACC---------- t3119034 1

---------------------------------------------------------------------ATCATGTGGCAGTTTCACCT--------- t0178444 5

//

mireap-plant

Mar-F-3-m0044 gi|116047829|gb|CM000349.1|:12992294:12992489:+ 196(nt) -82.60(kcal/mol)

ATGGTTTAAGAGAGCTTTCTTCAGTCCACTCATGGACGGGCGAAGGGTTTGGATTAGCTGCCGACTCATTCATTCAAACACAGTAGAAATTAAGGGAGCAGTATGGCTGCTATTGTGAATGTGTGAATGATGCGGGAGATAAATTTCATCCTTTTCTTCTCTGTGCTTGGACTGAAGGGAGCTCCCTTTAATCGTT Mar-F-3-m0044 8

((((((.(((.(((((..((((((((((..((((((..((.(((((((..(((((..((.(((..((((((((.((..((((((............(((((.....)))))))))))..)).))))))))..))).))...))))).))))))).))..))))))..))))))))))..))))).))).)))))).

**********AGAGCTTTCTTCAGTCCACTC********************************************************************************************************************************************************************* Mar-F-3-m0044-5p 7

----------AGAGCTTTCTTCAGTCCACTC--------------------------------------------------------------------------------------------------------------------------------------------------------------------- t0139692 6

----------AGAGCTTTCTTCAGTCCACTCA-------------------------------------------------------------------------------------------------------------------------------------------------------------------- t2773811 1

-------------------------------------------------------AGCTGCCGACTCATTCATTCA------------------------------------------------------------------------------------------------------------------------ t4220451 1

//

Mar-F-3-m0048 gi|116047831|gb|CM000347.1|:286903:286992:- 90(nt) -50.80(kcal/mol)

TAACGAGATATTGGTGCGGTTCAATCAGATAGTAATGCTCCATAAGCATAGAGCTCTATTGTTTGATTGAGCCGTGCCAATATCACGTAT Mar-F-3-m0048 114

..(((.((((((((..((((((((((((((((((..((((.((....)).))))..))))))))))))))))))..)))))))).)))..

**********TTGGTGCGGTTCAATCAGATA*********************************************************** Mar-F-3-m0048-5p 111

-----AGATATTGGTGCGGTTCAATC---------------------------------------------------------------- t0351681 3

-------ATATTGGTGCGGTTCAATCAG-------------------------------------------------------------- t0059025 12

--------TATTGGTGCGGTTCAATCAGA------------------------------------------------------------- t4490651 1

---------ATTGGTGCGGTTCAATCAGA------------------------------------------------------------- t6667553 1

---------ATTGGTGCGGTTCAATCAGAT------------------------------------------------------------ t0015513 32

---------ATTGGTGCGGTTCAATCAGATA----------------------------------------------------------- t4847479 1

----------TTGGTGCGGTTCAATCAGA------------------------------------------------------------- t0959119 2

----------TTGGTGCGGTTCAATCAGAT------------------------------------------------------------ t0607916 2

----------TTGGTGCGGTTCAATCAGATA----------------------------------------------------------- t0006585 60

//

mireap-plant

Mar-F-3-m0049 gi|116047832|gb|CM000346.1|:8492280:8492363:+ 84(nt) -35.76(kcal/mol)

AGGGGAATGCTGTCTGATTCGAGACCATTCACTTTAAGCACACATTCATCTTTCGAATGATCTCGGACCAGGCTTCATTCCCCC Mar-F-3-m0049 20

.((((((((..(((((.(((((((.(((((........................))))).))))))).)))))..)))))))).

*****************************************************CGAATGATCTCGGACCAGGCT********** Mar-F-3-m0049-3p 20

----------------------------------------------------TCGAATGATCTCGGACCAGGC----------- t1975720 1

-----------------------------------------------------CGAATGATCTCGGACCAGGCT---------- t0034753 18

-----------------------------------------------------CGAATGATCTCGGACCAGGCTT--------- t6880796 1

//

mireap-plant

Mar-F-3-m0050 gi|116047832|gb|CM000346.1|:9042207:9042303:- 97(nt) -44.00(kcal/mol)

GCACCTCTATCAGTTGAAGCTGCCAGCATGATCTTAGCCTTCCTCCTTTGTTGAGGAAAGAAACAGATCATGTGGCAGTTTCACCTGTTGTTGGTAT Mar-F-3-m0050 154

..(((..((.(((.(((((((((((.((((((((...(.((((((.......)))))).)....))))))))))))))))))).))).))..)))..

******************************************************************ATCATGTGGCAGTTTCACCTG********** Mar-F-3-m0050-3p 154

---------------------------------------------------------------CAGATCATGTGGCAGTTTCA-------------- t3236073 1

---------------------------------------------------------------CAGATCATGTGGCAGTTTCACC------------ t0170431 6

----------------------------------------------------------------AGATCATGTGGCAGTTTCACC------------ t0021956 25

-----------------------------------------------------------------GATCATGTGGCAGTTTCACC------------ t0279810 4

------------------------------------------------------------------ATCATGTGGCAGTTTCACC------------ t3119034 1

------------------------------------------------------------------ATCATGTGGCAGTTTCACCT----------- t0178444 5

------------------------------------------------------------------ATCATGTGGCAGTTTCACCTG---------- t0002748 111

------------------------------------------------------------------ATCATGTGGCAGTTTCACCTGT--------- t2863956 1

//

Mar-F-3-m0056 gi|116047834|gb|CM000344.1|:6416279:6416563:- 285(nt) -48.30(kcal/mol)

TCTTTTTTCTTCTTGACCTTGTAAGACCTTTTCTTGACCTTGTAAGACCCTGTTCTTCGTTCTTCATTTTTGTTTTTTTAATTGTCATTTACTCTATATCAAAACTTTATGATATAGATTTTTGACAAAATCTTTTCCCATTTTCATCCAACTTATCTTCTCTTTTCTTATCTATCCCTCCTGAGCTATTTGAATTAGACTACGTATTTTCCTTCAAAATCACCCAATTAAAGACAAATCAACAACCTCTGAAAGGTCATGCAAGCGATCGAAGGAGCCTTATTT Mar-F-3-m0056 4475

........(((((((((((((((.((((((((...((..((((((((......))))...........(((((((((....((((((.....(((((((((........)))))))))....)))))).....................................................(((....((((((..(((........)))..)))))).))).......)))))))))...))))..)).)))))))).)))))).).)).))))))........

**********TCTTGACCTTGTAAGACCTTT************************************************************************************************************************************************************************************************************************************************************** Mar-F-3-m0056-5p 4369

-------TCTTCTTGACCTTGTAAGAC------------------------------------------------------------------------------------------------------------------------------------------------------------------------------------------------------------------------------------------------------------------ t4792472 1

-------TCTTCTTGACCTTGTAAGACC----------------------------------------------------------------------------------------------------------------------------------------------------------------------------------------------------------------------------------------------------------------- t0042058 15

-------TCTTCTTGACCTTGTAAGACCT---------------------------------------------------------------------------------------------------------------------------------------------------------------------------------------------------------------------------------------------------------------- t1124856 2

--------CTTCTTGACCTTGTAAGACCT---------------------------------------------------------------------------------------------------------------------------------------------------------------------------------------------------------------------------------------------------------------- t0058477 12

--------CTTCTTGACCTTGTAAGACCTT--------------------------------------------------------------------------------------------------------------------------------------------------------------------------------------------------------------------------------------------------------------- t0413094 3

---------TTCTTGACCTTGTAAGAC------------------------------------------------------------------------------------------------------------------------------------------------------------------------------------------------------------------------------------------------------------------ t1125504 2

---------TTCTTGACCTTGTAAGACC----------------------------------------------------------------------------------------------------------------------------------------------------------------------------------------------------------------------------------------------------------------- t0044472 15

---------TTCTTGACCTTGTAAGACCT---------------------------------------------------------------------------------------------------------------------------------------------------------------------------------------------------------------------------------------------------------------- t0024276 23

---------TTCTTGACCTTGTAAGACCTT--------------------------------------------------------------------------------------------------------------------------------------------------------------------------------------------------------------------------------------------------------------- t0000140 947

---------TTCTTGACCTTGTAAGACCTTT-------------------------------------------------------------------------------------------------------------------------------------------------------------------------------------------------------------------------------------------------------------- t0011151 41

---------TTCTTGACCTTGTAAGACCTTTT------------------------------------------------------------------------------------------------------------------------------------------------------------------------------------------------------------------------------------------------------------- t3832526 1

---------TTCTTGACCTTGTAAGACCTTTTC------------------------------------------------------------------------------------------------------------------------------------------------------------------------------------------------------------------------------------------------------------ t3552067 1

----------TCTTGACCTTGTAAGACC----------------------------------------------------------------------------------------------------------------------------------------------------------------------------------------------------------------------------------------------------------------- t0178456 5

----------TCTTGACCTTGTAAGACCT---------------------------------------------------------------------------------------------------------------------------------------------------------------------------------------------------------------------------------------------------------------- t0033290 18

----------TCTTGACCTTGTAAGACCTT--------------------------------------------------------------------------------------------------------------------------------------------------------------------------------------------------------------------------------------------------------------- t0008920 48

----------TCTTGACCTTGTAAGACCTTT-------------------------------------------------------------------------------------------------------------------------------------------------------------------------------------------------------------------------------------------------------------- t0000046 2934

----------TCTTGACCTTGTAAGACCTTTT------------------------------------------------------------------------------------------------------------------------------------------------------------------------------------------------------------------------------------------------------------- t0172392 6

----------TCTTGACCTTGTAAGACCTTTTC------------------------------------------------------------------------------------------------------------------------------------------------------------------------------------------------------------------------------------------------------------ t0804343 2

----------TCTTGACCTTGTAAGACCTTTTCT----------------------------------------------------------------------------------------------------------------------------------------------------------------------------------------------------------------------------------------------------------- t4428350 1

-----------CTTGACCTTGTAAGACCTTT-------------------------------------------------------------------------------------------------------------------------------------------------------------------------------------------------------------------------------------------------------------- t0231078 4

-----------CTTGACCTTGTAAGACCTTTT------------------------------------------------------------------------------------------------------------------------------------------------------------------------------------------------------------------------------------------------------------- t0030620 20

-----------CTTGACCTTGTAAGACCTTTTC------------------------------------------------------------------------------------------------------------------------------------------------------------------------------------------------------------------------------------------------------------ t0974701 2

-----------CTTGACCTTGTAAGACCTTTTCT----------------------------------------------------------------------------------------------------------------------------------------------------------------------------------------------------------------------------------------------------------- t2410613 1

------------TTGACCTTGTAAGACCTTT-------------------------------------------------------------------------------------------------------------------------------------------------------------------------------------------------------------------------------------------------------------- t0531929 2

------------TTGACCTTGTAAGACCTTTT------------------------------------------------------------------------------------------------------------------------------------------------------------------------------------------------------------------------------------------------------------- t0286340 4

------------TTGACCTTGTAAGACCTTTTC------------------------------------------------------------------------------------------------------------------------------------------------------------------------------------------------------------------------------------------------------------ t0075858 10

------------TTGACCTTGTAAGACCTTTTCT----------------------------------------------------------------------------------------------------------------------------------------------------------------------------------------------------------------------------------------------------------- t2469398 1

-------------TGACCTTGTAAGACCTTTTC------------------------------------------------------------------------------------------------------------------------------------------------------------------------------------------------------------------------------------------------------------ t0046312 14

-------------TGACCTTGTAAGACCTTTTCT----------------------------------------------------------------------------------------------------------------------------------------------------------------------------------------------------------------------------------------------------------- t0001087 210

-------------TGACCTTGTAAGACCTTTTCTT---------------------------------------------------------------------------------------------------------------------------------------------------------------------------------------------------------------------------------------------------------- t0023544 24

------------------TTGTAAGACCTTTTCTTGA-------------------------------------------------------------------------------------------------------------------------------------------------------------------------------------------------------------------------------------------------------- t2494824 1

----------------------AAGACCTTTTCTTGACCTTGT-------------------------------------------------------------------------------------------------------------------------------------------------------------------------------------------------------------------------------------------------- t2943189 1

---------------------------CTTTTCTTGACCTTGTAAGAC--------------------------------------------------------------------------------------------------------------------------------------------------------------------------------------------------------------------------------------------- t0568706 2

----------------------------TTTTCTTGACCTTGTAAGACC-------------------------------------------------------------------------------------------------------------------------------------------------------------------------------------------------------------------------------------------- t0028355 21

-----------------------------TTTCTTGACCTTGTAAGACC-------------------------------------------------------------------------------------------------------------------------------------------------------------------------------------------------------------------------------------------- t3594003 1

-----------------------------TTTCTTGACCTTGTAAGACCC------------------------------------------------------------------------------------------------------------------------------------------------------------------------------------------------------------------------------------------- t0112112 8

------------------------------TTCTTGACCTTGTAAGAC--------------------------------------------------------------------------------------------------------------------------------------------------------------------------------------------------------------------------------------------- t1125504 2

------------------------------TTCTTGACCTTGTAAGACC-------------------------------------------------------------------------------------------------------------------------------------------------------------------------------------------------------------------------------------------- t0044472 15

------------------------------TTCTTGACCTTGTAAGACCC------------------------------------------------------------------------------------------------------------------------------------------------------------------------------------------------------------------------------------------- t0071224 11

------------------------------TTCTTGACCTTGTAAGACCCT------------------------------------------------------------------------------------------------------------------------------------------------------------------------------------------------------------------------------------------ t0012359 38

-------------------------------TCTTGACCTTGTAAGACC-------------------------------------------------------------------------------------------------------------------------------------------------------------------------------------------------------------------------------------------- t0178456 5

-------------------------------TCTTGACCTTGTAAGACCC------------------------------------------------------------------------------------------------------------------------------------------------------------------------------------------------------------------------------------------- t4432186 1

//

Mar-F-3-m0058 gi|116047835|gb|CM000343.1|:3395233:3395336:- 104(nt) -52.60(kcal/mol)

TGTGTTGAGGGGAATGTTGGCTGGCTCGAAGCTTAAGCAAAGAGTTTCCTAACATGAAACAACTGTTAAGGCTTCGGACCAGGCTTCATTCCCCTCAAACATAC Mar-F-3-m0058 10

((((((((((((((((..(.((((..((((((((.((((....(((((.......)))))...)))).))))))))..)))).)..)))))))))).)))))).

**********GGAATGTTGGCTGGCTCGAAG************************************************************************* Mar-F-3-m0058-5p 10

----------GGAATGTTGGCTGGCTCGAAG------------------------------------------------------------------------- t0081100 10

//

Mar-F-3-m0064 gi|116047837|gb|CM000341.1|:15276189:15276277:- 89(nt) -40.80(kcal/mol)

AGGGGATTGTCGTCTGGTTCGATGTCATTCATGAGAAGCTCAAACATAAACGTAATATTGAATGATTTCGGACCAGGCTTCATTCCCCC Mar-F-3-m0064 7

.(((((.((..(((((((((((.((((((((.(((...)))..((......)).....)))))))).)))))))))))..)).))))).

**********************************************************TGAATGATTTCGGACCAGGCT********** Mar-F-3-m0064-3p 7

---------------------------------------------------------TTGAATGATTTCGGACCAGGCT---------- t2425490 1

----------------------------------------------------------TGAATGATTTCGGACCAGGCT---------- t0142346 6

//

mireap-plant

Mar-F-3-m0065 gi|116047839|gb|CM000339.1|:2714458:2714726:+ 269(nt) -76.37(kcal/mol)

TATTCGTTGATGGTTGCCGGCTCACTGAACCGGGTCGGCAAGAGACACGTGTCGAAATCACGCTTGTCTGTACCCTCCATAAATAACCCTCATCCCACTCAAATCGAGTAGTCTATAACTAGGGTTGGTTTTTTGAAATAATAATAAATTCAGAAATAGCTAAGTGTATTTTTAGTCGTGTGTGAAAACCGATCAGAGAAGGAGAGACGATTTACTTACCGGCGGTCTCTGATTCGCTTGGTGCAGGTCGGGAACTGATTCGGCGATTT Mar-F-3-m0065 28

...(((((((.((((.(((((...(((.((((((.(((..((((((....((((.........((((((.....((((..................((((.....)))).........((..(((((((((((((((...........))))...(..((((((......))))))..)....)))))))))))..))..)))))))))).........)))).))))))...))))))))).)))))))).))))...)))))))...

**********************************************************************************************************************************************************************************************************************************************TGGTGCAGGTCGGGAACTGAT********** Mar-F-3-m0065-3p 28

----------------------------------------------------------------------------------------------------------------------------------------------------------------------------------------------------------------------------------------------TGGTGCAGGTCGGGAACTGA----------- t5697512 1

----------------------------------------------------------------------------------------------------------------------------------------------------------------------------------------------------------------------------------------------TGGTGCAGGTCGGGAACTGAT---------- t0019814 27

//

Mar-F-3-m0067 gi|116047839|gb|CM000339.1|:14406858:14406961:- 104(nt) -51.20(kcal/mol)

GGTCATGCTATTCCACGGCTTTCTTGAACTTCTTAAGTTATATGTATAGCTAGCAGCACCTCTAGGCTCTAGGAAGTTCAAGAAAGCTGTGGAAAAACATGGCA Mar-F-3-m0067 65

.((((((...(((((((((((((((((((((((((((((((....))))))...(((........))).)))))))))))))))))))))))))...)))))).

**********TTCCACGGCTTTCTTGAACTT************************************************************************* Mar-F-3-m0067-5p 65

----------TTCCACGGCTTTCTTGAA---------------------------------------------------------------------------- t0032879 19

----------TTCCACGGCTTTCTTGAAC--------------------------------------------------------------------------- t0312839 4

----------TTCCACGGCTTTCTTGAACT-------------------------------------------------------------------------- t0411484 3

----------TTCCACGGCTTTCTTGAACTT------------------------------------------------------------------------- t0011948 39

//

Mar-F-3-m0070 gi|116047841|gb|CM000337.1|:19074393:19074662:+ 270(nt) -69.07(kcal/mol)

TTTATGATTGTGCATTCTGATGTATGGGGACTGTCGAAAATAGCTACTCTTGGTGGTGCTCACTGGTTTGTTACTTTTATTGATGATTGTACCAGAATGACTTGGGTCGCTTTGTTGAAATCACAAAGCAAAGTCAGTTCAGCCTTTCAGAGGTTTCATAAAATGATTATGGTGCAATATAACAGCAAGATCTAAGTTCTTAGAAGTGACAGTGGTGGTGAATATGTTAATTTAGAACTTCGTACCTTCCTGGAACTGCATGGTATTGTT Mar-F-3-m0070 22

......((..((((((((((.((((((((.............(((((.....)))))..((((((.(((((((((((((..(((.(((((((((...((((((.....(((((((.(....)))))))).))))))....((((((...))))))..............))))))))).................)))..)))))))))))).).))))))................))))))))..))..)))).))))..))......

**********TGCATTCTGATGTATGGGGAC*********************************************************************************************************************************************************************************************************************************************** Mar-F-3-m0070-5p 22

--------TGTGCATTCTGATGTATGGGG------------------------------------------------------------------------------------------------------------------------------------------------------------------------------------------------------------------------------------------------- t4966268 1

----------TGCATTCTGATGTATGGGGAC----------------------------------------------------------------------------------------------------------------------------------------------------------------------------------------------------------------------------------------------- t0038569 17

----------TGCATTCTGATGTATGGGGACT---------------------------------------------------------------------------------------------------------------------------------------------------------------------------------------------------------------------------------------------- t6392964 1

------------CATTCTGATGTATGGGGA------------------------------------------------------------------------------------------------------------------------------------------------------------------------------------------------------------------------------------------------ t5173374 1

------------CATTCTGATGTATGGGGAC----------------------------------------------------------------------------------------------------------------------------------------------------------------------------------------------------------------------------------------------- t1049702 2

//

Mar-F-3-m0080 gi|123668821|emb|AM426479.1|:8261:8410:- 150(nt) -32.70(kcal/mol)

GTTTTAAAAAATAATTATACAAGCATATGATTAAAAATAAAACATTATGAGTTTGTTTAATTGTGCTATTTAAAAATAGTTTTTTGTTTTTCAAAACAGAAAACTGTTTTAAAWTTTTTTTAATATTGTTTGGATATTGTTTTTGAAAAA Mar-F-3-m0080 14

.((((.(((((((((....((((((....((((((((..(((((.........)))))..............(((((((((((((((((....)))))))))))))))))......))))))))..))))))...))))))))).)))).

**********************************************************************************************************************TTTAATATTGTTTGGATATTGT********** Mar-F-3-m0080-3p 14

--------------------------------------------------------------------------------------------------------------------TTTTTAATATTGTTTGGATATTGT---------- t5972115 1

----------------------------------------------------------------------------------------------------------------------TTTAATATTGTTTGGATATTG----------- t4783801 1

----------------------------------------------------------------------------------------------------------------------TTTAATATTGTTTGGATATTGT---------- t0103289 8

-----------------------------------------------------------------------------------------------------------------------TTAATATTGTTTGGATATTGT---------- t0255527 4

//

Mar-F-3-m0087 gi|147777644|emb|AM432580.2|:7828:7948:- 121(nt) -52.00(kcal/mol)

TCAGAAAGTATGGTGCAGCCAAGGATGACTTGCCGACTCTCTACTTAGCCATCTGCCTTTCTCCAAGGAGGCTTGCAGTACTGATGAAGCCGGCAAGTTGTCTTTGGCTACATGCTTCTTT Mar-F-3-m0087 5

..((((.(((((....(((((((((..((((((((.((.((...((((....((((...((((....))))...))))..)))).)))).))))))))..))))))))).)))))))))..

******************************************************************************************CGGCAAGTTGTCTTTGGCTAC********** Mar-F-3-m0087-3p 5

------------------------------------------------------------------------------------------CGGCAAGTTGTCTTTGGCTAC---------- t0204756 5

//

Mar-F-3-m0091 gi|147790249|emb|AM457961.2|:1659:1788:+ 130(nt) -63.81(kcal/mol)

TTGTTGTTTGCAGGTGTAGCATCATCAAGATTCACATGCAAAAGCCCGGCAGCGGCAGCGATGCCATCTTCGCTGCCTCAAGTTTTGGGGTTGAGAATCTTGATGATGCTGCATCGGCAATAGATGACTA Mar-F-3-m0091 14

..((..(((((.(((((((((((((((((((((.((.......((((.(.(((((((((((........))))))))....))).).)))))).))))))))))))))))))))).)))...))..))..

**********CAGGTGTAGCATCATCAAGAT*************************************************************************************************** Mar-F-3-m0091-5p 12

**************************************************************************************************CTTGATGATGCTGCATCGGCA*********** Mar-F-3-m0091-3p 1

----------CAGGTGTAGCATCATCAAGAT--------------------------------------------------------------------------------------------------- t0121838 7

-----------AGGTGTAGCATCATCAAGATT-------------------------------------------------------------------------------------------------- t0178141 5

---------------GTAGCATCATCAAGATTCACA---------------------------------------------------------------------------------------------- t5535403 1

--------------------------------------------------------------------------------------------------CTTGATGATGCTGCATCGGCA----------- t6605773 1

//

mireap-plant

Mar-F-3-m0092 gi|147798985|emb|AM466568.2|:13694:14042:+ 349(nt) -111.30(kcal/mol)

TTTGAGAGGTTGAAGCTGCCAGCATGATCTGGTGAAACAAACACCATCTCTTTCTTCTCTAACCCCATGTCTGGATTCGTCCACCGATCCATTATTATAGACCAGGCCGCCCGTTTCCCATGTAGTGATCGATAATTAGGCYSGGGGTTTTCACTTTTTAGTGGGATCTAATCCTTAGGATGGATGTTTGTATGGGTGGTATATATCATGGTGAGGTCTGTTTTCTATTTTAATTCTAACGGGGTTTTGATTTAGCTGAGGGGGTATAATTCATAGCCTAATTCCAAAACCTAACTCCATAGAGATAGGGTTCCATGATCAGGTCATCTTGCAGCTTCAATCACTCACT Mar-F-3-m0092 30

..((((.((((((((((((.((.((((((((((.(.......(((..(((...((..((.((((((.....((((((.(....).)))))).....(((((((..(((((((((.((((((..(((((.....((((........)))).))))).....))))))....((((.......))))......)))))))))...(((....))).)))))))...................))))))..))...))..)))..)))........................((((.(((....))).))))......).)))))))))))).)))))))))))).))))..

******************************************************************************************************************************************************************************************************************************************************************************************************************************TCAGGTCATCTTGCAGCTTCA********** Mar-F-3-m0092-3p 30

------------------------------------------------------------------------------------------------------------------------------------------------------------------------------------------------------------------------------------------------------------------------------------------------------------------------------TCAGGTCATCTTGCAGCTTCA---------- t0025674 22

------------------------------------------------------------------------------------------------------------------------------------------------------------------------------------------------------------------------------------------------------------------------------------------------------------------------------TCAGGTCATCTTGCAGCTTCAA--------- t0870731 2

--------------------------------------------------------------------------------------------------------------------------------------------------------------------------------------------------------------------------------------------------------------------------------------------------------------------------------AGGTCATCTTGCAGCTTCAA--------- t0264670 4

--------------------------------------------------------------------------------------------------------------------------------------------------------------------------------------------------------------------------------------------------------------------------------------------------------------------------------AGGTCATCTTGCAGCTTCAAT-------- t0955011 2

//

mireap-plant

Mar-F-3-m0093 gi|147803570|emb|AM484368.2|:7809:7951:- 143(nt) -61.50(kcal/mol)

GGGAGAATCTGTTAAGCTCAGGAGGGATAGCGCCATGAGCCATGATGAAAATGATTGTTGAGTTGAAAATTTTTTCTAGCTTGTTTTTTGGGTCATCATTTTGTGTGTGTGGCGCTATCTATCCTGAGTTTCACGGGWTCTTC Mar-F-3-m0093 53

.(((((.(((((.((((((((((.(((((((((((((.((.(((((((...(((....((((((((((....)))).))))))....)))..)))))))...))...))))))))))))).)))))))))).))))).)))))

****************************************************************************************************************CGCTATCTATCCTGAGTTTCA********** Mar-F-3-m0093-3p 53

----------------------------------------------------------------------------------------------------------------CGCTATCTATCCTGAGTTTC----------- t1066570 2

----------------------------------------------------------------------------------------------------------------CGCTATCTATCCTGAGTTTCA---------- t0008924 48

-----------------------------------------------------------------------------------------------------------------GCTATCTATCCTGAGTTTCA---------- t0421951 3

//

Mar-F-3-m0095 gi|147816046|emb|AM463946.2|:2277:2370:+ 94(nt) -24.80(kcal/mol)

TTCATTTCTTTTGAAGACCCATTTGCAACCAACAATCTTTTGATTTTTAGGTTTCTCAACTAGTTGCCAAGTGTGGTTCTTTTGAAGAGATTCA Mar-F-3-m0095 26

...((((((((.(((((((((((((((((....((((....))))....((((....)))).)))).)))))).)).))))).))))))))...

**********TTGAAGACCCATTTGCAACCAA************************************************************** Mar-F-3-m0095-5p 26

----------TTGAAGACCCATTTGCAACCA--------------------------------------------------------------- t0108445 8

----------TTGAAGACCCATTTGCAACCAA-------------------------------------------------------------- t0080521 10

----------TTGAAGACCCATTTGCAACCAAC------------------------------------------------------------- t0430651 3

-----------TGAAGACCCATTTGCAACCAA-------------------------------------------------------------- t0694003 2

-----------TGAAGACCCATTTGCAACCAAC------------------------------------------------------------- t0460374 3

//

Mar-F-3-m0110 gi|164257196|gb|ES791882.1|ES791882:531:614:+ 84(nt) -22.10(kcal/mol)

GGAATGAAACCTCTTGTTGGGCAAATGAGCATTTTTCCTCCTTGATGAACAGCTCATTTTCCCTTAAAGTTTGGAGCACCTCCC Mar-F-3-m0110 15

(((.((...((.(((...(((.((((((((.(((.((......)).)))..)))))))).)))...)))...))..))..))).

**********CTCTTGTTGGGCAAATGAGCAT**************************************************** Mar-F-3-m0110-5p 15

--------ACCTCTTGTTGGGCAAATGAGC------------------------------------------------------ t6386102 1

---------CCTCTTGTTGGGCAAATGAGCATT--------------------------------------------------- t5023742 1

----------CTCTTGTTGGGCAAATGAGCAT---------------------------------------------------- t0164409 6

----------CTCTTGTTGGGCAAATGAGCATT--------------------------------------------------- t0805253 2

-----------TCTTGTTGGGCAAATGAGCAT---------------------------------------------------- t0782158 2

-----------TCTTGTTGGGCAAATGAGCATT--------------------------------------------------- t5202623 1

------------CTTGTTGGGCAAATGAGCATT--------------------------------------------------- t0864384 2

//

Mar-F-3-m0113 gi|164301684|gb|ES798932.1|ES798932:588:798:+ 211(nt) -50.40(kcal/mol)

TGATGGGTTATGGGAACTTGAAGATGAGGCTGCTTATTACTATACTTTGTAACAGTCTGAACTCTTCATGTAGCTTCATGGCCGGAGAAACTACGAATCAAAACGAAATGGTAGGGGTTAAATTACACAATGGGAAAAAAAGGCATTGGGGGGGGAAATTTGAAAATGAAGGCCATTATCTTTTTGGGGACTATTCCTTTTGGACCCCCCA Mar-F-3-m0113 6

.....(((((((((.((.(((((((.((((((....((((........)))))))))).)..)))))).))...))))))))).....(((..(..((((........)))).)..)))........(((((...........))))).((((((......((.((((.....)))).)).....(((((....))))).....)))))).

**********TGGGAACTTGAAGATGAGGCT************************************************************************************************************************************************************************************ Mar-F-3-m0113-5p 6

----------TGGGAACTTGAAGATGAGGCT------------------------------------------------------------------------------------------------------------------------------------------------------------------------------------ t0141758 6

//

Mar-F-3-m0115 gi|164317397|gb|ES829294.1|ES829294:639:817:- 179(nt) -56.90(kcal/mol)

TGCTGTGATGCAGTAGGTGCTTGTTGTGGTTCTCCCAAAGAAACAAGGGCCAGTGAATTATCTGCCTTAGGTGAATTGAAGTCATCACCACTAAGCAAGTCCATTTTGGGATTGGCAGCTGGTGGAGGAGTTGAACCATTAGTTGCAGGAGGAGCAGGAAGCAGTAACTGATTAAATGG Mar-F-3-m0115 16

.....((((.((((.(.(((((.((((..(((((((((((.....(((((.((........))))))).(((((..((....)))))))..............)))))))....(((((((((((..........))))))))))).))))..)))).))))).).)))))))).....

**************************************************************************************************************************************************AGGAGGAGCAGGAAGCAGTAACT********** Mar-F-3-m0115-3p 15

--------------------------------------------------------------------------------------------------------------------------------------------AGTTGCAGGAGGAGCAGGAAGCAG--------------- t5868326 1

------------------------------------------------------------------------------------------------------------------------------------------------GCAGGAGGAGCAGGAAGCAGTAAC----------- t1780076 1

--------------------------------------------------------------------------------------------------------------------------------------------------AGGAGGAGCAGGAAGCAGTAACT---------- t0070949 11

--------------------------------------------------------------------------------------------------------------------------------------------------AGGAGGAGCAGGAAGCAGTAACTG--------- t2731489 1

---------------------------------------------------------------------------------------------------------------------------------------------------GGAGGAGCAGGAAGCAGTAACTGA-------- t6261947 1

-----------------------------------------------------------------------------------------------------------------------------------------------------AGGAGCAGGAAGCAGTAACTGAT------- t3067502 1

//

Mar-F-3-m0120 gi|164362130|gb|ES804885.1|ES804885:136:461:+ 326(nt) -102.07(kcal/mol)

CTCTGGTTTTAGTCCCTCTTCTATGCTGAAATTTATGATTTAATTCTTATTGTGATTTAGCAAATTTGATTGTCATTATTAGTAGGTCGGATTTGATAGCATCGTTATTAACGGGTTACTGCGAAAGTGATGAATTGATAATATGAATTTTTGCAGTAATGGAATTACTTATCAACAAAAAAAAAATCTATATCAACAGTGGTTAACAGAATTATCAAATTGGACTTACTAACAATACTCTAAAATAAAAGAATCATATTATGCAAAAATTAAATATTTGTGATTAAATCTCAAATTTCAACATAGTAGAGGGACTGAAACCGGAA Mar-F-3-m0120 7

.((((((((((((((((((.(((((.((((((((..(((((((((.....((((((((.....((((....((.(((.((((((((((.((((((((((..((((((...((..((((((((((((((..................)))))))))))))).((........)).........................))...)))).)).)))))))))).)))))))))).)))))....))))....))))))))....(((((.........))))))))))))))..)))))))).))))).)))))))))))))))))).

*******************************************************************************************************************************************************************************************************************************************************************************************************TTTCAACATAGTAGAGGGACT********** Mar-F-3-m0120-3p 6

-------------------------------------------------------------------------------------------------------------------------------------------------------------------------------------------------CAACAGTGGTTAACAGAATTAT--------------------------------------------------------------------------------------------------------------- t5376089 1

------------------------------------------------------------------------------------------------------------------------------------------------------------------------------------------------------------------------------------------------------------------------------------------------------ATTTCAACATAGTAGAGGGAC----------- t4719199 1

-------------------------------------------------------------------------------------------------------------------------------------------------------------------------------------------------------------------------------------------------------------------------------------------------------TTTCAACATAGTAGAGGGACT---------- t0215820 5

//

Mar-F-3-m0124 gi|189327957|gb|EQ138041.1|:913:1239:- 327(nt) -78.30(kcal/mol)

CATAAGTCTTTATATGGCTTAAAACAGGCTCCACGGGCATGGTACACTCGTCTGAGTGATTTTCTGATTTCTATTAGCTTTACTGCATCCAAGGTTGACACCTTCTTATTTATCTTATTTATTGGTGATAATATTTTTTATCTTCTGGTTTATGTTGATGACATTCTACTTACGGGTAGCAACTCTACTATGCTTCATCGGCTAATCCAGTTACTGAGTTCAGAGTTCAAGCTTCGTGACTTAGGTGTCGTTCACTATTTTTTAGGAATTAAGGTTCAACCCACTGCTATGGGTCTGATGTTACGCCAACATAAATATATTCTTAAC Mar-F-3-m0124 11

..((((..(((((.((((.(((..(((((.(((..((((.((..(((((....)))))..................((......))..))..((((((.(((((..(.....(((((.....((((((................((((.(((.(((((((((((.(((((....)))))..........)))..))))))))))).))))...((((((((..(((....)))..).))))))).......)))))).....))))))..)))))))))))...)))).))))))))...))).))))..)))))......))))..

**********TATATGGCTTAAAACAGGCTCC******************************************************************************************************************************************************************************************************************************************************************************************************* Mar-F-3-m0124-5p 11

----------TATATGGCTTAAAACAGGCTCC------------------------------------------------------------------------------------------------------------------------------------------------------------------------------------------------------------------------------------------------------------------------------------------------------- t0227193 5

-----------ATATGGCTTAAAACAGGCTCC------------------------------------------------------------------------------------------------------------------------------------------------------------------------------------------------------------------------------------------------------------------------------------------------------- t1080422 2

-----------ATATGGCTTAAAACAGGCTCCA------------------------------------------------------------------------------------------------------------------------------------------------------------------------------------------------------------------------------------------------------------------------------------------------------ t0295344 4

//

Mar-F-3-m0127 gi|270226120|emb|FN594950.1|:7578813:7578933:- 121(nt) -55.10(kcal/mol)

TGAGAATGTTTGGGAATTGGAGAGTAGGAAAGCTTAGCCATCTATTCCCTTCATGGTTTCCTCTCCATGGATTGGGGGTCTAGAGCTAGTCTTTCCTACTCCTCCCATTCCTATTGTTTTC Mar-F-3-m0127 21

.(((((((..(((((((.((((((((((((((.(((((..((((..((((((((((........))))))....))))..))))))))).)))))))))).)))).))))))).)))))))

*****************************************************************************************TCTTTCCTACTCCTCCCATTCC********** Mar-F-3-m0127-3p 21

-----------------------------------------------------------------------------------------TCTTTCCTACTCCTCCCATTC----------- t6769155 1

-----------------------------------------------------------------------------------------TCTTTCCTACTCCTCCCATTCC---------- t0032395 19

-------------------------------------------------------------------------------------------TTTCCTACTCCTCCCATTCC---------- t3861560 1

//

Mar-F-3-m0141 gi|270243647|emb|FN595992.1|:8843239:8843471:+ 233(nt) -50.46(kcal/mol)

TGTTAATGTACTGTACTCTTTTTTCCTTTGTCCAGGTTTTGTCCTACTAGTTTTACTGGCAAGGTTTTTAACGAGGTAGTCCTAACATACCAAGAACAACTTAAAGAATTATAAGAATATTGTACTCTTTTTCCTTTGCTAGGGTTTTTCCATAGGGTTTTTTATTAGCAAAGTTTTAATGAGGCATATTCTTCAATGTGATGGACATCCAAGGGGGAGTGTTATAATTGAAG Mar-F-3-m0141 10

..(((((......(((((((((((....((((((.(((((..(((.((((.....))))..)))..........((((((....)).)))).)))))...............((((((((.((.(((......(((((((((.......((....)).......))))))))).......)))))))))))))........))))))...))))))))))).....)))))..

********************************************************************************************************************************************************************************************************ATGGACATCCAAGGGGGAGTGTT********** Mar-F-3-m0141-3p 10

--------------------------------------------------------------------------------------------------------------------------------------------------------------------------------------------------------ATGGACATCCAAGGGGGAGTGTT---------- t0270158 4

--------------------------------------------------------------------------------------------------------------------------------------------------------------------------------------------------------ATGGACATCCAAGGGGGAGTGTTA--------- t0308959 4

---------------------------------------------------------------------------------------------------------------------------------------------------------------------------------------------------------TGGACATCCAAGGGGGAGTGTTAT-------- t0924910 2

//

Mar-F-3-m0146 gi|270244842|emb|FN596005.1|:479942:480175:- 234(nt) -75.70(kcal/mol)

GACAATGCGATCCCTTTGGATGTCTTCTTGCTCAGTGAGTTTTGGAGCATGTAAGCTGTTAGGTCAGAAGATGCCCAGACTCAATGTTGAGGTTATGGATGAAAGGGGGCGACCAGATTCAAGGCCAGAAAGCTGTTCAGTGGAGAAGCTTTACATATATAGATCAGTTGCTGGGCCAAGGAGTGACATGCCTCGATTTGTGTGGACAATGAAGACTCCGAGTTGAGGCTGTCT Mar-F-3-m0146 15

((((..((..((..((((((.(((((((((..((.(((((((.((.((((.(...(((......))).).)))))))))))))..((((((((.(((..((((...((....))...)))).((((....(((((.((.((((((...)))))).......)).)))))....)))).........))))))))))).....))..))).))))))))))))..)).)))))).

**********TCCCTTTGGATGTCTTCTTGC*********************************************************************************************************************************************************************************************************** Mar-F-3-m0146-5p 15

----------TCCCTTTGGATGTCTTCTTGC----------------------------------------------------------------------------------------------------------------------------------------------------------------------------------------------------------- t0043581 15

//

Mar-F-3-m0164 gi|270307419|emb|FN597030.1|:16730153:16730232:+ 80(nt) -23.40(kcal/mol)

CAACTTTTTGTTGCATCTTGTTTTTCTAGTAGCAGTTCAAGTGATGACTGGCTTCTAGACAGTGGATGCACAAAACACAT Mar-F-3-m0164 22

.....((((((.((((((...((.(((((.(((((((........)))).))).))))).)).)))))))))))).....

************************************************TGGCTTCTAGACAGTGGATGCA********** Mar-F-3-m0164-3p 22

------------------------------------------------TGGCTTCTAGACAGTGGATGC----------- t0504034 3

------------------------------------------------TGGCTTCTAGACAGTGGATGCA---------- t0032498 19

//

Mar-F-3-m0178 gi|270307433|emb|FN597044.1|:1502309:1502426:+ 118(nt) -41.73(kcal/mol)

GTTACTGTGATGTTGGCTCGGTTCACTCAGACAAAAAGAAAAAAAGAAAAAACAAACACATATATGCATGCATTTTTCTCTTTTAGCTTTGATTGAGCCGTGCCAATATCTCAGTGCC Mar-F-3-m0178 24

..(((((.(((((((((.(((((((.((((((.((((((.(((((...........................))))).)))))).).))))).))))))).))))))))).)))))..

**********TGTTGGCTCGGTTCACTCAGA*************************************************************************************** Mar-F-3-m0178-5p 24

---------ATGTTGGCTCGGTTCACTCAG---------------------------------------------------------------------------------------- t1820311 1

----------TGTTGGCTCGGTTCACTCAGA--------------------------------------------------------------------------------------- t0025730 22

----------TGTTGGCTCGGTTCACTCAGACAA------------------------------------------------------------------------------------ t2451280 1

//

Mar-F-3-m0183 gi|57903498|gb|AY728390.1|:1:91:+ 91(nt) -50.60(kcal/mol)

TGAAGACGGGGAACAGGCAGAGCATGGATGGAGCTACTAACAGAAGTACTTGTTTTGGCTCTACCCATGCACTGCCTCTTCCCTGGCTTGT Mar-F-3-m0183 21

..(((.(((((((.((((((.((((((.((((((((..(((((......))))).)))))))).)))))).)))))).))))))).)))..

******************************************************************ATGCACTGCCTCTTCCCTGGC**** Mar-F-3-m0183-3p 21

------------------------------------------------------------------ATGCACTGCCTCTTCCCTGG----- t0506480 3

------------------------------------------------------------------ATGCACTGCCTCTTCCCTGGC---- t0060257 12

-------------------------------------------------------------------TGCACTGCCTCTTCCCTGGCT--- t0307144 4

-------------------------------------------------------------------TGCACTGCCTCTTCCCTGGCTT-- t0816413 2

//

Mar-F-3-m0185 gi|57903528|gb|AY728420.1|:1:92:+ 92(nt) -36.30(kcal/mol)

CACTGGAGGCAGCGGTTCATCGATCTTTTCCTGAAGATTTTTTTGTTTTACACGAACAACACGAACCGATCGATAAACCTCTGCATCCAGTG Mar-F-3-m0185 160

(((((((.((((.((((.(((((((..(((.((.........((((((.....)))))))).)))..))))))).)))).)))).)))))))

***TGGAGGCAGCGGTTCATCGATC******************************************************************* Mar-F-3-m0185-5p 160

--CTGGAGGCAGCGGTTCATCGA--------------------------------------------------------------------- t0037427 17

--CTGGAGGCAGCGGTTCATCGAT-------------------------------------------------------------------- t0721831 2

---TGGAGGCAGCGGTTCATCGAT-------------------------------------------------------------------- t0125295 7

---TGGAGGCAGCGGTTCATCGATC------------------------------------------------------------------- t0002207 130

----GGAGGCAGCGGTTCATCGATC------------------------------------------------------------------- t0270423 4

//

Mar-F-3-m0198 gi|73847232|gb|DT456252.1|DT456252:431:528:+ 98(nt) -25.40(kcal/mol)

CTCTTATACGAAGAGTCAGATTGCATTTTGCCATCTTTATTCAATAAATGGATAAATTATTCTTTATACAATATGCAATTTGACTCCTAGTACAAAAG Mar-F-3-m0198 7

......(((...((((((((((((((.(((.....((((((((.....)))))))).((.....))..))).))))))))))))))...)))......

**********AAGAGTCAGATTGCATTTTG******************************************************************** Mar-F-3-m0198-5p 7

----------AAGAGTCAGATTGCATTTT--------------------------------------------------------------------- t0504301 3

----------AAGAGTCAGATTGCATTTTG-------------------------------------------------------------------- t0290691 4

//

Mar-F-3-m0200 gi|73858868|gb|DT461607.1|DT461607:226:288:- 63(nt) -21.20(kcal/mol)

TTAACCCACGTATGATCATCATCTATACAACTTAGAGGTGATCATGGGCCGGGCCGAGTCAGG Mar-F-3-m0200 10

....(((.(.((((((((((.((((.......)))))))))))))).)..)))..........

*********************************AGAGGTGATCATGGGCCGGG********** Mar-F-3-m0200-3p 9

--------------------------------TAGAGGTGATCATGGGCCGGG---------- t3940190 1

---------------------------------AGAGGTGATCATGGGCCGG----------- t0515954 3

---------------------------------AGAGGTGATCATGGGCCGGG---------- t0495902 3

----------------------------------GAGGTGATCATGGGCCGGGCCG------- t2930632 1

-----------------------------------AGGTGATCATGGGCCGGGCCGAG----- t6777973 1

---------------------------------------GATCATGGGCCGGGCCGAGTCAGG t5262133 1

//

Mar-F-3-m0205 gi|84171745|gb|DR460393.1|DR460393:56:185:+ 130(nt) -46.00(kcal/mol)

TTGCGGGGCACAGCCCTGGTGTTGGACATTCTTTTGCAGACGTTGTTGAAGATATTGTTGAACAAAACCCAGCAAGCATTAGCGTGCAAGGCAATGGCAAGCGTTCAATTGCAGGGCACAGCCCTGGAGT Mar-F-3-m0205 32

((.((((((...((((((..(((((((.((((.((((.(((((((.((......((((((.........)))))).)).)))))).)...)))).)).))..)))))))..))))))...)))))).)).

**********CAGCCCTGGTGTTGGACATTC*************************************************************************************************** Mar-F-3-m0205-5p 32

---------ACAGCCCTGGTGTTGGACATT---------------------------------------------------------------------------------------------------- t6354189 1

----------CAGCCCTGGTGTTGGACATTC--------------------------------------------------------------------------------------------------- t0028078 21

----------CAGCCCTGGTGTTGGACATTCT-------------------------------------------------------------------------------------------------- t5199704 1

-----------AGCCCTGGTGTTGGACATTCT-------------------------------------------------------------------------------------------------- t0304464 4

-----------AGCCCTGGTGTTGGACATTCTT------------------------------------------------------------------------------------------------- t1710927 1

------------GCCCTGGTGTTGGACATTCTT------------------------------------------------------------------------------------------------- t0431350 3

------------GCCCTGGTGTTGGACATTCTTT------------------------------------------------------------------------------------------------ t3386337 1

//

mireap-plant

Mar-S-1-m0013 gi|109869056|gb|DW498032.1|DW498032:7:319:- 313(nt) -76.70(kcal/mol)

AATAATTTGAGGGGCTGCATTGAAGTGAAGGCTGCTAATCTTTACCAGTAACGGACAAGGTTTAAACTTTCAGAACCTCTTTGTTTCTCTAAAGTAGATCATTTCTGTTTTTAGTCCATATTGGAGACGATGGAGGACATCTTTATGGACACCTTCTCATAAGGAAGGAAAAGAAAAAAAGAAGTCTAGAATTTATACAATGAGTTAGGTCAAATACGTTCTCATGGTTCGTGGGATAAAGCTGGCTGTTAGTGTTTCTTTTAGCTGTACTTGATAGTGTGATTTCAAAGGTAAGGTAGTGTCTTAAACATCG Mar-S-1-m0013 9

.....(((((((.(((((.(((...(((((..(((((..(((((((....((((((...((.((((.((..(((...(((((.(((((.(((((((((.....))))......((((.(((((....)))))..))))..)))))......((((((.....))))))...))))).)))))..)))..)))))).)).(((((...(........)...))))).)))))).)).)))))..(((((..((.....))..)))))........)))))...)))))....))).))))).))))))).....

**********GGGGCTGCATTGAAGTGAAGGCT**************************************************************************************************************************************************************************************************************************************************************************************** Mar-S-1-m0013-5p 9

--------GAGGGGCTGCATTGAAGTGAAGGC----------------------------------------------------------------------------------------------------------------------------------------------------------------------------------------------------------------------------------------------------------------------------------------- t4538549 1

----------GGGGCTGCATTGAAGTGAAGGCT---------------------------------------------------------------------------------------------------------------------------------------------------------------------------------------------------------------------------------------------------------------------------------------- t0189889 5

----------GGGGCTGCATTGAAGTGAAGGCTG--------------------------------------------------------------------------------------------------------------------------------------------------------------------------------------------------------------------------------------------------------------------------------------- t3215150 1

-----------GGGCTGCATTGAAGTGAAGGCTGC-------------------------------------------------------------------------------------------------------------------------------------------------------------------------------------------------------------------------------------------------------------------------------------- t0976825 2

//

mireap-plant

Mar-S-1-m0014 gi|109872689|gb|DW501664.1|DW501664:37:171:+ 135(nt) -49.30(kcal/mol)

CTTCGTATTCTTCCACAGCTTTCTTGAACTGCAATTTCATGTAATCAGATCATTCATTCATTCGTTGTCGTTGATGGTGCTGCATATATACGAGTACGTTGTTGCGGTTCAATAAAGCTGTGGGAAGATACAAAC Mar-S-1-m0014 8

....((((..((((((((((((.((((((((((((...(((((....((....))............((((..(((......)))....)))).))))).)))))))))))).))))))))))))..))))....

**********TTCCACAGCTTTCTTGAACTG******************************************************************************************************** Mar-S-1-m0014-5p 7

----------TTCCACAGCTTTCTTGAACT--------------------------------------------------------------------------------------------------------- t2646666 1

----------TTCCACAGCTTTCTTGAACTG-------------------------------------------------------------------------------------------------------- t0142717 6

----------------------------------------------------------------------------------------------------------------TAAAGCTGTGGGAAGATACAAA- t6119759 1

//

Mar-S-1-m0035 gi|116047830|gb|CM000348.1|:1926977:1927249:+ 273(nt) -68.99(kcal/mol)

CTGCTTCTGGACAGGACAGGACAGGACAGGACAGGTTTAAGAATATGAATATTGTTGGAAAGCTTTGCAACTGATTTGACTTCATTTTCCCGGGCCTTCTGACAGAAATTCATACAATATTATTGCTTACAACAAACATTCTATGTATTCATCATGAGTTACAATTAATTGAATACAGGCCGTGTGTTTTTTTTTTCTGCGTTACCCCCATCCAAGACCTGTCCCGCTGCGTCTTCTCTTCTCCTAGTACTTCATCCATGTCCATGTGGTGGA Mar-S-1-m0035 12

.((((...(((.((((.(((((.((.(.((((((((((..((.(((((((.((((..(((.(((..(.((.(((.......))).))..)..))).)))..))))..)))))))..........((.......((((((....((((((((...(((.......)))..))))))))......)))))).........))..........))..)))))))))).)))..))))).)))).))).)))).(((((((((....))).))))))

**********ACAGGACAGGACAGGACAGGACA************************************************************************************************************************************************************************************************************************************************ Mar-S-1-m0035-5p 12

--------GGACAGGACAGGACAGGACAGGAC------------------------------------------------------------------------------------------------------------------------------------------------------------------------------------------------------------------------------------------------- t5698413 1

----------ACAGGACAGGACAGGACAGGACA------------------------------------------------------------------------------------------------------------------------------------------------------------------------------------------------------------------------------------------------ t0123203 6

----------ACAGGACAGGACAGGACAGGACAG----------------------------------------------------------------------------------------------------------------------------------------------------------------------------------------------------------------------------------------------- t5875089 1

-----------CAGGACAGGACAGGACAGGAC------------------------------------------------------------------------------------------------------------------------------------------------------------------------------------------------------------------------------------------------- t1565700 1

-----------CAGGACAGGACAGGACAGGACAGG---------------------------------------------------------------------------------------------------------------------------------------------------------------------------------------------------------------------------------------------- t0754567 2

------------AGGACAGGACAGGACAGGACA------------------------------------------------------------------------------------------------------------------------------------------------------------------------------------------------------------------------------------------------ t3173552 1

//

Mar-S-1-m0039 gi|116047832|gb|CM000346.1|:18982831:18982930:+ 100(nt) -51.90(kcal/mol)

ATATTATATGTGCCTGGCTCCCTGTATGCCATTTGCAGAGCCCACCGGTTCTTCGATGGCCTCCGTGGATGGCGTATGAGGAGCCATGCATATTCACATG Mar-S-1-m0039 14

.......((((((.(((((((..((((((((((..(.(((.(((.(((....))).))).))).)..))))))))))..))))))).)))))).......

*********************************************************************TGGCGTATGAGGAGCCATGCA********** Mar-S-1-m0039-3p 13

---------------------------------------------------------------CGTGGATGGCGTATGAGGAGC---------------- t2184926 1

---------------------------------------------------------------------TGGCGTATGAGGAGCCATGCA---------- t0068548 9

----------------------------------------------------------------------GGCGTATGAGGAGCCATGCA---------- t1516295 1

----------------------------------------------------------------------GGCGTATGAGGAGCCATGCAT--------- t0890465 2

-----------------------------------------------------------------------GCGTATGAGGAGCCATGCA---------- t5642051 1

//

Mar-S-1-m0097 gi|189327957|gb|EQ138041.1|:914:1238:- 325(nt) -78.30(kcal/mol)

ATAAGTCTTTATATGGCTTAAAACAGGCTCCACGGGCATGGTACACTCGTCTGAGTGATTTTCTGATTTCTATTAGCTTTACTGCATCCAAGGTTGACACCTTCTTATTTATCTTATTTATTGGTGATAATATTTTTTATCTTCTGGTTTATGTTGATGACATTCTACTTACGGGTAGCAACTCTACTATGCTTCATCGGCTAATCCAGTTACTGAGTTCAGAGTTCAAGCTTCGTGACTTAGGTGTCGTTCACTATTTTTTAGGAATTAAGGTTCAACCCACTGCTATGGGTCTGATGTTACGCCAACATAAATATATTCTTAA Mar-S-1-m0097 18

.((((..(((((.((((.(((..(((((.(((..((((.((..(((((....)))))..................((......))..))..((((((.(((((..(.....(((((.....((((((................((((.(((.(((((((((((.(((((....)))))..........)))..))))))))))).))))...((((((((..(((....)))..).))))))).......)))))).....))))))..)))))))))))...)))).))))))))...))).))))..)))))......)))).

**********ATATGGCTTAAAACAGGCTCCA***************************************************************************************************************************************************************************************************************************************************************************************************** Mar-S-1-m0097-5p 18

---------TATATGGCTTAAAACAGGCTCC------------------------------------------------------------------------------------------------------------------------------------------------------------------------------------------------------------------------------------------------------------------------------------------------------ t1016514 2

---------TATATGGCTTAAAACAGGCTCCA----------------------------------------------------------------------------------------------------------------------------------------------------------------------------------------------------------------------------------------------------------------------------------------------------- t1000580 2

----------ATATGGCTTAAAACAGGCTCC------------------------------------------------------------------------------------------------------------------------------------------------------------------------------------------------------------------------------------------------------------------------------------------------------ t0978585 2

----------ATATGGCTTAAAACAGGCTCCA----------------------------------------------------------------------------------------------------------------------------------------------------------------------------------------------------------------------------------------------------------------------------------------------------- t0055568 11

-----------TATGGCTTAAAACAGGCTCC------------------------------------------------------------------------------------------------------------------------------------------------------------------------------------------------------------------------------------------------------------------------------------------------------ t2544201 1

//

Mar-S-1-m0110 gi|270241860|emb|FN595767.1|:650841:651099:- 259(nt) -45.65(kcal/mol)

AGAAACGTGTTGTGGTGCATTATTCCTTTCTCTACCACCCAAAATCTTCTTTCCACAATCATAAATATCGAAGTGTTTAAGCATTAGGTAAGACATCTCTTGATAATGATGTGAGAGATATAGACAAGTAATGTTACTAAATAACAAGAATATGTATAAAAAAAAAAAAACAGAATAAGAATACACATATTTATGTGAAAAACCTTAGACGGGAATCACCATGAGTAGAGGAGAAGAAAATTCACTATATTAAAAAATT Mar-S-1-m0110 8

..........(((((((.(((.((..((((((((((((((....(((((((((.(((.((((...(((((((((((((.(.(....).).))))).)).)))))))))))))))))))...)))..((((....)))).....(((.((((((((.............................)))))))).)))..............)))........)).)))))))))..)).))).)))))))..........

**********************************************************************************************************************************************************************************************************************************AGAGGAGAAGAAAATTCACTATA********** Mar-S-1-m0110-3p 8

-------------------------------------------------------------------------------------------------------------------------------------------------------------------------------------------------------------------------------AGTAGAGGAGAAGAAAATTCA--------------- t6227305 1

----------------------------------------------------------------------------------------------------------------------------------------------------------------------------------------------------------------------------------AGAGGAGAAGAAAATTCACT------------- t5853784 1

----------------------------------------------------------------------------------------------------------------------------------------------------------------------------------------------------------------------------------AGAGGAGAAGAAAATTCACTAT----------- t5762697 1

----------------------------------------------------------------------------------------------------------------------------------------------------------------------------------------------------------------------------------AGAGGAGAAGAAAATTCACTATA---------- t0394235 3

-----------------------------------------------------------------------------------------------------------------------------------------------------------------------------------------------------------------------------------GAGGAGAAGAAAATTCACTAT----------- t0833078 2

//

Mar-S-2-m0027 gi|164313724|gb|ES841040.1|ES841040:32:198:+ 167(nt) -33.10(kcal/mol)

CATATGCAAAATTGGTGATTGACATTTTTATCTGATTTCCACTTGTAATTATTGTCTGCAGCTTCACTTGTTTGCAAAATTTTGTCTAAAAATCTTATTTTTTTAAAATTTGATAGAATGTGGATATGAAGATAGACATTTGACACCCAGTCTGAGTTGCAATATGC Mar-S-2-m0027 8

(((((((((.(((((((.(..(.((.(((((((....(((((.........((((..((((......))))..)))).((((((((...((((.(((......))).))))))))))))))))).....))))))).)))..))).))))).....)))).))))).

**********ATTGGTGATTGACATTTTTATCT************************************************************************************************************************************** Mar-S-2-m0027-5p 7

----------ATTGGTGATTGACATTTTTAT---------------------------------------------------------------------------------------------------------------------------------------- t2835247 1

----------ATTGGTGATTGACATTTTTATC--------------------------------------------------------------------------------------------------------------------------------------- t0738862 2

----------ATTGGTGATTGACATTTTTATCT-------------------------------------------------------------------------------------------------------------------------------------- t0259409 4

------------------------------------------------------------------------------------------------------------------------TGGATATGAAGATAGACATTTGACA---------------------- t5045167 1

//

mireap-plant

Mar-S-2-m0040 gi|48793670|gb|CO094984.1|CO094984:253:354:+ 102(nt) -47.59(kcal/mol)

TCTGGAAATTTTCGGAATGGAGGAGTTGGAAAGATTTTGAATTTTCTCTGTATTTTAATAATGAAAAATCTTTCCAATTCCTCCCATTCCACTGATTTCCAG Mar-S-2-m0040 80

.(((((((((...(((((((((((((((((((((((((..(((................)))..)))))))))))))))))).)))))))...)))))))))

*************GGAATGGAGGAGTTGGAAAGA******************************************************************** Mar-S-2-m0040-5p 67

**********************************************************************TTTCCAATTCCTCCCATTCCAC********** Mar-S-2-m0040-3p 11

-----------TCGGAATGGAGGAGTTGGA------------------------------------------------------------------------ t1226980 1

-----------TCGGAATGGAGGAGTTGGAAA---------------------------------------------------------------------- t1163995 2

------------CGGAATGGAGGAGTTGGAAA---------------------------------------------------------------------- t0299119 4

------------CGGAATGGAGGAGTTGGAAAG--------------------------------------------------------------------- t0481390 3

---------------AATGGAGGAGTTGGAAAGATT------------------------------------------------------------------ t0008554 47

---------------AATGGAGGAGTTGGAAAGATTT----------------------------------------------------------------- t1936985 1

----------------ATGGAGGAGTTGGAAAGATT------------------------------------------------------------------ t0506223 3

----------------ATGGAGGAGTTGGAAAGATTT----------------------------------------------------------------- t0160845 6

-----------------TGGAGGAGTTGGAAAGATT------------------------------------------------------------------ t4666636 1

-------------------------------------TGAATTTTCTCTGTATTTTAA-------------------------------------------- t7179335 1

-------------------------------------------------------------------ATCTTTCCAATTCCTCCCAT--------------- t1279313 1

--------------------------------------------------------------------TCTTTCCAATTCCTCCCATTCC------------ t1105525 2

----------------------------------------------------------------------TTTCCAATTCCTCCCATTCCA----------- t3613672 1

----------------------------------------------------------------------TTTCCAATTCCTCCCATTCCAC---------- t0157886 6

-----------------------------------------------------------------------TTCCAATTCCTCCCATTCCA----------- t1446473 1

//

Mar-S-2-m0043 gi|48822414|gb|CO123727.1|CO123727:154:308:+ 155(nt) -40.30(kcal/mol)

GTAAATTAGTCCTTGTACGTTAGATTAAAAAGCATGTCCTTTTGTTAAAAATGTCATCCATTTCTACCGTTAAAAACTGATTTTTGCACATCAAGATGAGGTACTAGTAGAAATAACCAATATACTCTTTGATCTAATATACAGGGATTAATTTG Mar-S-2-m0043 6

.((((((((((((((((..((((((((((.((.(((.(.((((....)))).).)))..((((((((...((....((.(((((........))))).)).))...))))))))..........)).))))))))))..))))))))))))))))

*****************************************************************************************************************************TCTTTGATCTAATATACAGG********** Mar-S-2-m0043-3p 6

-----------------------------------------------------------------------------------------------------------------------------TCTTTGATCTAATATACAG----------- t2088393 1

-----------------------------------------------------------------------------------------------------------------------------TCTTTGATCTAATATACAGG---------- t0363518 3

-----------------------------------------------------------------------------------------------------------------------------TCTTTGATCTAATATACAGGGA-------- t0963138 2

//

Mar-S-2-m0046 gi|73859145|gb|DT461884.1|DT461884:212:302:- 91(nt) -28.50(kcal/mol)

TGAGATGTTAGCCAATGTCAGACATGTCTGGCACATGCGTCAGCACGGTTACATGTGTCAGTGTAAGACCTGTCTGGGACATGGTATCGAT Mar-S-2-m0046 8

...(((....((((.(((((((((.((((.((((.((.....(((((......))))))))))).)))).)))))..)))))))))))...

***********************************************************AGTGTAAGACCTGTCTGGGACA********** Mar-S-2-m0046-3p 7

----------------------------------ATGCGTCAGCACGGTTACATGTGT--------------------------------- t7879814 1

---------------------------------------------------------TCAGTGTAAGACCTGTCTGGGACA---------- t7369762 1

----------------------------------------------------------CAGTGTAAGACCTGTCTGGGACA---------- t5003206 1

-----------------------------------------------------------AGTGTAAGACCTGTCTGGGACA---------- t0438524 3

-----------------------------------------------------------AGTGTAAGACCTGTCTGGGACATG-------- t0789208 2

//

Mar-S-2-m0051 gi|84173350|gb|DR461998.1|DR461998:108:223:+ 116(nt) -30.02(kcal/mol)

CCCGGGAGGAATTCGATCTCTTCAATCTCGGGATACCACCTAAATAACTGCGGCCAAAAAGTCAAATAGGTCGGGAAGAAAGAGTCTGAGGTTGGGTCGGACGACATACATCTTGG Mar-S-2-m0051 6

.((((((.....(((.(((.((((((((((((...(((((((....(((..........)))....))))).))..........))))))))))))..))))))......))))))

*************************************************************************************CTGAGGTTGGGTCGGACGACA********** Mar-S-2-m0051-3p 6

------------------------------------------------------------------------------------TCTGAGGTTGGGTCGGACGAC----------- t6113066 1

-------------------------------------------------------------------------------------CTGAGGTTGGGTCGGACGAC----------- t4457664 1

-------------------------------------------------------------------------------------CTGAGGTTGGGTCGGACGACA---------- t0370880 3

-------------------------------------------------------------------------------------CTGAGGTTGGGTCGGACGACAT--------- t3835116 1

//

mireap-plant

Mar-S-3-m0014 gi|109874652|gb|DW503626.1|DW503626:79:186:- 108(nt) -30.00(kcal/mol)

TTCAACAACATCCATGGTGGAGATTGCTCTTTTGAAGCATATGCTGTCAATAACCATGATGGTATTAGGTTTATTGAAGAATATGTCCATGATGATGATGATAGCTTG Mar-S-3-m0014 7

........(((((((.(((((.((...(((((..((((..(((((((((.......)))))))))...))))...)))))..)).))))).)))..))))........

**********TCCATGGTGGAGATTGCTCTT***************************************************************************** Mar-S-3-m0014-5p 6

---------ATCCATGGTGGAGATTGCTCT------------------------------------------------------------------------------ t5006213 1

----------TCCATGGTGGAGATTGCTC------------------------------------------------------------------------------- t1022906 2

----------TCCATGGTGGAGATTGCTCTT----------------------------------------------------------------------------- t0442165 3

--------------------------------------ATATGCTGTCAATAACCATGA------------------------------------------------- t7014314 1

//

Mar-S-3-m0041 gi|48808281|gb|CO109595.1|CO109595:402:570:- 169(nt) -39.70(kcal/mol)

TCGATGGGTTTGTTGAAGGTCGATGGGTTAACTGATAATACATGGATGGAACGTGCGGTTCGTTGCTTTGATTATGATTATGATAATAATTATGAAATTGAAACTCCCTTGATGATGATGAACAAGAACCTTAATTATGCCCATATGGTGTTCGACGAAGACCCTTTTA Mar-S-3-m0041 17

.....((((((((((((..(((((((((....((((....((((.......)))).(((((.(((.(((..((((((((((....)))))))))).((..(.......)..))......)))))))))))...)))).)))))).)))..)))))))..))))).....

**********TGTTGAAGGTCGATGGGTTAA****************************************************************************************************************************************** Mar-S-3-m0041-5p 16

---------TTGTTGAAGGTCGATGGGTTA------------------------------------------------------------------------------------------------------------------------------------------- t0135773 7

----------TGTTGAAGGTCGATGGGTTAA------------------------------------------------------------------------------------------------------------------------------------------ t0108801 8

-----------GTTGAAGGTCGATGGGTTAAC----------------------------------------------------------------------------------------------------------------------------------------- t6091148 1

--------------------------------------TACATGGATGGAACGTGCGGT-------------------------------------------------------------------------------------------------------------- t5376089 1

//
